# Phenotypically distinct female castes in honey bees are defined by alternative chromatin states during larval development

**DOI:** 10.1101/gr.236497.118

**Published:** 2018-10

**Authors:** Marek Wojciechowski, Robert Lowe, Joanna Maleszka, Danyal Conn, Ryszard Maleszka, Paul J. Hurd

**Affiliations:** 1 School of Biological and Chemical Sciences, Queen Mary University of London, London E1 4NS, United Kingdom;; 2 The Blizard Institute, Barts and The London School of Medicine and Dentistry, Queen Mary University of London, London E1 2AT, United Kingdom;; 3 Research School of Biology, Australian National University, Canberra ACT 2601, Australia

## Abstract

The capacity of the honey bee to produce three phenotypically distinct organisms (two female castes; queens and sterile workers, and haploid male drones) from one genotype represents one of the most remarkable examples of developmental plasticity in any phylum. The queen–worker morphological and reproductive divide is environmentally controlled during post-embryonic development by differential feeding. Previous studies implicated metabolic flux acting via epigenetic regulation, in particular DNA methylation and microRNAs, in establishing distinct patterns of gene expression underlying caste-specific developmental trajectories. We produce the first genome-wide maps of chromatin structure in the honey bee at a key larval stage in which developmental canalization into queen or worker is virtually irreversible. We find extensive genome-wide differences in H3K4me3, H3K27ac, and H3K36me3, many of which correlate with caste-specific transcription. Furthermore, we identify H3K27ac as a key chromatin modification, with caste-specific regions of intronic H3K27ac directing the worker caste. These regions may harbor the first examples of caste-specific enhancer elements in the honey bee. Our results demonstrate a key role for chromatin modifications in the establishment and maintenance of caste-specific transcriptional programs in the honey bee. We show that at 96 h of larval growth, the queen-specific chromatin pattern is already established, whereas the worker determination is not, thus providing experimental support for the perceived timing of this critical point in developmental heterochrony in two types of honey bee females. In a broader context, our study provides novel data on environmentally regulated organismal plasticity and the molecular foundation of the evolutionary origins of eusociality.

Eusociality is an intriguing evolutionary invention found in many species of Hymenoptera (e.g., bees, wasps, and ants), termites, and even some mammals (Jarvis 1981; Gordon 2002; Nalepa 2015). In true eusocialism, large self-organizing colonies are formed out of individuals partitioned into reproductive and nonreproductive types known as castes, each representing an organism with a distinct repertoire of morphological, physiological, and behavioral characteristics. In some species, this phenotypic divide is epigenetically rather than genetically determined, consistent with the environmental impact by which these differences are implemented.

Insect pollinators such as the honey bee (*Apis mellifera*) play a crucial role in most ecosystems and strongly influence ecological relationships, for example, by helping to maintain genetic variation in flowering plants. Furthermore, in farmed areas, the honey bee is used extensively for the commercial pollination of a variety of cultivated crops. Honey bees live in complex societies comprising tens of thousands of individuals, in which there is a division of labor that can be separated into two broad categories. The first is a reproductive distinction; each colony contains two diploid female castes comprising a single queen who is specialized for reproduction and thousands of sterile female worker bees (Winston 1991). The second distinction relates to the division of tasks performed by the worker caste, which changes during the course of adult life from nurse through to forager, in a process termed behavioral maturation that results in worker subcastes (Winston 1991). A third main phenotypic outcome, which develops from unfertilized eggs, is a haploid male drone. The key feature in the establishment of these different female developmental trajectories and subsequent maintenance during adulthood, is nutrition. For the first 72 h after hatching, both queen and worker larvae receive a certain amount of nutritious jelly, although the worker jelly contains lower concentration of sugars and a few other ingredients than the queen food known as royal jelly (Wang et al. 2016; Maleszka 2018). Drone larvae not only receive a distinct diet, but also in larger quantities compared to that of worker larvae. This suggests that similarly to queens, nutrition provides important cues for their proper development (Hrassnigg and Crailsham 2007). Larvae developmentally destined to be workers or drones are then switched to a diet comprised of nectar and pollen, in contrast to larvae destined to become queens, which remain on a royal jelly diet. After 96 h, larval chambers are capped and no further feeding occurs until after pupation. Differential feeding continues throughout adulthood, resulting in distinct but genetically indistinguishable organisms/castes. The honey bee genome therefore exemplifies environmentally driven phenotypic plasticity, where diet dictates the ability of different phenotypes to arise from a single genome and represents one of the most striking examples of developmental plasticity in any phylum.

The establishment, maintenance, and modulation of transcriptional programs such as those during development are reliant on the inherent plasticity of chromatin, and recent evidence indicates that chromatin-based epigenetic mechanisms direct nutrition-mediated caste differentiation in the honey bee. RNAi knock-down of the putative de novo DNA methyltransferase *DNMT3* in newly hatched larvae has been shown to lead to royal jelly–like effects on developmental trajectory, resulting in a significantly high proportion of queens with fully developed ovaries (Kucharski et al. 2008). The potential role of differential DNA methylation in influencing alternate developmental outcomes of queens and workers has been confirmed by genome-wide mapping of methylated CpGs in both castes at 96 h of larval growth (Foret et al. 2012). Although the exact function of this common epigenomic modification in the honey bee remains poorly understood, several studies have shown that differential DNA methylation correlates with alternative splicing and modulation of gene expression in a context-dependent manner (Lyko et al. 2010; Foret et al. 2012; Kucharski et al. 2016; Wedd et al. 2016). More recently, using proteomic approaches, we demonstrated that honey bee histone proteins are extensively post-translationally modified and show caste-specific signatures (Dickman et al. 2013). Given the conservation of both histone sequences and epigenetic machinery in the honey bee, we hypothesize that histone post-translational modifications (PTM) are also pivotal in determining developmental trajectory in response to nutrition in this organism. Furthermore, a direct link between a component of royal jelly and potential caste-specific histone PTM changes has been provided by a biochemical study of a fatty acid, (*E*)-10-hydroxy-2-decenoic acid (10-HDA), which comprises up to 5% of royal jelly. 10-HDA has been shown to be a histone deacetylase inhibitor and can reactivate the expression of epigenetically silenced genes in mammalian cells (Spannhoff et al. 2011).

Because hundreds of genes have been implicated in queen–worker differentiation (Barchuk et al. 2007; Foret et al. 2012), we reasoned that their coordinated differential expression has to be regulated at the level of chromatin. Chromatin structure has not previously been studied in the honey bee; for the first time, we have determined the genome-wide distribution of three histone H3 modifications (H3K4me3, H3K27ac, and H3K36me3), in both queen and worker female castes, at a crucial development time point that has been shown to be critical for caste determination with both types of females essentially committed to a specific trajectory (Weaver 1966; Maleszka 2018). We sequenced chromatin immunoprecipitation (ChIP) and RNA samples from 96 h larval heads and identified thousands of genomic regions that show caste-specific chromatin states, many of which are linked to caste-specific gene transcription.

## Results

### Histone post-translational modifications in the honey bee associate with transcribed regions

**Figure 1.**
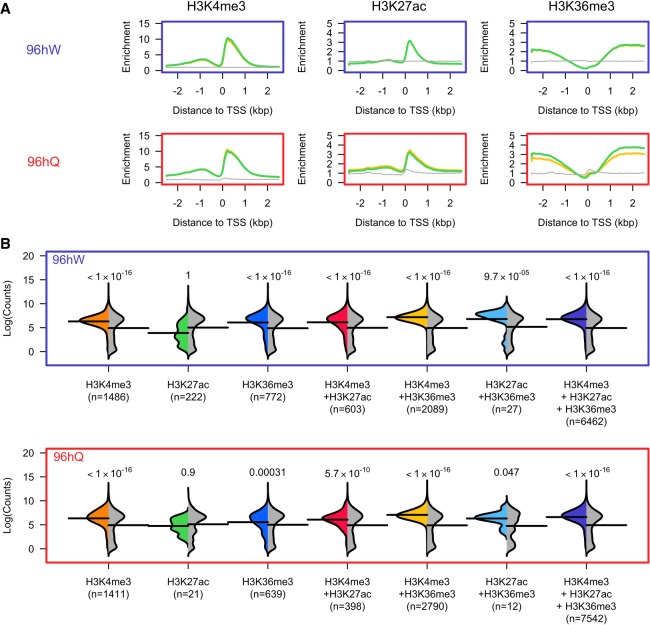
H3K4me3, H3K27ac, and H3K36me3 are associated with transcribed regions in honey bee castes. (*A*) Plots of the average ChIP-seq enrichment above input around the TSS (±2 kbp) of genes profiled across 96hW (*upper*) and 96hQ (*lower*). The green and yellow lines represent the two replicates performed for each ChIP-seq experiment, and the gray line represents the input. (*B*) The expression distribution (shown by a colored half-bean) of transcripts enriched by greater than threefold change above input for all possible combinations of H3K4me3, H3K27ac, and H3K36me3, compared to a random sampling of genes (gray half-bean). The mean of either distribution is shown by a solid black line; 96hW is shown in the *top* panel; 96hQ in the *bottom* panel; n is the number of transcripts.

We previously identified more than 20 different histone PTM states in queen and worker honey bee castes using mass spectrometry (Dickman et al. 2013; M Dickman and P Hurd, unpubl.). To study chromatin structure in honey bees, for the first time we determined the genome-wide distribution of three histone PTMs that are associated with transcription and active *cis*-elements in other organisms: H3K4me3, H3K27ac, and H3K36me3 (Pokholok et al. 2005; Guenther et al. 2007; Heintzman et al. 2007; Négre et al. 2011; Simola et al. 2013). We profiled replicate pools of worker (W) and queen (Q) larval heads at 96 h post-hatching by ChIP-seq (*n* = 50 per caste). Replicates show a very strong and significant correlation across all histone PTMs and castes (*ρ* > 0.96; *P*-value < 2.2 × 10^−16^) (Supplemental Fig. S1). We find enrichment of H3K4me3 and H3K27ac and depletion of H3K36me3 around the transcriptional start sites (TSS) of genes in both 96hW and 96hQ castes (Fig. 1A; Supplemental Fig. S2), similar to what was previously reported in other organisms including vertebrates, invertebrates, and plants (Pokholok et al. 2005; Guenther et al. 2007; Heintzman et al. 2007; Négre et al. 2011; Simola et al. 2013). H3K36me3 is mostly found downstream from TSSs (Fig. 1A), suggesting that it demarcates gene bodies. Overall, the majority of the 10,746 protein coding genes in the honey bee show high levels of enrichment (greater than threefold over input) for at least one of the three histone PTMs profiled (57.6% in 96hW and 61.3% in 96hQ). Because each of these histone PTMs are mostly localized close to or within genes, we next investigated their correlation with transcription. We profiled gene expression in 96hW and 96hQ larval heads (*n* = 4 per caste) by RNA-seq. In both 96hW and 96hQ castes, genes marked uniquely by H3K4me3 or H3K36me3 show a significant (*P*-value < 3.1 × 10^−4^) increase in expression compared to background, whereas genes marked uniquely by H3K27ac do not show any significant association with expression (Fig. 1B). Furthermore, in both castes, genes marked by two or more histone PTMs also show significant increases in expression compared to background (*P*-value < 0.047). Thus, in both worker and queen honey bee castes; H3K4me3, H3K36me3, and H3K27ac when occurring in combination, associate with actively transcribed regions and therefore have the potential to regulate caste-specific gene expression.

### Caste-specific chromatin patterns correlate with differential gene expression

**Figure 2.**
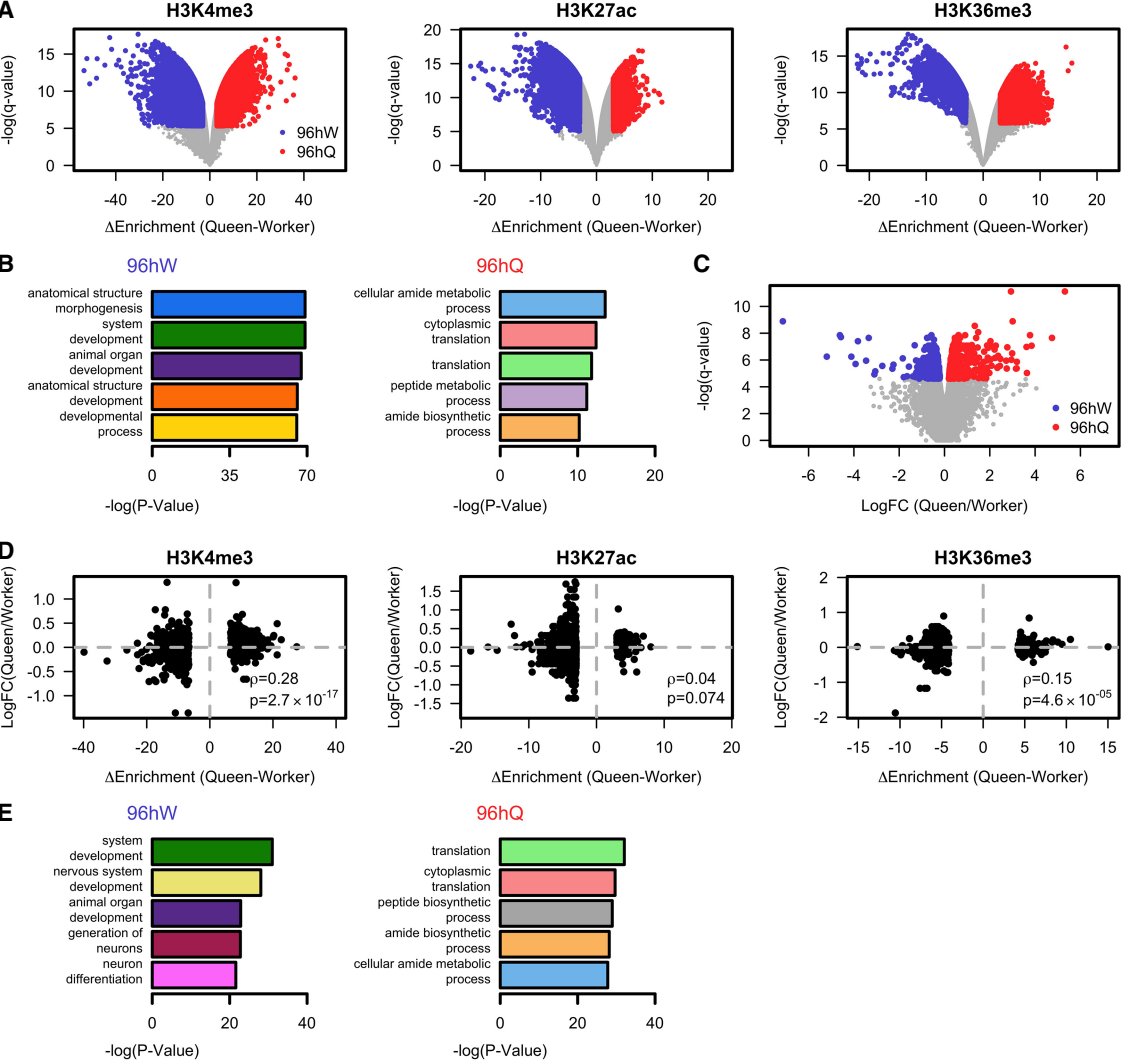
At 96 h, worker and queen larvae show caste-specific differences in the enrichment of H3K4me3, H3K27ac, and H3K36me3 that correlate with differential gene expression. (*A*) A volcano plot of the difference in enrichment between 96hW and 96hQ castes against the negative log *q*-value for H3K4me3, H3K27ac, and H3K36me3. Regions in gray fall below the genome-wide threshold of significance (*P* > 0.01). Regions in blue (96hW) and red (96hQ) are those that reach genome-wide significance (*P* ≤ 0.01) and have a greater than threefold difference in enrichment above input between castes. (*B*) The negative log *P*-value for the top five biological process GO terms for those genes which show increased enrichment for H3K4me3, H3K27ac, or H3K36me3 in 96hW compared to 96hQ (*left*) and for those which show an increased enrichment in 96hQ compared to 96hW (*right*). (*C*) A volcano plot of the log fold change (LogFC) in transcript expression by RNA-seq between 96hW and 96hQ castes against the negative log *q*-value. Transcripts in gray fall below the genome-wide threshold of significance (*P* > 0.01); transcripts in blue reach genome-wide significance (*P* ≤ 0.01) and are more expressed in 96hW; transcripts in red reach genome-wide significance (*P* ≤ 0.01) and are more expressed in 96hQ. (*D*) A scatter plot of the difference in significant ChIP-seq enrichment between 96hQ and 96hW (*x*-axis) against the LogFC of transcript expression between 96hQ and 96hW castes (*y*-axis). (*E*) The negative log *P*-value for the top five biological process GO terms for those transcripts that show both increased expression and increased enrichment in H3K4me3, H3K27ac, or H3K36me3 in 96hQ compared to 96hW (*left*) and for those that show both increased expression and increased enrichment in 96hQ compared to 96hW (*right*).

Having established that within each caste, H3K4me3, H3K27ac, and H3K36me3 are significantly enriched at transcribed regions, we next wanted to determine whether these three histone PTMs show caste-specific distributions. To investigate this, we called differences between 96hW and 96hQ castes for H3K4me3, H3K27ac, and H3K36me3 (Fig. 2A). For H3K4me3, we identify 1834 unique genomic regions that are significantly more enriched in 96hW and 3333 in 96hQ (adjusted *P*-value <0.01 and |Δ*Enrichment*| > 3). For H3K27ac, we identify 2027 unique genomic regions that are significantly more enriched in 96hW and 489 in 96hQ (adjusted *P*-value <0.01 and |Δ*Enrichment*| > 3). Finally, for H3K36me3 we identify 1196 unique regions that are significantly more enriched in 96hW and 3285 in 96hQ (adjusted *P*-value <0.01 and |Δ*Enrichment*| > 3). Gene Ontology (GO) analysis of those genes that show any significant caste-specific chromatin marks reveal a distinct developmental separation of castes at 96 h. In 96hQ, overrepresented GO terms significantly associate with physio-metabolic functions and processes, such as the structural constituents of ribosome (GO:0003735) and biological processes, including cellular amide metabolic processes (GO:0043603), cytoplasmic translation (GO:0002181), and peptide metabolic processes (GO:0006518), suggesting that at 96 h, the queen developmental trajectory is established (Fig. 2B; Supplemental Table S1). In contrast, 96hW GO terms associate with development and transcriptional programming, including the molecular functions of transcription factor activity and binding (GO:0003700) along with biological processes of anatomical structure morphogenesis (GO:0009653), system development (GO:0048731), and developmental processes (GO:0032502). Importantly, this suggests that relative to the 96hQ, the worker caste developmental trajectory is not yet established at 96 h (Fig. 2B; Supplemental Fig. S3; Supplemental Table S2). Because caste-specific DNA methylation patterns were previously reported (Lyko et al. 2010; Foret et al. 2012), we asked whether the observed caste-specific differences in histone PTM enrichment correlated with differentially DNA methylated positions (DMPs). We reanalyzed the 96 h larval DNA methylation data of Foret et al. (2012) using Fisher's exact test and detected a total of 24,663 DMPs (adjusted BH *P*-value <0.05) and then analyzed the enrichment of these DMPs with each of our differential histone PTMs. We find only a strong enrichment in H3K36me3 (permutation test; *P*-value <0.001; Fold Enrichment = 15). However, this is driven by an overlap in genome distribution rather than a strong functional association (Supplemental Figs. S4, S5). This suggests that DNA methylation and histone PTMs provide different signals in the process of caste determination.

**Figure 3.**
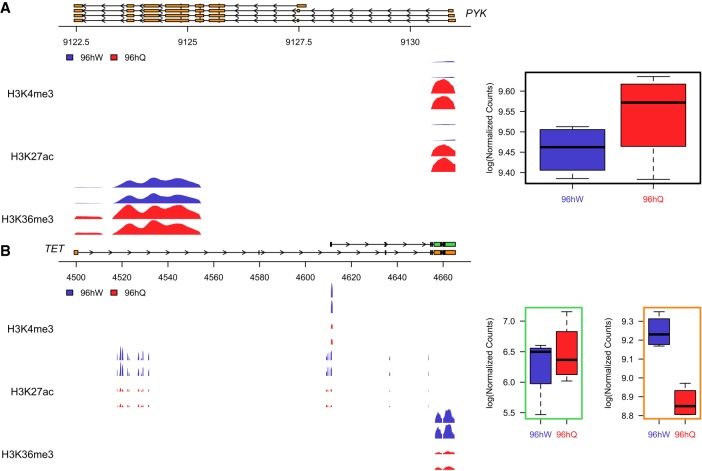
Profiles of two genes that show a significant difference in both ChIP-seq enrichment and transcript expression between castes. (*A*) Pyruvate kinase (*PYK*; LOC552007). Regions are shown which reach genome-wide significance (*P* ≤ 0.01) and have a greater than threefold difference in enrichment over input between 96hW (blue) and 96hQ (red). The expression profile is shown in the *right* panel. (*B*) Ten-eleven translocation (*TET*; LOC412878). Regions are shown which reach genome-wide significance (*P* ≤ 0.01) and have a greater than threefold difference in enrichment over input between 96hW (blue) and 96hQ (red) for both *TET* transcripts, XM_016915488 (*upper* transcript) and XM_006561197 (*lower* transcript). The expression profile is also shown for transcripts XM_016915488 (*left*) and XM_006561197 (*right*).

We next wanted to determine whether the observed caste-specific differences in histone PTM enrichment correlated with any differential gene expression between castes. Unsupervised multidimensional scaling analysis of our RNA-seq data reveal a strong separation of the two castes (Supplemental Fig. S6). We identify a total of 1060 significant differences (genome-wide adjusted *P*-value <0.01) in transcript levels between 96hW and 96hQ (Fig. 2C), with 386 transcripts showing an increase in expression in the 96hW and 674 showing an increase in the 96hQ (Supplemental Fig. S7; Supplemental Tables S3, S4). To confirm that we were robustly measuring differences between castes, we compared these differentially expressed transcripts to previously published RNA-seq data taken from whole 96hW and 96hQ larvae (Supplemental Fig. S8; Ashby et al. 2016). We find a strong correlation (*ρ* = 0.51; *P*-value <5.3 × 10^−72^) between the transcriptional differences detected in our experiment and that of Ashby et al. (2016). We find that genes differentially enriched with H3K4me3 (*ρ* = 0.28; *P*-value = 2.7 × 10^−17^) and H3K36me3 (*ρ* = 0.15; *P*-value = 4.6 × 10^−5^) show significant correlation with transcriptional differences, suggesting that caste-specific H3K4me3 and H3K36me3 patterns associate with caste-specific transcriptional profiles (Fig. 2D). In contrast, genes differentially enriched with H3K27ac show a nonsignificant correlation with caste-specific transcriptional differences (*ρ* = 0.04; *P*-value = 0.074). To investigate the function of genes that show consistent caste-specific changes in both gene expression and histone PTM enrichment, we performed Gene Ontology analysis. We observe that these genes reveal a distinct developmental separation of castes at 96 h. In 96hQ, we again find significant GO terms for biological processes and functions that associate with physio-metabolic processes such as translation (GO:0006412 and GO:0002181) and peptide biosynthetic processes (GO:0043043) (Fig. 2E; Supplemental Table S5). In contrast, GO terms for system development (GO:0048731), nervous system development (GO:0007399), generation of neurons (GO:0048699), and neuron differentiation (GO:0030182) are enriched in 96hW (Fig. 2E; Supplemental Fig. S9; Supplemental Table S6). In agreement with our earlier analyses, this again suggests that at 96 h and relative to worker, queen development is set, whereas the worker-specific developmental program (which will determine a distinct phenotype) is yet to be established. Representative examples of two genes that show some of the most significant caste-specific changes in both gene expression and histone PTM enrichment are shown in Figure 3. Pyruvate kinase (*PYK*; LOC552007) is shown as an example of a physio-metabolic 96hQ-specific gene, where H3K27ac and H3K4me3 enrichment differences associate with the TSS and H3K36me3 over the gene body (Fig. 3A; Supplemental Fig. S10). Similarly, the 96hW-specific gene Ten-eleven translocation (*TET*; LOC412878), also shows TSS and gene body differences in H3K4me3, H3K27ac, and H3K36me3. However, *TET* also has significant 96hW-specific differences in H3K27ac in the intronic region between two alternative TSSs (Fig. 3B). Notably, the longer transcript containing caste-specific intronic H3K27ac (XM_006561197) shows not only the highest level of expression but also the most significant caste-specific difference in expression (LogFC = 0.38; *P*-value = 0.0075). Taken together, these data indicate that aged-matched worker and queen honey bee castes exhibit distinct patterns of histone PTMs. Furthermore, patterns of H3K4me3 and H3K36me3 correlate with caste-specific gene expression and reveal that at this crucial time point and relative to the queen, the worker-specific developmental pathway is not yet established.

### Intronic H3K27ac regions most readily define the worker caste and are enriched for transcription factor binding sites

**Figure 4.**
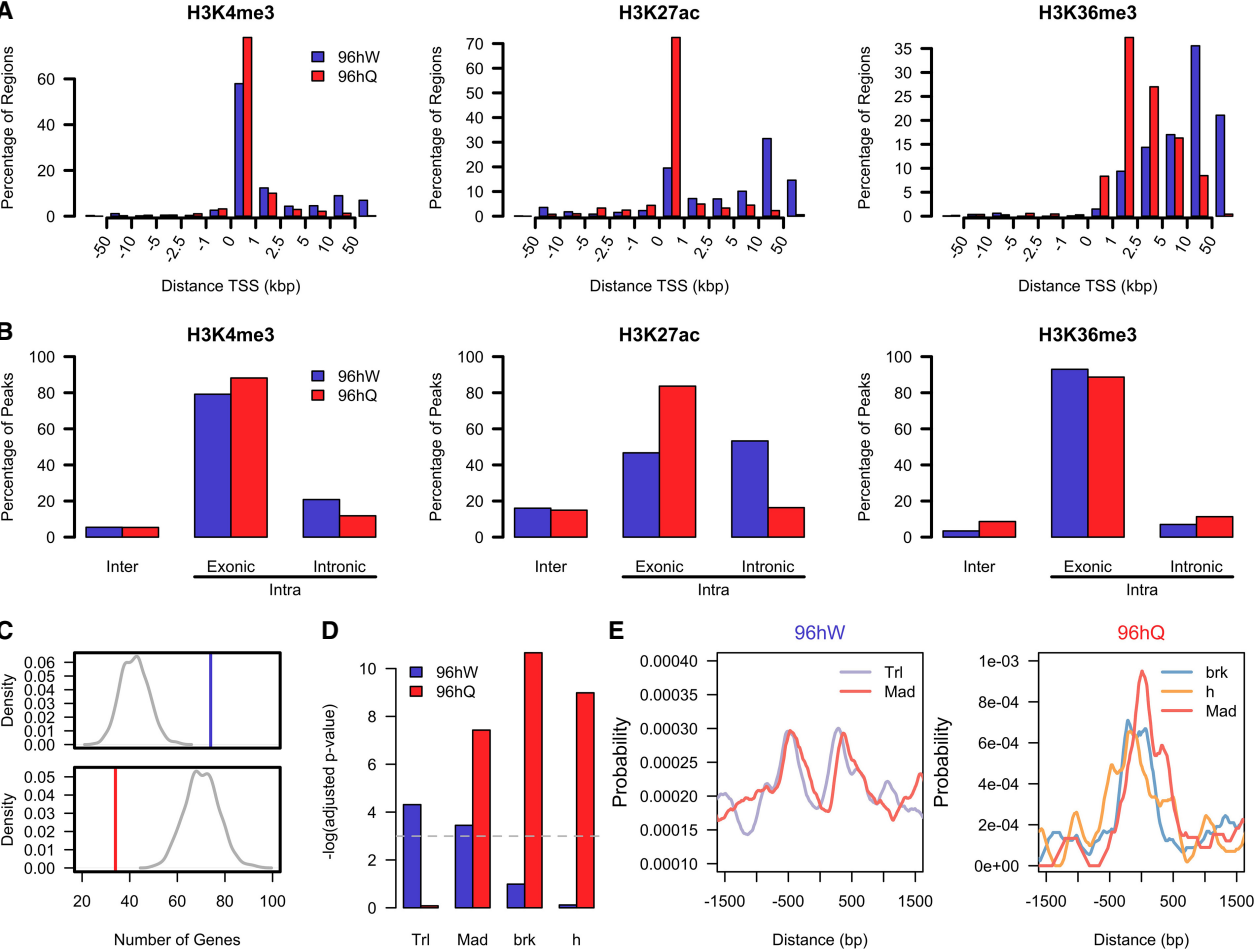
Intronic H3K27ac regions define the worker caste and are enriched for transcription factor binding sites. (*A*) A bar plot showing the percentage of unique H3K4me3, H3K27ac, and H3K36me3 ChIP-seq regions and their location relative to the nearest TSS in 96hW (blue) and 96hQ (red). (*B*) A bar plot showing the percentage of unique H3K4me3, H3K27ac, and H3K36me3 ChIP-seq regions within intergenic or intragenic locations. Intragenic distributions are further defined as either exonic or intronic. The blue bar represents regions with higher enrichment in 96hW, and the red bar are those regions with higher enrichment in 96hQ. (*C*) In the *top* panel, the vertical blue line shows the number of differentially expressed genes that contain at least one peak of intronic H3K27ac and are more expressed in 96hW compared to 96hQ. A background distribution (shown in gray) was calculated by randomly selecting an identical number of genes and calculating how many of these contain at least one peak of intronic H3K27ac. This was repeated 1000 times. In the *bottom* panel, the vertical red line shows the number of differentially expressed genes that contain at least one peak of intronic H3K27ac and are more expressed in 96hQ compared to 96hW. A background distribution (shown in gray) was calculated as previously and repeated 1000 times. (*D*) A bar plot of the adjusted *P*-value for enrichment of transcription factor binding motifs located within caste-specific intronic H3K27ac regions. (*E*) A motif probability graph showing the probability of transcription factor binding motifs in relation to caste-specific intronic H3K27ac (centered at 0 bp).

Previously, we observed a strong correlation between caste-specific H3K4me3 and H3K36me3 patterns and transcriptional profiles but not for H3K27ac. However, at the *TET* locus, an alternative longer transcript containing 96hW-specific intronic H3K27ac did correlate strongly with caste-specific transcription, whereas a shorter transcript with caste-specific H3K27ac around the TSS did not. This led us to examine more closely the distribution of H3K27ac caste differences, and to this end, we plotted unique ChIP-seq regions relative to TSSs. In both castes, the majority of unique H3K4me3 regions are similarly located around the TSS. For H3K36me3, the majority of unique regions are in gene bodies, with the distribution in 96hW more downstream than 96hQ. In contrast, differences in H3K27ac have a much more pronounced caste-specific distribution (Fig. 4A). An increase in enrichment of H3K27ac in 96hQ is almost exclusively located within 0–1 kbp of TSSs, whereas in 96hW, enrichment is mostly located outside these regions (Fig. 4A). Furthermore, in comparison to H3K4me3 and H3K36me3, there is significantly more caste-specific intergenic H3K27ac (16% in 96hW and 15% in 96hQ) (Fig. 4B). In order to further define the caste-specific distribution of intragenic H3K27ac, we mapped differences to either exons or introns. We observe a difference in the location of 96hW and 96hQ-specific intragenic H3K27ac, with 83% of all 96hQ-specific enrichments occurring within exons; conversely, >53% of 96hW-specific differences occur within introns (Fig. 4B). We then asked whether there was any correlation between these distinct caste-specific intronic H3K27ac regions and expression of the associated gene. In agreement with our previous observations at the *TET* locus, genes that are significantly more expressed in 96hW show highly significant enrichment for 96hW intronic H3K27ac (1.75-fold enrichment; *P*-value <0.001), whereas those genes significantly more expressed in 96hQ are actually depleted for 96hQ intronic H3K27ac (2.06-fold depletion; *P*-value <0.001) (Fig. 4C). However, in both castes, intronic H3K27ac is associated with gene expression (Supplemental Fig. S11). It is also likely that H3K27ac acts at distal regulatory elements; therefore, we determined the average expression of genes at various distances from H3K27ac enriched regions, revealing a specific peak of gene expression at a distance of 130–140 kbp (Supplemental Figs. S12, S13). In order to try and gain a better understanding of the functional significance of caste-specific intronic H3K27ac, we performed motif enrichment analysis on these regions using CentriMo from the MEME suite software package (Bailey and MacHanick 2012). Using transcription factor motifs annotated in *Drosophila melanogaster*, we identify highly significant enrichment for Trithorax-like (Trl; *P*-value <1.3 × 10^−2^) and Mothers against dpp (Mad; *P*-value <3.2 × 10^−2^), which accounts for 70% of all 96hW-specific regions of intronic H3K27ac (Fig. 4D). In contrast, 66% of all 96hQ-specific intronic H3K27ac regions are enriched in motifs for Brinker (brk; *P*-value <2.3 × 10^−5^), Hairy (h; *P*-value <1.3 × 10^−4^), and Mothers against dpp (Mad; *P*-value <5.9 × 10^−4^) (Fig. 4D). Analysis of Trl and Mad binding sites in relation to peaks of intronic 96hW-specific H3K27ac reveals highly significant motif enrichment flanking peaks of H3K27ac, most likely a reflection of nucleosome displacement associated with transcription factor binding (Fig. 4E). Conversely, binding sites for brk, h, and Mad are centered on peaks of 96hQ-specific H3K27ac (Fig. 4E). Given the enrichment for Trl and Mad transcription factor binding sites, the presence of the enhancer-associated histone modification H3K27ac, the intronic genomic locations, and increased gene expression, these results suggest that 96hW-specific H3K27ac enriched regions are marking active enhancers and play an important role in worker and queen honey bee caste determination.

## Discussion

We used ChIP-seq to provide the first description of genome-wide caste-specific chromatin patterns in the honey bee and, furthermore, the first between Hymenoptera castes that show a reproductive division of labor. Combined with RNA-seq analysis and at a crucial developmental stage when developmental trajectory has been shown to be irreversible (Weaver 1966; Maleszka 2018), we identify numerous queen and worker-specific chromatin differences many of which correlate with caste-specific transcription. Importantly, regions of the genome that show the most robust caste-specific differences are suggestive of previously unidentified enhancer regions that are important in specifying the worker caste development from that of the queen.

We hypothesized that for the honey bee genome to specify two different female castes, different chromatin patterns and transcriptional programs have to be established during development. Previous work mainly focused on the role of caste-specific DNA methylation patterns in adult honey bees (Lyko et al. 2010; Welsh et al. 2017), in which differentially methylated regions are mainly localized to exons and are thought to mediate alternative splicing (Foret et al. 2012; Kucharski et al. 2016). More recently, proteomic analysis of histone modifications (Dickman et al. 2013) and miRNAs (Ashby et al. 2016) also suggested potential roles in caste determination. Because chromatin structure was not previously studied in the honey bee, we first established that within each caste H3K4me3, H3K27ac, and H3K36me3 were associated with transcribed regions in a manner consistent in other organisms. Importantly, this also suggested that these highly conserved histone modifications could have the potential to regulate the widely reported caste-specific transcription of the honey bee genome (Evans et al. 1999; Evans and Wheeler 2001; Barchuk et al. 2007; Chen et al. 2012; Foret et al. 2012; Cameron et al. 2013; Ashby et al. 2016). We find that in both worker and queen castes, H3K4me3 and H3K36me3 strongly correlate with transcription, whereas H3K27ac alone does not. Importantly, worker and queen castes have contrasting chromatin patterns for all three histone PTMs at 96 h post-hatching, and these genomic regions are strongly suggestive of fundamentally different caste developmental states. In queen, enrichment for genes involved in body growth suggests that developmental trajectory is established, whereas in worker, there is a strong enrichment for processes concerned with continued development and specialization. This is further supported by transcriptome analysis, which also shows strong developmental separation of worker and queen caste at 96 h in agreement with other studies (Barchuk et al. 2007; Ashby et al. 2016). Moreover, we find that for H3K4me3 and H3K36me3, caste-specific chromatin signatures correlate with caste-specific transcription suggesting that histone PTMs play a role in determining alternate developmental trajectories. Furthermore, analysis of these regions again highlights contrasting caste-specific developmental stages at 96 h. Biological processes associated with body growth in the queen is in sharp contrast to worker development, where neurogenesis is strongly evident. This is consistent with previous observations that queen and worker development is distinct for neurogenesis (Ashby et al. 2016), possibly because workers show remarkable behavioral complexity in adult life and would therefore be expected to require a more complex nervous system.

In contrast to H3K4me3 and H3K36me3, we show that caste-specific H3K27ac does not correlate with caste-specific transcriptional differences. Although the distribution of caste-specific differences for H3K4me3 and H3K36me3 occurs mainly over similar genomic locations, H3K27ac shows a much more pronounced caste bias. Queen-specific H3K27ac is localized mainly within exons and close to transcriptional start sites; conversely, worker-specific H3K27ac is more pervasive and most frequently located within introns. Furthermore, genes with caste-specific regions of intronic H3K27ac correlate with higher levels of caste-specific expression suggesting that these regions may play important *cis*-regulatory roles. Enhancers are *cis*-acting elements that are frequently found in noncoding regions of genomes and are characterized by nucleosome-free regions enriched in transcription factor binding sites (Calo and Wysocka 2013; Li et al. 2016). Activation of enhancers most commonly requires the repositioning of nucleosomes through the activity of ATP-dependent chromatin remodelers followed by transcription factor binding and recruitment of coactivators, which modify adjacent nucleosomes most often at H3K27ac (Creyghton et al. 2010; Rada-Iglesias et al. 2011). The transcriptional coactivator CREB binding protein (CBP) is the main histone acetyltransferase that catalyzes H3K27ac (Tie et al. 2009; Jin et al. 2011), and honey bees have a single gene for the CBP enzyme (LOC726332) that is differentially DNA methylated in *A. mellifera* larvae (Foret et al. 2012). H3K27ac, transcription factor binding motifs, and CBP occupancy have been widely used to successfully map enhancers in numerous cell types, tissues, and organisms (Visel et al. 2009; Négre et al. 2011; Rada-Iglesias et al. 2011; Simola et al. 2013; Koenecke et al. 2016).

Therefore, in the absence of any previous enhancer annotation in the honey bee genome, we analyzed regions of caste-specific intronic H3K27ac for conserved transcription factor binding motifs. Trl (LOC552090; GAGA factor in mammals) and Mad (LOC409301; SMAD1 in mammals) motifs accounted for 70% of all worker-specific intronic H3K27ac. Trl/GAGA factor is a multifunctional transcriptional regulator and the gene is differentially DNA methylated in *A. mellifera* larvae (Foret et al. 2012). Trl/GAGA factor activates transcription by promoting chromatin remodeling at enhancers, primarily by recruiting the nucleosome remodeling factor (NURF) (Okada and Hirose 1998; Kwon et al. 2016) before associating with, or allowing, other proteins to modulate transcription including CBP (Philip et al. 2015; Boija et al. 2017). NURF can also directly interact with Ecdysone receptor (EcR; LOC406084) in order to potentially target different enhancer regions (Badenhorst et al. 2005). In our present study, EcR shows highly significant worker-specific chromatin and expression patterns (LogFC relative to queen = 0.24), suggesting that NURF-mediated transcriptional activation of enhancers could also be directed via this crucial developmental steroid hormone signaling pathway in honey bees. Mad/SMAD1 is also an enhancer-associated transcription factor that mediates the bone morphogenetic protein (BMP) signaling cascade, acting downstream from decapentaplegic (dpp) (Deignan et al. 2016). Mad/SMAD1 was demonstrated to interact directly with CBP (Pearson et al. 1999), colocalize with regions of H3K27ac at enhancers (Koenecke et al. 2016), and affect gene expression in a CBP-dependent manner (Waltzer and Bienz 1999). In our study, Magu (LOC411502; LogFC 0.12), BMP receptor 1B (LOC408442; LogFC 0.12), and Mad/SMAD1 (LogFC 0.19) show a significant worker caste bias in gene expression compared to queen and are all differentially DNA methylated genes in larvae (Foret et al. 2012), suggesting that BMP signaling via Mad-mediated enhancer activation may also play an important role in worker caste determination.

In contrast to worker, motifs for the transcriptional repressors brk and Hairy (LOC410468; HES1 in mammals) show the most significant enrichment in queen and accounted for 66% of 96hQ-specific H3K27ac regions. brk and Hairy were demonstrated to bind enhancers and mediate transcriptional repression (Campbell and Tomlinson 1999; Jaźwińska et al. 1999). Although Hairy can directly recruit the Sirt1 histone deacetylases to repress transcription (Rosenberg and Parkhurst 2002; Takata and Ishikawa 2003), both Hairy and brk associate with the corepressors Groucho (Gro; TLE in mammals) and C-terminal binding protein (CtBP) (Paroush et al. 1994; Poortinga et al. 1998; Hasson et al. 2001; Morel et al. 2001; Zhang et al. 2001; Barolo et al. 2002; Nagel et al. 2005). Groucho functions downstream from key signaling pathways such as Wg/Wnt and Dpp/TGF-beta and mediates deacetylation of histones through recruitment of HDAC1. In *A. mellifera* it is also a differentially DNA methylated gene (Foret et al. 2012). CtBP is an NAD(H)-regulated transcription factor that functions through the recruitment of a variety of histone modification enzymes or via the inhibition of CBP in order to repression transcription (for review, see Chinnadurai 2007), although there is evidence that CtBP can also activate transcription in certain contexts (Phippen et al. 2000; Fang et al. 2006). Significantly and in contrast to worker, in 96hQ-specific H3K27ac regions, transcription factor motifs are centered on peaks of H3K27ac, indicating the presence of a nucleosome and therefore potentially preventing accessibility and subsequent repressor function. This was equally true for Mad/SMAD1 motifs, which were also enriched in 27% of 96hQ-specific H3K27ac regions. In *Drosophila*, Mad and brk mediate opposing transcriptional effects in the BMP signaling pathway, in part by competing for binding to overlapping sites at certain enhancers during different development stages (Kirkpatrick et al. 2001). Previous studies demonstrated enrichment of Brinker sites at regions of H3K27ac in dorsal ectoderm enhancers during development and that they are occupied by Mad (Koenecke et al. 2016), therefore Mad may function in a similar way at queen intronic H3K27ac regions.

Taken together, we speculate that 96hW-specific intronic H3K27ac regions bear all the hallmarks of active enhancers. Furthermore, the majority of worker genes that are enriched for intronic H3K27ac are also transcription factors, suggesting further downstream gene expression cascades during worker caste development. 96hQ-specific regions could also be caste-specific enhancers but require further characterization. Therefore, we conclude that it is highly likely that H3K27ac and CBP play an important role at this key developmental stage in honey bee caste development through differential enhancer activation. This is further supported by elegant studies in the carpenter ant, *Camponotus floridanus*, where CBP-catalyzed H3K27ac was shown to be essential in establishing different worker castes and behaviors (Simola et al. 2013, 2016). We conclude that chromatin modifications play a crucial role in defining worker and queen honey bee castes by establishing and orchestrating caste-specific transcriptional networks; furthermore, it is the worker developmental pathway that is actively switched on from a default queen developmental program.

## Methods

### Age-matched larvae collection

To obtain worker larvae of known age, frames containing eggs and larvae were removed from healthy hives and placed as soon as possible in an incubator at 35°C and ∼80% humidity, in a warm room (at least 30°C). After removal from the incubator, frames were closely examined and an acetate sheet was laid over the patches of newly hatched larvae. The position of the sheet was marked on the edges of the frame to ensure accurate replacement at the time of collection. The locations of individual newly hatched larvae were marked on the sheet, and the frames were immediately returned to their original hives. The exact time was recorded. After 96 h, the frames were transferred back to a warm room and the larvae were collected with blunt nose soft forceps using the acetate sheet positioned over the frame according to the previously marked reference points. To obtain queen larvae of known age, standard queen raising techniques were used (Evans et al. 2013). Double grafting gave improved results and priming the queen cups with warm (35°C) royal jelly increased yield.

### Chromatin preparation

Chromatin was extracted from the larval heads (∼1.6 mm of the frontal end dissected in PBS) containing brain, optic and retinular ganglia, neurosecretory cells, glands (corpora allata, corpora cardiaca), suboesophageal ganglion, a small number of fat bodies, the maxillae, labium and mandibles, segmented imaginal antennae developing in hypodermal pockets, the openings of silk glands ducts at the tip of the labium-hypopharynx, trachea, and cuticle. The rest of the larval body is predominantly occupied by a large digestive system filled with processed food and bacteria (larvae do not defecate), a tracheal network and reproductive parts that at this stage of development are already large in queens and rudimentary in workers. Chromatin was cross-linked in fixation buffer (50 mM HEPES at pH 7.9, 1 mM EDTA at pH 8, 0.5 mM EGTA at pH 8, 100 mM NaCl, 1.8% formaldehyde) for 15 min. Reactions were quenched by washing twice in stop solution (125 mM glycine, 0.01% Triton X-100, PBS). Afterward, fixed larval heads were washed four times with wash buffer 1 (10 mM HEPES at pH 8, 10 mM EDTA at pH 8, 0.5 mM EGTA at pH 8, 0.25% Triton X-100), followed by four washes with wash buffer 2 (10 mM HEPES at pH 8, 1 mM EDTA at pH 8, 0.5 mM EGTA at pH 8, 0.01% Triton X-100, 200 mM NaCl). Larval heads were homogenized and centrifuged (1200*g*/10 min). All buffers from this step onward contained cOmplete protease inhibitor cocktail (Roche) and 2.5 mM sodium butyrate. Pellets were suspended in 5 mL lysis buffer 1 (2.5% glycerol, 50 mM Tris-HCl at pH 8.0, 140 mM NaCl, 0.5% IGEPAL, 0.25% Triton X-100, 1 mM EDTA) and incubated for 30 min on a rotator mixer at 4°C. Lysates were then centrifuged (1200*g*/10 min), and pellets were suspended in 5 mL lysis buffer 2 (10 mM Tris-HCl at pH 8.0, 200 mM NaCl, 1 mM EDTA) and incubated for another 30 min on a rotator mixer at 4°C. Lysates were centrifuged (1200*g*/10 min), and pellets were suspended in 900 µL sonication buffer (10 mM Tris-HCl at pH 8.0, 0.5% SDS, 1 mM EDTA). Chromatin was sonicated using a Bioruptor (3 × 15 min cycles [30 sec on, 30 sec off] at high power). Lysates were cleared by centrifugation (20,000*g*/10 min), and sonication was checked by agarose gel electrophoresis.

### Chromatin immunoprecipitation and ChIP-seq library preparation

Chromatin was concentrated using a Millipore Amicon concentrator (3 kDa cutoff). Fifty microliters chromatin aliquots was diluted 10-fold with ChIP dilution buffer (0.01% SDS, 1.1% Triton X-100, 1.2 mM EDTA, 16.7 mM Tris-HCl at pH 8.0, 167 mM NaCl). Antibodies (H3K4me3 [Active Motif, 39159]; H3K27ac [Active Motif, 39133], and H3K36me3 [Abcam, ab9050]) were added according to the manufacturers’ instructions, and samples were incubated overnight on a rotator mixer at 4°C. Thirty microliters of magnetic protein A Dynabeads (Invitrogen) was added to each reaction, and samples were incubated for 4 h on a rotator mixer at 4°C. Beads were washed twice with 500 µL wash buffer A (50 mM Tris-HCl at pH 8.0, 150 mM NaCl, 1 mM EDTA, 0.1% SDS, 1% NP-40, 0.5% sodium deoxycholate) and once with 500 µL wash buffer B (50 mM Tris-HCl at pH 8.0, 500 mM NaCl, 1 mM EDTA, 0.1% SDS, 1% NP-40, 0.5% sodium deoxycholate) and wash buffer C (50 mM Tris-HCl at pH 8.0, 250 mM LiCl, 1 mM EDTA, 1% NP-40, 0.5% sodium deoxycholate). After these washes, DNA was eluted from the beads by incubation in 200 µL elution buffer (1% SDS, 100 mM NaHCO_3_) for 40 min at 65°C in a ThermoMixer. Fifty micrograms of RNase A was added, and samples were incubated for 15 min at 37°C. NaCl was then added to a final concentration of 500 mM. Afterward, the samples were incubated overnight with 40 µg of proteinase K at 65°C in a ThermoMixer. DNA was purified with GeneJET PCR Purification columns (Thermo Fisher Scientific). NEBNext Ultra II DNA Library Prep Kit for Illumina (NEB) was used to make sequencing libraries from 0.5 to 1 ng of DNA following the manufacturer's instructions. Seventy-five base-pair SE next-generation DNA sequencing was carried out by the Barts and The London Genome Centre at Queen Mary University of London on the Illumina NextSeq 500 platform.

### RNA isolation and RNA-seq library preparation

Larval heads were dissected and individually snap frozen in liquid nitrogen. Total RNA from individual heads was isolated using the TRIzol method, followed by the use of RNA Clean-up and Concentration kit (Zymo Research). mRNA was isolated with poly(A) mRNA Magnetic Isolation Module (NEB) from 1 µg total RNA. RNA-seq libraries were constructed using the NEBNext Ultra Directional RNA Library Prep Kit for Illumina (NEB) following the manufacturer's instructions. One hundred base-pair PE next-generation sequencing was carried out by the Barts and The London Genome Centre at Queen Mary University of London on the Illumina NextSeq 500 platform.

### ChIP-seq analysis

The genome assembly Amel_4.5 (GCF_000002195.4) was downloaded from the NCBI and indexed using Bowtie 2 (v2.2.8) (Langmead and Salzberg 2012). ChIP-seq samples were mapped to this indexed genome using Bowtie 2 with default parameters. Detailed mapping statistics for each sample is available in Supplemental Table S7. Reads were counted into windows of width 100 bp with a spacing of 50 bp (each window therefore overlaps two other windows) using csaw (Lun and Smyth 2015). Duplicate reads were included in the analysis, mapping quality was restricted to ≥20, and each read was extended to 250 bp. Input reads were counted using the same parameters except each region was expanded to 5000 bp (±2450 bp). Windows that could not be expanded (e.g., those at the end of contigs) were removed from analysis. The count value of each window (*C*_*i*_) for each sample, *i*, was then normalized to the background counts (*C*_*b*_) using the following equation:

(1) $$NC_{i}=\frac{C_{i}N_{b}}{C_{b}N_{i}}$$

where *N*_*i*_ is the total number of reads sequenced for each sample, *i*, and *N*_*b*_ is the total number of reads sequenced in the appropriate input sample. To determine differential windows, we computed moderated *t*-statistics using empirical Bayes moderation of the standard errors with the *limma* R package (Ritchie et al. 2015).

### RNA-seq analysis

The cDNA of reference transcripts and ncRNA were downloaded from Ensembl Metazoa (https://metazoa.ensembl.org/index.html) in FASTA format using genome version GCA_000002195.1. These two FASTA files were concatenated and supercontigs were removed using linux command grep with the following string: “supercontig|$genome_version:[^1-9XMY]”. Kallisto (Bray et al. 2016) was used to build an index for further mapping using default parameters. Each sample's FASTQ file was mapped using Kallisto quant with default parameters except for increasing the number of bootstrap samples to 100 and setting the strand-specific nature of the reads using parameters “-b 100 --rf-stranded”. Detailed mapping statistics for each sample is available in Supplemental Table S8. To determine differential expression, the resulting files from the mapping were used with the R program sleuth (Pimentel et al. 2017). Default parameters were used throughout analysis. Sleuth uses a likelihood ratio test, and hence we tested for those genes whose abundance is significantly better explained when caste is included in the model compared to a reduced model in which a single parameter is fitted for each gene.

### Gene Ontology analysis

*Drosophila melanogaster* GO terms were downloaded from FlyBase [http://flybase.org]. Each *D. melanogaster* gene was mapped to its *A. mellifera* ortholog using HymenopteraMine (http://hymenopteragenome.org/hymenopteramine/begin.do). GO analysis was performed using the R package, topGO (http://bioconductor.org/packages/release/bioc/html/topGO.html).

### Transcription factor motif analysis

Transcription factor motif analysis was performed using CentriMo (Bailey and MacHanick 2012), which is part of the MEME suite tools. Individual peaks of H3K27ac were extended to 5 kbp from the center of the called peak.

## Data access

ChIP-seq and RNA-seq data from this study have been submitted to the NCBI Gene Expression Omnibus (GEO; https://www.ncbi.nlm.nih.gov/geo/) under accession number GSE110642.

## Supplementary Material

Supplemental Material
