# Supplementary material for: Phenotypically distinct female castes in honey bees are defined by alternative chromatin states during larval development
Source: Genome Res. 2018 Oct;28(10):1532–42. doi: 10.1101/gr.236497.118 (PMC6169885; doi:10.1101/gr.236497.118)
Supplement: Supplemental Material [file supp_28_10_1532__index.html]

Phenotypically distinct female castes in honey bees are defined by alternative chromatin states during larval development — Phenotypically distinct female castes in honey bees are defined by alternative chromatin states during larval development — Supplemental Material 

# Phenotypically distinct female castes in honey bees are defined by alternative chromatin states during larval development

## Supplemental Material

- Supplemental\_Material.docx
