# Supplementary material for: Phenotypically distinct female castes in honey bees are defined by alternative chromatin states during larval development
Source: Genome Res. 2018 Oct;28(10):1532–42. doi: 10.1101/gr.236497.118 (PMC6169885; doi:10.1101/gr.236497.118)
Supplement: Supplemental Material [file supp_gr.236497.118_Supplemental_Material.docx]

**Supplemental Materials**

**Phenotypically distinct female castes in honey bees are defined by alternative chromatin states during larval development.**

**Marek Wojciechowski,^1,4^ Robert Lowe,^2,4^ Joanna Maleszka,^3^ Danyal Conn,^1^ Ryszard Maleszka,^3,5^ and Paul J. Hurd^1,5^**

^1^ School of Biological and Chemical Sciences, Queen Mary University of London, Mile End Road, London, E1 4NS, UK.

^2^ The Blizard Institute, Barts and The London School of Medicine and Dentistry, Queen Mary University of London, 4 Newark Street, London, E1 2AT, UK.

^3^ Research School of Biology, Australian National University, ACT, 0200, Australia.

^4^ These authors contributed equally to this work.

^5^ Corresponding authors

E-mail: p.j.hurd@qmul.ac.uk

E-mail: ryszard.maleszka@anu.edu.au

**Table of contents**

**Supplemental Figures S1 – S13**

**Supplemental Tables S1- S8**

**Supplemental Figure S1.** Scatter plot of the enrichment values for each of the histone modifications profiled for the two biological replicates (R1, R2).


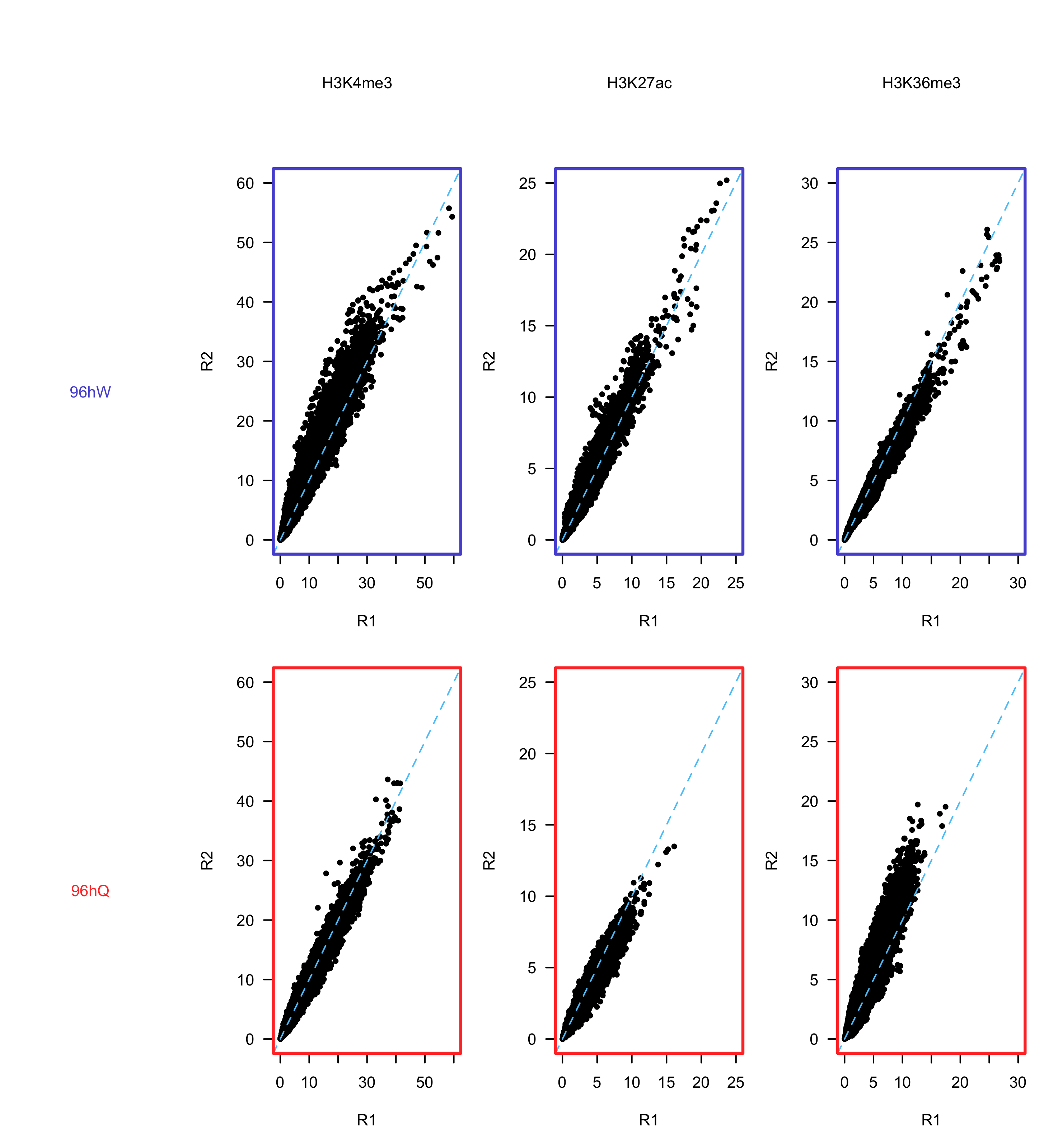


**Supplemental Figure S2**. Bar plots showing the percentage of H3K4me3, H3K27ac and H3K36me3 peaks that have a > 3-fold enrichment over input and the distance from the nearest TSS. This revealed that 82% (96hW) and 86% (96hQ) of all H3K4me3 peaks, 61% (96hW) and 58% (96hQ) of all H3K27ac peaks and 30% (96hW) and 22% (96hQ) of all H3K36me3 peaks are located within +/- 2kbp of the TSS of a gene.

**Supplemental Figure S3**. Bar plots showing the number of genes in common between the top five GO terms presented in Fig. 2B. For the 96hW GO analysis (left panel), there are a mean number of 53.6 genes in the top five terms. As expected, there is considerable overlap of genes across these top five GO terms. In total there are 70 unique genes identified in the top five GO terms. For the 96hQ GO analysis (right panel), there are a mean number of 391.6 genes in the top five GO terms. Again, as expected there is considerable overlap among GO terms with the majority of genes present in all five.

**Supplemental Figure S4**. A scatter plot showing the logFC(96hQ/96hW) of differential H3K36me3 enrichment levels against the change in fraction of overlapping DNA methylation differences. No statistically significant correlation (Spearman’s rank correlation p-value = 0.3495) or association (chisq-test p-value = 0.3424) was found.

**Supplemental Figure S5**. A bar plot showing the number of DMPs and their location to the nearest intron/exon boundary. In black are those DMPs which overlap with H3K36me3 peaks and in grey are randomly selected DMPs (background). DMPs that overlap H3K36me3 and DMPs that do not, show strong enrichment for intron/exon boundaries.

**Supplemental Figure S6.** A multidimensional scaling plot of the gene expression values as measured by RNA-seq for four 96hQ larval heads and four 96hW larval heads.

**Supplemental Figure S7.** The negative log p-value for the top five biological process GO terms for those genes which show increased expression in 96hW compared to 96hQ (left panel) and for those which show an increased expression in 96hQ compared to 96hW (right panel).

**
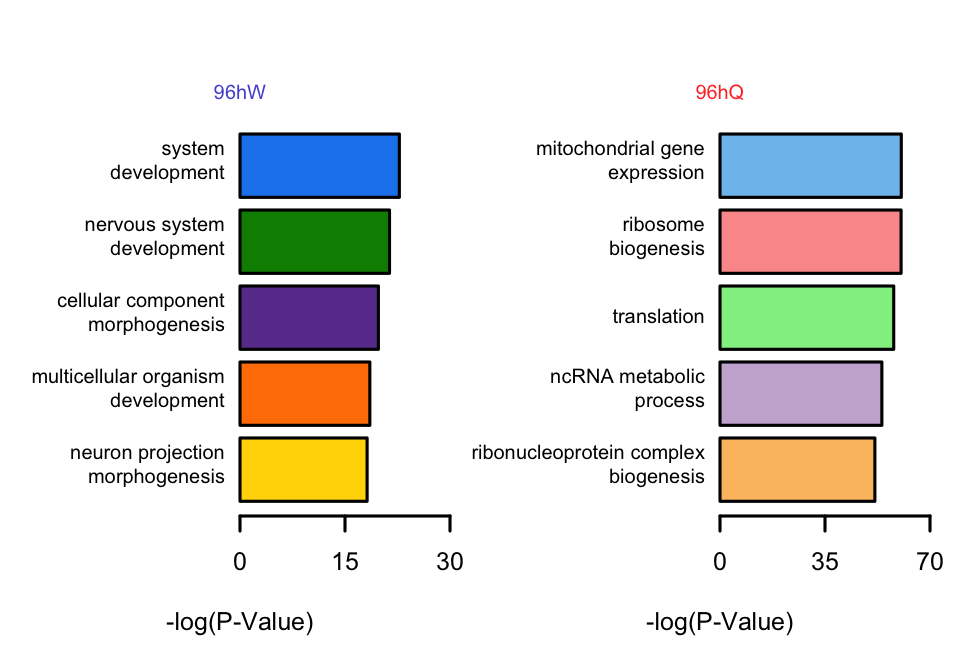
**

**Supplemental Figure S8.** Scatter plot of LogFC in expression between 96hW and 96hQ against the LogFC in 96hQ and 96hW from Ashby et al. 2016. A highly significant correlation ($\rho=0.51$; p-value = 5.3 $\times$ 10^-72^) is observed between the two datasets.

**
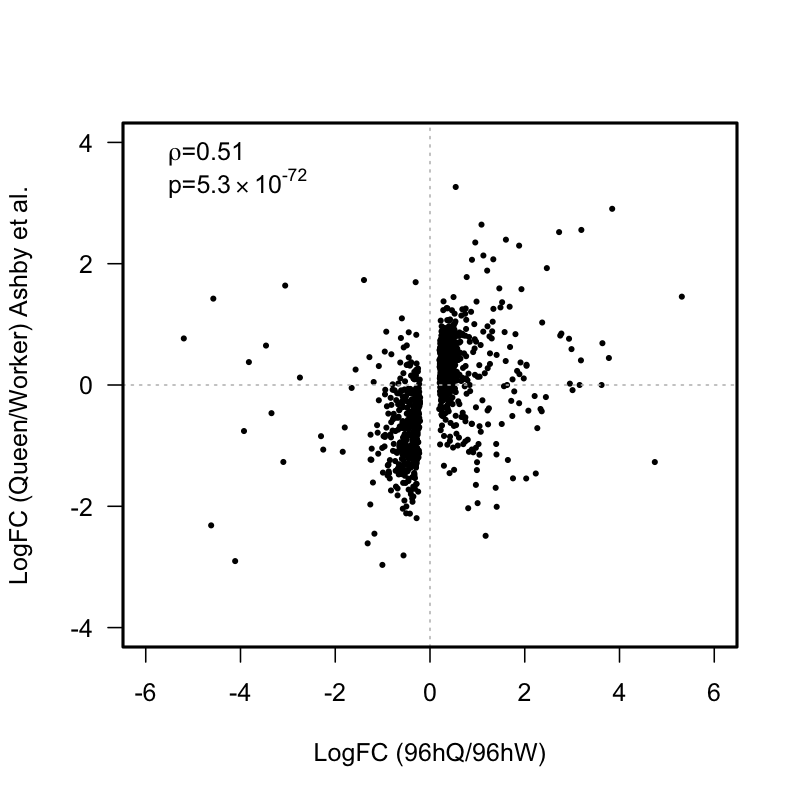
**

**Supplemental Figure S9.** Bar plots showing the number of genes in common between the top five GO terms presented in Fig. 2E. For the 96hW GO analysis (left panel), there are a mean number of 62.6 genes per GO term. As expected, there is considerable overlap of genes across these top five GO terms. For the 96hQ GO analysis (right panel), there are a mean number of 31 genes per GO term. Again, as expected there is considerable overlap among GO terms with the majority of genes present in all five.

**Supplemental Figure S10.** A screenshot from the genome browser display showing the read alignments over pyruvate kinase (*PYK*; 552007) without any processing.


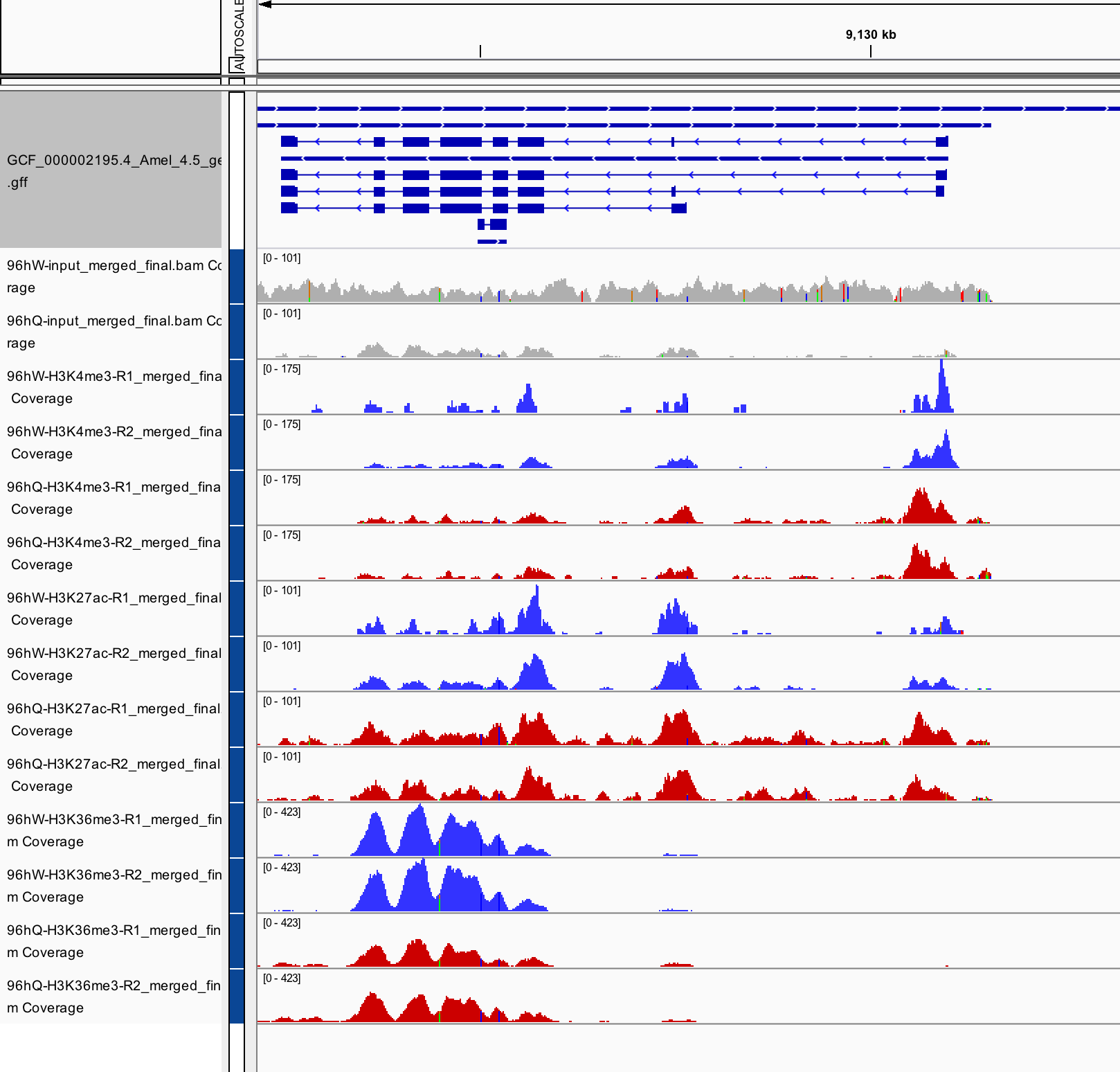


**Supplemental Figure S11.** A boxplot of the LogFC in expression (96hQ/96hW) for those genes containing 96hW-specific intronic H3K27ac and 96hQ-specific intronic H3K27ac.

**
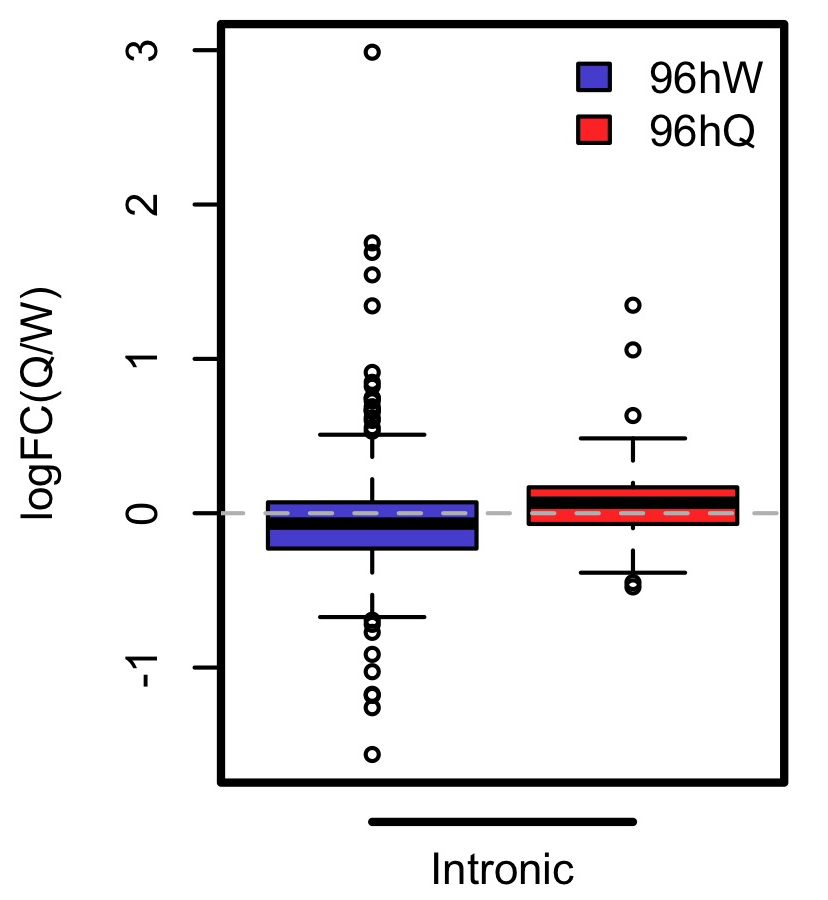
**

**Supplemental Figure S12**. A line plot showing the number of genes at varying distances from peaks of H3K27ac that show a > 3-fold enrichment over input. The number of genes were calculated by counting genes in bins of 10kbp.

**Supplemental Figure S13**. A line plot showing the average Log(expression) of the genes in each 10kbp bin defined in Supplemental Figure S12**.** A peak in expression is seen in both 96hQ and 96hW at a distance of 130-140kbp.

**Supplemental Table S1.** List of GO data for 96hQ ChIP-seq enrichment differences

| GO.ID | Term | Gene IDs | classicFisher |
| --- | --- | --- | --- |
| GO:0043603 | cellular amide metabolic process | 725062;552106;552649;100576457;412549;552118;726874;552152;552062;552025;552056;409589;725357;100578551;552768;408515;551613;552272;726899;409552;100576960;552774;725147;725197;725136;413868;552790;550687;724790;551418;552266;409832;406099;724162;551330;409866;411380;550715;408336;726171;411646;551107;552736;552726;412521;409290;550711;413711;724816;413877;725039;726766;724475;727436;552524;725555;409280;725012;552247;100577676;726326;551419;100578030;552287;412536;552710;409109;552314;726823;725942 | 1.30E-06 |
| GO:0002181 | cytoplasmic translation | 552106;100576457;409589;408515;552272;409552;552774;725147;413868;551418;552266;409832;406099;551330;411380;550715;726171;551107;552726;409290;550711;727436 | 4.20E-06 |
| GO:0006412 | translation | 725062;552106;552649;100576457;412549;726874;552152;552062;552025;552056;409589;725357;100578551;552768;408515;551613;552272;409552;100576960;552774;725147;725197;725136;413868;724790;551418;552266;409832;406099;724162;551330;409866;411380;550715;408336;726171;411646;551107;552726;412521;409290;550711;413711;413877;725039;726766;724475;727436;725555;409280;725012;726326;552287 | 7.50E-06 |
| GO:0006518 | peptide metabolic process | 725062;552106;552649;100576457;412549;552118;726874;552152;552062;552025;552056;409589;725357;100578551;552768;408515;551613;552272;726899;409552;100576960;552774;725147;725197;725136;413868;724790;551418;552266;409832;406099;724162;551330;409866;411380;550715;408336;726171;411646;551107;552726;412521;409290;550711;413711;724816;413877;725039;726766;724475;727436;725555;409280;725012;552247;100577676;726326;100578030;552287;412536;409109;552314;726823;725942 | 1.40E-05 |
| GO:0043604 | amide biosynthetic process | 725062;552106;552649;100576457;412549;726874;552152;552062;552025;552056;409589;725357;100578551;552768;408515;551613;552272;726899;409552;100576960;552774;725147;725197;725136;413868;552790;724790;551418;552266;409832;406099;724162;551330;409866;411380;550715;408336;726171;411646;551107;552726;412521;409290;550711;413711;413877;725039;726766;724475;727436;725555;409280;725012;552247;100577676;726326;552287;552710;409109 | 3.70E-05 |
| GO:0042254 | ribosome biogenesis | 726812;100576457;552280;724203;409589;551311;411603;408515;725947;409552;413868;413720;724162;550715;726171;552726;409290;550711;725441;413758;100576775;724538;551343;726279;552510;724794;412830;551413 | 3.90E-05 |
| GO:1901566 | organonitrogen compound biosynthetic pro... | 725062;552106;552649;100576457;412549;552118;726874;552152;552062;552025;411719;552056;409589;725357;100578551;552768;411525;408515;551613;552272;727483;726222;726899;409552;100576960;552774;725147;725197;725136;552699;413868;552790;724790;551418;552266;409832;406099;724162;551330;409866;411380;550715;408336;726171;411646;551107;552084;413078;550703;552726;551662;412234;412521;409290;550711;413711;551341;413877;725039;726766;724475;727436;412675;410066;727520;725555;409280;725012;552364;552247;100577676;726326;552287;727630;552710;409109;726823;412670;551857;551696;551849 | 5.20E-05 |
| GO:0043043 | peptide biosynthetic process | 725062;552106;552649;100576457;412549;726874;552152;552062;552025;552056;409589;725357;100578551;552768;408515;551613;552272;726899;409552;100576960;552774;725147;725197;725136;413868;724790;551418;552266;409832;406099;724162;551330;409866;411380;550715;408336;726171;411646;551107;552726;412521;409290;550711;413711;413877;725039;726766;724475;727436;725555;409280;725012;552247;100577676;726326;552287;409109 | 6.20E-05 |
| GO:0022613 | ribonucleoprotein complex biogenesis | 726812;100576457;552062;552280;724203;409589;551311;411603;408515;725947;409552;413868;413720;724162;550715;552260;726171;552726;412521;551331;409290;550711;552559;725441;726766;727436;413758;100576775;724538;551343;726279;552510;724794;412830;551413 | 9.30E-05 |
| GO:0008612 | peptidyl-lysine modification to peptidyl... | 410763;100578266;413869 | 0.00038 |
| GO:1901564 | organonitrogen compound metabolic proces... | 725062;412328;552106;552649;100576457;412549;552118;726874;552152;552062;552025;411719;552056;409589;725357;100578551;552768;411525;408515;551613;552272;727483;726222;726899;409552;100576960;552774;551866;725147;725197;725136;552699;413868;552790;550687;724790;551418;552266;409832;406099;724162;552023;551330;409866;411380;550715;552007;408336;726171;411646;551107;552736;552084;413078;550703;552726;551662;412234;412521;551660;409290;550711;413711;551341;724816;413877;725039;552273;726766;724475;727436;412675;410066;412094;552524;727520;725555;409280;725012;552364;725044;551492;552247;100577676;550724;726326;551419;100578030;552287;412536;727630;552710;409109;552314;726823;412670;551857;725325;551768;551696;551849;725942 | 0.00071 |
| GO:0000027 | ribosomal large subunit assembly | 100576457;724203;409589;551311;550715;726171 | 0.00121 |
| GO:0042273 | ribosomal large subunit biogenesis | 100576457;552280;724203;409589;551311;408515;413868;550715;726171;409290;551343 | 0.00139 |
| GO:0043524 | negative regulation of neuron apoptotic ... | 551330;409290;550978;409414;552524;727022 | 0.00224 |
| GO:0034058 | endosomal vesicle fusion | 412816;100577764;727031 | 0.00337 |
| GO:0048227 | plasma membrane to endosome transport | 412816;100577764;727031 | 0.00337 |
| GO:0034622 | cellular macromolecular complex assembly | 100576457;552062;727599;724203;409589;551311;725947;409552;552253;726900;409439;550715;552260;726171;727473;725680;412521;551331;413845;550824;552559;411410;411535;724816;725789;551784;412119;726766;727436;551343;410493;552038;551492;727031;726326;412823;727630;411886;727192;409581;724128;551918;727153 | 0.00414 |
| GO:0034660 | ncRNA metabolic process | 726812;726874;552025;411719;551311;411603;552768;551613;725947;413250;552662;100576960;725136;413868;413720;724162;408336;413877;725441;551809;726766;100577689;413758;724538;551343;726279;552510;551355;724794;412830;551413 | 0.00519 |
| GO:0006695 | cholesterol biosynthetic process | 552163;725018 | 0.00522 |
| GO:0060568 | regulation of peptide hormone processing | 100578030;412536 | 0.00522 |
| GO:0060570 | negative regulation of peptide hormone p... | 100578030;412536 | 0.00522 |
| GO:0090110 | cargo loading into COPII-coated vesicle | 409439;411410 | 0.00522 |
| GO:0065003 | macromolecular complex assembly | 100576457;552062;727599;724203;409589;412355;551311;725947;409552;552253;726900;409866;409439;550715;552260;726171;727473;725680;412521;551331;413845;550824;552559;411410;411535;724816;550886;725789;551784;412119;726766;727436;551343;410493;552038;551492;727031;726326;412823;727630;411886;551109;727192;409581;724128;551918;727153 | 0.00585 |
| GO:0000290 | deadenylation-dependent decapping of nuc... | 552260;552284;412521 | 0.00637 |
| GO:0032509 | endosome transport via multivesicular bo... | 727354;727141;100577764;551408;411857;727142 | 0.00751 |
| GO:0042255 | ribosome assembly | 100576457;724203;409589;551311;409552;550715;726171 | 0.00812 |
| GO:0016072 | rRNA metabolic process | 726812;551311;411603;725947;413868;413720;724162;725441;100577689;413758;724538;551343;726279;552510;724794;412830;551413 | 0.0082 |
| GO:0007041 | lysosomal transport | 725680;410493;552555;412346;411886;727153 | 0.00918 |
| GO:0097352 | autophagosome maturation | 100577764;410493;552038;552555;727630;411886;727153 | 0.0096 |
| GO:0097576 | vacuole fusion | 100577764;410493;552038;552555;727630;411886;727153 | 0.0096 |

**Supplemental Table S2.** List of GO data for 96hW ChIP-seq enrichment differences.

| **GO.ID** | **Term** | **Gene IDs** | **classicFisher** |
| --- | --- | --- | --- |
| GO:0009653 | anatomical structure morphogenesis | 411276;552187;724704;552782;409643;411569;409611;725928;408894;413558;410447;413242;410689;411158;410686;408661;411890;409856;410723;408438;410632;410956;410280;413968;726842;724607;725943;409001;408792;724595;413569;413683;408328;408763;409543;551733;413125;551746;411177;410685;413377;552485;410351;408777;727000;408810;409890;727235;409390;409707;411011;410368;100578826;551130;410279;550804;411575;408670;408786;408869;408890;410739;552617;552340;724991;408298;408936;409902;410606;410975;412739;411250;412108;408264;408276;551765;726469;410913;413215;408591;413677;725190;411981;409362;410674;724929;408278;724207;411112;412256;409084;406084;410497;410785;409501;726709;409348;413388;408900;412046;551826;411700;411048;409717;408976;410009;409024;727312;408534;411539;406106;725679;408783;412602;413440;100578885;413246;410208;409983;408703;551602;406111;408616;408896;413289;412406;408427;410340;550818;726683;409049;410906;725311;551291;100578927;550966;410413;552235;725237;727081;725436;724338;406154;726280;410933;724501;727335;411553;551848;413310;413618;410758;409470;409321;406122;725189;408939;410585;552397;411843;408325;410840;412175;409576;410427;408697;410253;412274;409006;724516;409961;412468;408721;724344;724651;408951;410505;551735;724603;412770;413474;411812;727528;410235;409151;410923;412601;411175;410609;408372;409227;409861;408563;726510;410809;411157;409780;411566;100577697;550645;100578883;406086;411116;411083;408470;410256;413371;100576321;410989;412742;552099;724322;408311;551124;725591;413385;724311;411288;413467;410203;551349;411678;724375;408725;100578984;552709;724518;100577393;724292;410776;410741;724851;408782;726349;726308;725391;411820;551706;408844;724517;725433;410190;408277;410348;408519;551250;411009;410904;410918;410553;100576392;409722;410573;408358;551170;410951;551259;552030;410326;408333;412243;409898;408793;724172;725923;410998;408423;724138;410399;552545;412528;551514;410195;100577365;413423;411917;411744;408959;100578439;551928;413197;408306;409836;409904;725091;408551;726681;411086;551272;552589;406123;724613;411124;724159;412763;410922;726582;408552;409942;413052;411238;408405;411827;408602;408359;408923;413054;410853;409778;410204;410271;552079;408821;100577801;411038;550930;408444;725782;408399;414025;408677;410222;410649;408583;726790;410211;727399;410159;725294;408857;551895;100578515;411079;725776;726914;100578467;100578865;408292;408343;408365;408411;408429;408494;408508;408664;408758;408779;408862;409051;409658;409697;409733;409921;410228;410902;412840;412968;413410;552265;552804;726302 | < 1e-30 |
| GO:0048731 | system development | 411276;552187;552782;412801;409643;100576129;411569;409611;725928;408894;413558;408509;413242;726419;410689;411158;408726;100578818;410686;726461;408661;411890;413987;409856;410723;408438;410632;410956;410280;413968;726842;724607;725943;409001;408792;724595;413569;413683;408763;409543;551733;413125;551071;551746;411177;410685;413377;410730;410351;408777;727000;408810;409890;727235;409390;409707;411011;100578826;551130;410279;408835;550804;725754;410258;408670;408786;408869;100576251;408890;552094;410739;552617;552340;724991;408298;408936;409902;410606;413288;410975;412739;411250;412108;408264;724973;408276;551765;726469;552377;410913;413215;413677;725190;408430;725551;408431;409165;411981;409362;410674;724929;408278;724207;411112;412256;409084;724902;406084;410497;410785;409501;409893;413130;726709;409348;413388;408900;412046;412884;551826;408852;411700;411048;409717;408976;410009;727312;408534;411539;406106;725679;408783;412602;413440;100578885;413246;410208;409983;408703;408406;551602;408616;408896;413289;412406;408427;550818;725038;726683;409049;725311;551291;551425;100578927;550966;410413;552235;725237;408489;725436;406154;726280;724501;727335;411553;725155;551848;410174;413310;413618;410758;408631;409470;410514;409321;406122;725189;410585;410269;552397;408325;410840;409576;410427;408697;410253;412274;409006;724516;409961;726587;408721;724344;724651;408951;410505;551735;724603;412770;413474;411812;727528;410235;409151;410923;412601;411175;410609;408372;409227;409861;726350;408563;726510;410809;411157;411566;100577697;100578883;410732;406086;411116;411083;408470;410256;413371;410989;412742;552099;724322;408311;551124;725591;413385;724311;411288;413467;410203;551349;411678;724375;551894;408725;410767;413755;100578984;552709;724518;100577393;724292;410776;410741;100578976;724851;408782;726349;726308;725391;725474;411820;411144;551706;408844;724517;725433;410190;408277;551250;411009;410904;410918;410553;100576392;409142;409722;410573;408358;551170;410951;551259;552030;551102;411490;410326;408333;412243;409898;408793;724172;725923;410998;724138;410399;552545;412528;551514;100577365;413423;411917;411744;408959;551928;413197;408306;409836;409904;409928;725091;408551;411086;551272;552589;410425;100576700;406123;724613;411124;410487;724159;412763;410922;726582;408552;409942;410502;410920;411238;408405;411827;408602;725893;408359;408923;410853;409778;410271;552079;408821;100577801;411038;408383;550930;408399;414025;408677;410222;410649;408981;408583;726790;410211;727399;551356;410159;725294;408857;551895;100578515;411079;725776;726914;100578467;100578865;408292;408343;408365;408411;408429;408494;408508;408664;408758;408779;408862;409051;409658;409697;409921;410228;410278;410902;412840;412968;413410;551360;552265;724997;725932;726302;726729 | < 1e-30 |
| GO:0048513 | animal organ development | 411276;412801;100576129;411569;409611;725928;413558;413242;410689;100578818;408661;409856;410723;410632;410956;413968;726842;724607;409001;408792;724595;413569;413683;408763;409543;551733;413125;551746;411177;410685;413377;410351;408810;409890;727235;409390;411011;100578826;551130;410279;725754;410258;408670;408786;408869;100576251;552094;410739;552340;724991;408936;409902;413288;412739;411250;412108;408264;408276;551765;726469;552377;410913;413215;413677;725190;725551;408431;411981;724929;408278;412256;409084;724902;406084;410497;409501;413130;726709;409348;413388;412046;412884;408852;411700;411048;408976;410009;727312;411539;406106;725679;408783;412602;413440;100578885;413246;410208;409983;408703;551602;408616;413289;412406;550818;726683;409049;725311;551425;550966;410413;552235;725237;408489;406154;726280;724501;411553;725155;551848;410174;410758;409470;409321;406122;725189;410585;410269;552397;410840;409576;410427;410253;409006;724516;409961;726587;408721;724651;408951;410505;551735;724603;412770;413474;411812;727528;410235;409151;410923;412601;410609;408372;409227;726350;726510;410809;411157;411566;100577697;100578883;406086;411083;410256;413371;410989;412742;552099;724322;408311;551124;725591;413385;724311;410203;551349;411678;100578984;552709;100577393;724292;410776;410741;724851;408782;725391;725474;411820;411144;551706;408844;724517;410190;408277;411009;410904;410918;410553;100576392;409722;408358;551170;410951;551259;552030;551102;411490;410326;408333;412243;409898;408793;724172;725923;410998;410399;552545;412528;551514;100577365;413423;551928;413197;408306;409836;409928;725091;408551;411086;551272;552589;100576700;406123;724613;411124;410487;724159;412763;410922;408552;409942;410502;410920;411238;408405;411827;408602;725893;408923;410853;410271;552079;100577801;411038;408383;550930;408399;414025;408677;410649;408583;726790;410211;727399;551356;410159;551895;100578515;411079;725776;100578467;100578865;408292;408365;408411;408429;408494;408508;408664;408758;408779;408862;409658;409697;409921;410228;410278;410902;412840;413410;551360;726302;726729 | 5.60E-30 |
| GO:0048856 | anatomical structure development | 411276;552187;724704;552782;412801;409643;100576129;411569;409611;725928;408894;413558;410447;408509;413242;726419;410689;411158;408726;100578818;410686;726461;408661;409708;411890;413987;409856;410723;408438;410632;410956;410280;413968;726842;724607;725943;409001;408792;412878;724595;413569;727238;413683;408328;408763;409543;551733;413125;551071;551746;411177;410685;413377;552485;725195;100577280;410730;410351;408777;727000;408810;409890;727235;409390;409707;411011;410368;100578826;551130;410279;408835;550804;411575;725754;408986;410258;408670;408786;408869;100576251;408890;552094;410739;552617;411969;552340;724991;408298;408936;409902;410606;413288;410975;412739;411250;412108;408264;724973;408276;408769;551765;726469;552377;410913;413215;408591;413677;725190;408430;725551;408431;409165;411981;409362;410674;724929;408278;724207;411112;412256;409084;724902;406084;410497;410785;409501;409893;413130;726709;409348;413388;408900;412046;412884;551826;408852;411700;411048;409717;408976;410009;409405;409024;727312;408534;411539;406106;725679;408783;412602;413440;100578885;413246;410208;409983;408703;408406;410178;551602;406111;408616;409628;408896;413289;412406;408427;410340;550818;725038;726683;409049;411227;410906;725311;551291;551425;100578927;550966;410413;552235;725237;408489;727081;725436;724338;406154;726280;410933;724501;727335;411553;725155;411115;551848;412846;410174;413310;413618;410758;408631;409470;410514;409321;406122;725189;408939;410585;409854;410269;100576586;552397;411843;408325;410840;412175;409576;410427;408697;410253;412274;409006;724516;409961;412468;726587;408721;724344;724651;408951;410505;551735;724603;412770;413474;411812;727528;410235;409151;410923;410554;412601;411175;410609;408372;409227;409861;726350;408563;726510;410809;411157;409780;411566;100577697;550645;100578883;410732;406086;411116;411083;408470;410256;413371;100576321;410989;412742;552099;724322;408311;551124;725591;413385;724311;411288;413467;410203;551349;411678;724375;551894;408725;410767;413755;100578984;552709;409727;724518;100577393;724292;410776;410741;100578976;724851;408782;726349;726308;725391;725474;408723;411820;411144;551706;408844;724517;725433;410190;408277;410348;408519;551250;411009;410904;410918;408658;410553;100576392;409142;409722;410573;408358;551170;410951;551259;552030;551102;726375;411490;410326;408333;412243;409898;408793;724172;725923;410998;408423;724138;410399;552545;412528;551514;410195;100577365;413423;411917;411744;408959;100578439;551928;413197;408306;409836;409904;409928;725091;408551;726681;411086;408891;551272;552589;410425;100576700;406123;724613;411124;410487;724159;412763;410922;726582;408552;409942;413052;410502;410920;411238;408405;411827;408602;725893;408359;408923;413054;410853;409778;410204;410271;552079;408821;100577801;411038;408383;410371;550930;408444;725782;408399;414025;408677;410222;410649;408981;408583;408434;726790;410211;727399;551356;410159;725294;408857;551895;100578515;411079;725776;726914;100578467;100578865;408292;408343;408365;408411;408429;408494;408508;408664;408758;408779;408862;409051;409658;409697;409733;409921;410228;410278;410902;412840;412968;413410;551360;552265;552804;724997;725932;726302;726729 | 3.30E-29 |
| GO:0032502 | developmental process | 411276;552187;724704;411534;410925;552782;412801;409643;100576129;411569;409611;725928;408894;413558;410447;408509;413242;726419;410689;411158;408726;100577778;100578818;410769;410686;726461;408661;409708;411890;413987;409856;410723;408438;409305;410632;410956;410280;413968;552747;726842;552166;724607;725943;409001;408792;412878;724595;413569;727238;413683;408328;408763;409543;551733;413125;724460;551071;551746;411177;410685;413377;552485;725195;100577280;410730;410351;408777;727000;408810;409890;727235;409390;409707;411011;410368;100578826;551130;410279;408835;550804;411575;725754;408986;410258;408670;408786;408869;100576251;408890;552094;410739;552617;411969;552340;724991;408298;408936;409902;410606;413288;410975;412739;411250;412108;408264;724973;408276;408769;551765;726469;552377;410913;413215;408591;413677;408915;725190;408430;725551;408431;409165;411981;409362;410674;724929;408278;724207;411112;412256;409084;724902;406084;410497;410785;409501;409893;413130;726709;409348;408348;413388;408900;412046;412884;551826;408852;411700;411048;409717;408976;410009;409405;726018;409024;409965;727312;408534;411539;406106;725679;408783;412602;413440;100578885;413246;410208;409983;408703;408406;410178;551602;406111;408616;409628;408896;413289;410352;412406;408427;410340;550818;725038;726683;409049;411227;410906;725311;551291;551425;100578927;550966;410413;552235;725237;408489;727081;725436;724338;406154;726280;410933;724501;727335;411553;725155;411115;551848;412846;410174;413310;413618;410758;408631;409470;410514;409321;406122;725189;408939;410585;409854;410269;100576586;552397;411843;408325;410840;412175;409576;410427;408697;410253;412274;409006;724516;409961;412468;726587;408721;724344;724651;408951;410505;551735;724603;412770;413474;411812;408577;727528;410235;409151;410923;410554;412601;411175;410609;408372;409227;552078;411754;409861;726350;408563;726510;410809;411157;409780;411566;100577697;550645;100578883;410732;406086;411116;411083;408470;410256;413371;100576321;410989;412742;552099;724322;408311;551124;725591;413385;724311;411288;413467;410203;551349;411678;724375;551894;408725;410767;413755;100578984;552709;409727;724518;100577393;724292;410776;410741;100578976;724851;408782;726349;726308;725391;725474;408723;411820;411144;551706;408844;724517;725433;410190;408277;410348;408519;551250;411009;410904;410918;408658;410553;100576392;409142;409722;410573;408358;551170;410951;551259;552030;551102;726375;411490;410326;408333;412243;409898;408793;724172;725923;410998;408423;724138;410399;552545;412528;551514;410195;100577365;413423;726458;411917;411744;408959;100578439;551928;413197;408306;409836;409904;409928;725091;408551;726681;411086;408891;551272;552589;410425;100576700;406123;724613;411124;726913;410487;724159;412763;410922;726582;408552;409942;413052;410502;410920;411238;410795;408405;411827;408602;725893;408359;408923;413054;410853;409778;410204;410271;552079;408821;100577801;411038;408383;410371;550930;408444;725782;408399;414025;408677;410222;410649;408981;408583;408434;726790;410211;727399;551356;410159;725294;408857;551895;100578515;411079;725776;726914;100578467;100578865;408292;408343;408365;408411;408429;408494;408508;408664;408758;408779;408862;409051;409658;409697;409733;409921;410228;410278;410902;412840;412968;413410;551360;552265;552804;724997;725932;726302;726729 | 4.30E-29 |
| GO:0044767 | single-organism developmental process | 411276;552187;724704;410925;552782;412801;409643;100576129;411569;409611;725928;408894;413558;410447;408509;413242;726419;410689;411158;408726;100577778;100578818;410769;410686;726461;408661;409708;411890;413987;409856;410723;408438;409305;410632;410956;410280;413968;552747;726842;552166;724607;725943;409001;408792;412878;724595;413569;727238;413683;408328;408763;409543;551733;413125;724460;551071;551746;411177;410685;413377;552485;725195;100577280;410730;410351;408777;727000;408810;409890;727235;409390;409707;411011;410368;100578826;551130;410279;408835;550804;411575;725754;408986;410258;408670;408786;408869;100576251;408890;552094;410739;552617;411969;552340;724991;408298;408936;409902;410606;413288;410975;412739;411250;412108;408264;724973;408276;408769;551765;726469;552377;410913;413215;408591;413677;725190;408430;725551;408431;409165;411981;409362;410674;724929;408278;724207;411112;412256;409084;724902;406084;410497;410785;409501;409893;413130;726709;409348;408348;413388;408900;412046;412884;551826;408852;411700;411048;409717;408976;410009;409405;726018;409024;409965;727312;408534;411539;406106;725679;408783;412602;413440;100578885;413246;410208;409983;408703;408406;410178;551602;406111;408616;409628;408896;413289;412406;408427;410340;550818;725038;726683;409049;411227;410906;725311;551291;551425;100578927;550966;410413;552235;725237;408489;727081;725436;724338;406154;726280;410933;724501;727335;411553;725155;411115;551848;412846;410174;413310;413618;410758;408631;409470;410514;409321;406122;725189;408939;410585;409854;410269;100576586;552397;411843;408325;410840;412175;409576;410427;408697;410253;412274;409006;724516;409961;412468;726587;408721;724344;724651;408951;410505;551735;724603;412770;413474;411812;408577;727528;410235;409151;410923;410554;412601;411175;410609;408372;409227;552078;409861;726350;408563;726510;410809;411157;409780;411566;100577697;550645;100578883;410732;406086;411116;411083;408470;410256;413371;100576321;410989;412742;552099;724322;408311;551124;725591;413385;724311;411288;413467;410203;551349;411678;724375;551894;408725;410767;413755;100578984;552709;409727;724518;100577393;724292;410776;410741;100578976;724851;408782;726349;726308;725391;725474;408723;411820;411144;551706;408844;724517;725433;410190;408277;410348;408519;551250;411009;410904;410918;408658;410553;100576392;409142;409722;410573;408358;551170;410951;551259;552030;551102;726375;411490;410326;408333;412243;409898;408793;724172;725923;410998;408423;724138;410399;552545;412528;551514;410195;100577365;413423;726458;411917;411744;408959;100578439;551928;413197;408306;409836;409904;409928;725091;408551;726681;411086;408891;551272;552589;410425;100576700;406123;724613;411124;726913;410487;724159;412763;410922;726582;408552;409942;413052;410502;410920;411238;410795;408405;411827;408602;725893;408359;408923;413054;410853;409778;410204;410271;552079;408821;100577801;411038;408383;410371;550930;408444;725782;408399;414025;408677;410222;410649;408981;408583;408434;726790;410211;727399;551356;410159;725294;408857;551895;100578515;411079;725776;726914;100578467;100578865;408292;408343;408365;408411;408429;408494;408508;408664;408758;408779;408862;409051;409658;409697;409733;409921;410228;410278;410902;412840;412968;413410;551360;552265;552804;724997;725932;726302;726729 | 9.00E-29 |
| GO:0048869 | cellular developmental process | 411276;552187;724704;552782;409643;411569;409611;725928;408894;413558;410447;726419;410689;411158;100577778;100578818;410769;410686;408661;411890;409856;410723;408438;410632;410280;552747;726842;552166;724607;725943;409001;408792;412878;724595;413569;727238;408328;408763;409543;551733;724460;551746;411177;410685;413377;100577280;410351;408777;727000;408810;409890;727235;409390;409707;410368;551130;410279;408835;550804;411575;725754;408986;408670;408786;408869;100576251;408890;552094;410739;552617;411969;552340;724991;408298;408936;409902;410975;412739;411250;412108;408264;724973;408276;551765;726469;552377;410913;413215;408591;725190;408430;408431;411981;409362;410674;724929;408278;724207;411112;412256;724902;406084;410497;410785;409501;409893;413130;726709;409348;413388;408900;412046;551826;408852;411700;411048;409717;408976;410009;409405;726018;409024;727312;408534;411539;406106;725679;408783;412602;413440;100578885;413246;410208;409983;408703;410178;551602;408616;408896;413289;412406;408427;410340;550818;725038;726683;409049;411227;410906;551291;100578927;550966;410413;552235;725237;727081;724338;406154;726280;410933;724501;727335;411553;411115;551848;413310;413618;410758;408631;409470;410514;409321;406122;725189;408939;410585;409854;410269;552397;411843;408325;410840;409576;410427;408697;410253;412274;409006;724516;409961;412468;408721;724651;408951;410505;724603;412770;411812;727528;410235;409151;410923;412601;411175;410609;408372;409227;552078;726350;408563;726510;410809;411157;409780;411566;100577697;100578883;406086;411116;411083;408470;410256;413371;100576321;412742;552099;408311;551124;725591;413385;411288;413467;410203;411678;724375;551894;408725;410767;413755;100578984;552709;409727;724518;100577393;724292;410776;410741;408782;726349;726308;725474;411820;411144;408844;724517;725433;410190;408277;410348;408519;551250;411009;410904;410918;410553;100576392;409142;409722;410573;408358;551170;410951;551259;552030;726375;410326;412243;409898;408793;724172;410998;408423;724138;410399;552545;412528;551514;410195;100577365;413423;726458;411917;411744;551928;409836;409904;725091;408551;726681;411086;551272;552589;410425;100576700;406123;724613;411124;726913;724159;412763;410922;726582;408552;409942;413052;411238;408405;411827;408602;408359;408923;413054;410853;409778;410204;410271;552079;100577801;411038;408383;410371;550930;725782;408399;414025;408677;410222;410649;408583;410211;727399;551356;410159;725294;408857;100578515;411079;725776;726914;100578467;408292;408343;408411;408494;408508;408758;408779;409051;409697;409733;409921;410228;410902;412840;412968;413410;552265;552804;724997 | 4.20E-28 |
| GO:0048468 | cell development | 411276;552187;724704;552782;409643;411569;409611;725928;408894;413558;410447;726419;410689;411158;410686;408661;411890;409856;410723;408438;410632;410280;726842;724607;725943;409001;408792;412878;724595;413569;727238;408328;408763;409543;551733;551746;411177;410685;413377;100577280;410351;408777;727000;408810;409890;727235;409390;409707;410368;551130;410279;408835;550804;725754;408986;408670;408786;408869;100576251;408890;410739;552617;411969;552340;724991;408298;408936;409902;410975;412739;411250;408264;408276;551765;726469;552377;413215;408591;725190;408430;408431;409362;410674;724929;408278;724207;411112;412256;724902;406084;410785;409501;409893;413130;726709;409348;413388;408900;412046;551826;408852;411700;411048;409717;408976;410009;409405;409024;727312;408534;411539;406106;725679;408783;412602;413440;100578885;413246;410208;409983;408703;410178;551602;408616;408896;413289;412406;408427;550818;725038;409049;411227;410906;551291;100578927;550966;410413;552235;724338;406154;726280;410933;724501;727335;411553;411115;551848;413310;413618;410758;408631;409470;409321;406122;725189;408939;410585;409854;552397;411843;408325;410840;409576;410427;408697;412274;724516;409961;412468;408721;724651;408951;410505;724603;412770;411812;727528;410235;409151;410923;412601;411175;410609;409227;726350;408563;410809;411157;409780;411566;100577697;100578883;406086;411116;408470;410256;413371;100576321;412742;552099;408311;551124;725591;413385;411288;413467;410203;411678;724375;408725;410767;413755;100578984;552709;409727;724518;100577393;724292;410776;410741;408782;726349;726308;725474;411820;411144;408844;724517;725433;410190;408277;410348;551250;411009;410904;410918;410553;100576392;409142;409722;410573;408358;410951;551259;552030;726375;410326;412243;409898;408793;724172;410998;724138;552545;412528;551514;410195;100577365;413423;411917;411744;551928;409836;409904;725091;408551;726681;551272;552589;410425;406123;724613;411124;724159;412763;410922;726582;408552;413052;411238;408602;408359;408923;413054;410853;409778;410204;552079;411038;410371;550930;725782;408399;414025;408677;410222;410649;408583;410211;727399;551356;725294;408857;100578515;411079;725776;726914;100578467;408292;408343;408411;408494;408508;408758;408779;409051;409697;409733;410228;410902;412840;412968;552265;552804 | 8.60E-28 |
| GO:0007275 | multicellular organism development | 411276;552187;724704;552782;412801;409643;100576129;411569;409611;725928;408894;413558;408509;413242;726419;410689;411158;408726;100578818;410686;726461;408661;409708;411890;413987;409856;410723;408438;410632;410956;410280;413968;726842;724607;725943;409001;408792;724595;413569;727238;413683;408328;408763;409543;551733;413125;551071;551746;411177;410685;413377;552485;725195;410730;410351;408777;727000;408810;409890;727235;409390;409707;411011;100578826;551130;410279;408835;550804;725754;410258;408670;408786;408869;100576251;408890;552094;410739;552617;411969;552340;724991;408298;408936;409902;410606;413288;410975;412739;411250;412108;408264;724973;408276;408769;551765;726469;552377;410913;413215;413677;725190;408430;725551;408431;409165;411981;409362;410674;724929;408278;724207;411112;412256;409084;724902;406084;410497;410785;409501;409893;413130;726709;409348;413388;408900;412046;412884;551826;408852;411700;411048;409717;408976;410009;727312;408534;411539;406106;725679;408783;412602;413440;100578885;413246;410208;409983;408703;408406;551602;406111;408616;409628;408896;413289;412406;408427;550818;725038;726683;409049;410906;725311;551291;551425;100578927;550966;410413;552235;725237;408489;725436;724338;406154;726280;410933;724501;727335;411553;725155;551848;412846;410174;413310;413618;410758;408631;409470;410514;409321;406122;725189;410585;410269;100576586;552397;408325;410840;412175;409576;410427;408697;410253;412274;409006;724516;409961;412468;726587;408721;724344;724651;408951;410505;551735;724603;412770;413474;411812;727528;410235;409151;410923;410554;412601;411175;410609;408372;409227;409861;726350;408563;726510;410809;411157;411566;100577697;550645;100578883;410732;406086;411116;411083;408470;410256;413371;410989;412742;552099;724322;408311;551124;725591;413385;724311;411288;413467;410203;551349;411678;724375;551894;408725;410767;413755;100578984;552709;724518;100577393;724292;410776;410741;100578976;724851;408782;726349;726308;725391;725474;408723;411820;411144;551706;408844;724517;725433;410190;408277;551250;411009;410904;410918;408658;410553;100576392;409142;409722;410573;408358;551170;410951;551259;552030;551102;411490;410326;408333;412243;409898;408793;724172;725923;410998;724138;410399;552545;412528;551514;100577365;413423;411917;411744;408959;551928;413197;408306;409836;409904;409928;725091;408551;726681;411086;408891;551272;552589;410425;100576700;406123;724613;411124;410487;724159;412763;410922;726582;408552;409942;413052;410502;410920;411238;408405;411827;408602;725893;408359;408923;410853;409778;410271;552079;408821;100577801;411038;408383;550930;408444;408399;414025;408677;410222;410649;408981;408583;408434;726790;410211;727399;551356;410159;725294;408857;551895;100578515;411079;725776;726914;100578467;100578865;408292;408343;408365;408411;408429;408494;408508;408664;408758;408779;408862;409051;409658;409697;409921;410228;410278;410902;412840;412968;413410;551360;552265;552804;724997;725932;726302;726729 | 3.70E-27 |
| GO:0030154 | cell differentiation | 411276;552187;724704;552782;409643;411569;409611;725928;408894;413558;410447;726419;410689;411158;100577778;100578818;410769;410686;408661;411890;409856;410723;408438;410632;410280;552747;726842;552166;724607;725943;409001;408792;412878;724595;413569;727238;408328;408763;409543;551733;724460;551746;411177;410685;413377;100577280;410351;408777;727000;408810;409890;727235;409390;409707;410368;551130;410279;408835;550804;725754;408986;408670;408786;408869;100576251;408890;552094;410739;552617;411969;552340;724991;408298;408936;409902;410975;412739;411250;412108;408264;724973;408276;551765;726469;552377;413215;408591;725190;408430;408431;411981;409362;410674;724929;408278;724207;411112;412256;724902;406084;410497;410785;409501;409893;413130;726709;409348;413388;408900;412046;551826;408852;411700;411048;409717;408976;410009;409405;726018;409024;727312;408534;411539;406106;725679;408783;412602;413440;100578885;413246;410208;409983;408703;410178;551602;408616;408896;413289;412406;408427;550818;725038;726683;409049;411227;410906;551291;100578927;550966;410413;552235;725237;724338;406154;726280;410933;724501;727335;411553;411115;551848;413310;413618;410758;408631;409470;410514;409321;406122;725189;408939;410585;409854;410269;552397;411843;408325;410840;409576;410427;408697;410253;412274;409006;724516;409961;412468;408721;724651;408951;410505;724603;412770;411812;727528;410235;409151;410923;412601;411175;410609;408372;409227;552078;726350;408563;726510;410809;411157;409780;411566;100577697;100578883;406086;411116;408470;410256;413371;100576321;412742;552099;408311;551124;725591;413385;411288;413467;410203;411678;724375;551894;408725;410767;413755;100578984;552709;409727;724518;100577393;724292;410776;410741;408782;726349;726308;725474;411820;411144;408844;724517;725433;410190;408277;410348;551250;411009;410904;410918;410553;100576392;409142;409722;410573;408358;551170;410951;551259;552030;726375;410326;412243;409898;408793;724172;410998;408423;724138;410399;552545;412528;551514;410195;100577365;413423;726458;411917;411744;551928;409836;409904;725091;408551;726681;411086;551272;552589;410425;100576700;406123;724613;411124;726913;724159;412763;410922;726582;408552;409942;413052;411238;408405;411827;408602;408359;408923;413054;410853;409778;410204;410271;552079;100577801;411038;408383;410371;550930;725782;408399;414025;408677;410222;410649;408583;410211;727399;551356;410159;725294;408857;100578515;411079;725776;726914;100578467;408292;408343;408411;408494;408508;408758;408779;409051;409697;409733;409921;410228;410902;412840;412968;413410;552265;552804;724997 | 4.70E-27 |
| GO:0009887 | animal organ morphogenesis | 411276;409611;725928;413558;413242;408661;410723;410632;410956;413968;726842;724607;408792;724595;413569;413683;408763;413125;551746;411177;410685;413377;410351;408810;409890;727235;411011;100578826;551130;408869;410739;552340;724991;408936;409902;412739;411250;412108;408264;408276;551765;726469;410913;413215;413677;725190;411981;724929;408278;409084;406084;410497;409501;726709;409348;412046;411700;411048;408976;410009;406106;412602;413440;100578885;413246;410208;409983;551602;408616;413289;412406;550818;726683;725311;725237;406154;726280;724501;411553;551848;410758;409470;409321;725189;410585;552397;410840;410427;410253;724516;409961;724651;551735;724603;413474;411812;727528;409151;410923;412601;410609;409227;726510;410809;411566;100577697;100578883;406086;411083;410256;413371;410989;724322;408311;551124;725591;413385;724311;551349;411678;100578984;552709;100577393;724292;410776;410741;724851;408782;725391;411820;551706;408844;724517;410190;408277;411009;410904;410918;410553;100576392;409722;408358;551170;410951;552030;410326;408333;412243;409898;408793;724172;725923;410399;552545;412528;100577365;413423;551928;413197;408306;409836;725091;408551;411086;551272;406123;724613;724159;410922;408552;409942;411238;408405;411827;408602;410853;410271;552079;411038;550930;408399;414025;408677;410649;726790;410211;727399;410159;551895;100578515;411079;725776;100578467;100578865;408292;408365;408429;408508;408664;408758;408779;408862;409658;409921;410228;412840;413410 | 9.60E-27 |
| GO:0060429 | epithelium development | 411276;724704;411569;409611;725928;413558;410447;413242;410689;100578818;408661;410723;410632;410956;413968;726842;724607;408792;724595;413569;408328;408763;413125;551746;411177;410685;552485;410351;727000;408810;727235;411011;410368;100578826;410279;408986;410258;408869;552617;552340;408936;410606;412739;412108;551765;726469;552377;413215;413677;725190;725551;408431;724929;408278;409084;724902;406084;410497;409501;409348;413388;412046;408976;410009;727312;406106;725679;408783;413440;100578885;413246;410208;410178;551602;408616;413289;550818;726683;409049;411227;410906;551291;551425;550966;725237;406154;726280;724501;411553;551848;409470;409321;725189;408939;410585;410269;552397;411843;408325;410840;412175;409576;410427;410253;724516;409961;726587;408721;724651;408951;410505;551735;724603;412770;410235;410923;412601;410609;408372;409227;409861;726510;410809;411157;411566;100577697;100578883;410256;413371;410989;724322;551124;725591;724311;410203;551349;411678;552709;100577393;724292;410776;410741;724851;411820;551706;408844;410190;408277;410348;411009;410904;410918;410553;100576392;551170;410951;552030;551102;410326;408333;412243;409898;408793;725923;410399;552545;412528;551514;410195;100577365;413423;408959;100578439;551928;413197;409904;409928;725091;408551;726681;411086;551272;552589;100576700;406123;724613;411124;410487;724159;412763;408552;409942;413052;410502;410920;411238;411827;408602;408359;413054;410271;552079;408821;100577801;411038;408383;410371;550930;408444;408399;414025;408677;410649;726790;410211;727399;410159;551895;100578515;411079;725776;100578467;100578865;408292;408411;408429;408494;408508;408664;408779;409658;409697;409733;409921;410228;410278;410902;412840;551360 | 2.10E-26 |
| GO:0044707 | single-multicellular organism process | 411276;552187;724704;552782;412801;726230;409643;100576129;411569;409611;725928;408894;413558;408509;413242;726419;410689;411158;408543;408726;100578818;410686;726461;408661;725996;409708;411890;413987;409856;410723;408438;409305;410632;410956;410280;413968;726842;724607;725943;409001;408792;724595;413569;727238;413683;408328;408763;409543;551733;413125;551071;551746;411177;410685;413377;552485;725195;410730;410351;727007;408777;727000;408810;409890;727235;409390;409707;411011;100578826;551130;410279;408835;550804;725754;410258;408670;408786;408869;100576251;408890;552094;410739;552617;411969;552340;724991;408298;408936;409902;410606;726361;413288;410975;412739;411250;412108;408264;724973;409726;408276;408769;551765;726469;552377;410913;413215;413677;725190;408430;725551;408431;409165;411981;409362;410674;724929;408278;724207;411112;412256;409084;724902;406084;410497;410785;409501;409893;413130;726709;100576770;552142;409348;408348;413388;408900;412046;412884;412059;551826;408852;411700;411048;409717;408976;410009;409405;409965;727312;408534;411539;406106;725679;408783;412602;413440;100578885;413246;410208;409983;408703;408406;410178;551602;406111;408616;409628;408896;413289;412406;408427;550818;725038;726683;409049;411227;410906;725311;551291;551425;100578927;550966;410413;552235;725237;408489;725436;724338;406154;726280;410933;724501;727335;411553;725155;411115;551848;412846;410174;413310;413618;410758;408631;409470;410514;409321;406122;725189;410585;726602;410269;100576586;552397;411843;408325;410840;412175;409576;410427;408697;410253;412274;409006;724516;409961;412468;726587;408721;724344;724651;408951;410505;551735;724603;412770;413474;413503;411812;408577;727528;410235;409151;410923;410554;412601;411175;410609;408372;409227;409861;726350;408563;726510;410809;411157;409780;413184;411566;100577697;550645;100578883;410732;406086;411116;411083;408470;410256;413371;410989;412742;552099;724322;408311;551124;725591;413385;724311;411288;413467;408331;410203;551349;411678;724375;551894;408725;410767;413755;100578984;552709;724518;100577393;724292;410776;410741;100578976;724851;408782;726349;726308;725391;725474;408723;411820;411144;551706;408844;724517;725433;410190;408277;551250;411009;410904;410918;408658;410553;100576392;409142;409722;410573;408358;551170;410951;551259;552030;551102;411490;410326;408333;412243;409898;408793;724172;725923;410998;408423;724138;410399;552545;412528;551514;410195;100577365;413423;726458;411917;411744;408959;551928;413197;408306;409836;409904;409928;725091;408551;408693;726681;411086;408891;551272;552589;410425;100576700;406123;724613;411124;410487;724159;412763;410922;726582;408552;409942;413052;410502;410920;411238;410795;408405;411827;408602;725893;408359;408923;413071;412470;410853;409777;409778;410271;552079;408821;100577801;411038;408383;410371;550930;408444;725782;408399;414025;408677;410222;410649;408981;408583;408434;726790;410211;727399;551356;410159;725294;408857;551895;100578515;411079;725776;726914;100578467;100578865;408292;408343;408365;408411;408429;408494;408508;408664;408758;408779;408862;409051;409658;409697;409921;410078;410228;410278;410902;411209;412840;412968;413410;551360;552265;552804;724997;725932;726302;726729 | 1.20E-25 |
| GO:0002009 | morphogenesis of an epithelium | 411276;724704;725928;413558;413242;408661;410723;410632;410956;413968;726842;724607;408792;724595;413125;411177;410685;552485;410351;408810;727235;411011;100578826;552617;552340;408936;410606;412108;551765;726469;413215;413677;725190;724929;409084;406084;410497;409501;409348;412046;410009;406106;100578885;413246;410208;551602;408616;413289;550818;726683;410906;725237;406154;726280;724501;551848;409470;409321;725189;410585;552397;411843;408325;412175;410427;410253;724516;409961;724651;410505;551735;724603;410235;410923;412601;409227;409861;726510;410809;411157;411566;100577697;100578883;410256;413371;410989;724322;551124;725591;724311;551349;411678;552709;100577393;724292;410776;410741;724851;411820;551706;408844;408277;411009;410904;410918;100576392;551170;410951;552030;410326;408333;412243;408793;725923;410399;552545;412528;410195;100577365;413423;408959;100578439;551928;413197;409904;725091;408551;411086;551272;552589;406123;724613;411124;724159;408552;413052;411238;411827;408602;408359;410271;552079;408821;100577801;411038;550930;408444;408399;414025;408677;410649;726790;410211;410159;551895;100578515;411079;725776;100578467;100578865;408292;408429;408508;408664;408779;409658;409697;409921;410228;410902;412840 | 8.40E-25 |
| GO:0007399 | nervous system development | 411276;552187;552782;412801;409643;100576129;409611;725928;408894;413558;408509;726419;410689;411158;410686;726461;408661;411890;410723;410956;410280;726842;724607;725943;409001;408792;724595;413569;408763;551733;551071;551746;411177;410685;413377;410351;408777;727000;408810;409890;727235;409390;409707;551130;408835;550804;725754;408670;408786;408869;100576251;408890;552094;410739;552617;552340;724991;408298;408936;409902;413288;410975;412739;411250;412108;408264;724973;410913;413215;725190;408430;409165;409362;410674;724929;408278;724207;411112;412256;724902;406084;410497;410785;409501;409893;413130;726709;409348;413388;408900;412046;551826;411700;409717;408976;410009;408534;406106;725679;408783;412602;413440;410208;409983;408703;551602;408896;413289;412406;408427;550818;725038;726683;409049;551291;100578927;410413;552235;725237;408489;406154;726280;727335;411553;551848;410174;413310;413618;410758;408631;409470;410514;409321;406122;725189;410585;552397;408325;410840;409576;410427;408697;410253;412274;724516;409961;408721;724651;408951;410505;724603;412770;411812;727528;410235;409151;412601;411175;410609;409227;726350;408563;726510;410809;100577697;100578883;410732;406086;411116;411083;408470;410256;413371;412742;552099;408311;551124;725591;413385;411288;413467;410203;411678;724375;551894;408725;410767;413755;100578984;724518;100577393;724292;410776;410741;100578976;408782;726349;726308;725474;411820;408844;725433;410190;551250;411009;410904;410918;410553;100576392;409142;409722;410573;410951;552030;411490;410326;412243;409898;408793;724172;410998;724138;552545;412528;551514;100577365;413423;411917;411744;551928;409836;409904;725091;411086;410425;100576700;724613;411124;724159;412763;410922;726582;409942;411238;408359;408923;409778;552079;550930;408399;414025;410222;410649;408981;408583;410211;727399;725294;408857;411079;725776;726914;100578467;408292;408343;408411;408494;408779;409051;409697;409921;410228;410902;412840;412968;413410;552265;724997;726302 | 8.90E-25 |
| GO:0032989 | cellular component morphogenesis | 411276;552187;552782;409643;411569;409611;725928;408894;413558;411158;410686;408661;411890;409856;410723;408438;410632;410280;726842;724607;725943;409001;724595;413569;408328;551733;551746;411177;410685;410351;408777;727000;408810;727235;409390;409707;410368;550804;411575;408670;408786;408869;408890;410739;552617;552340;408298;408936;409902;410975;412739;412108;408264;551765;410913;413215;725190;409362;410674;724929;408278;724207;411112;412256;406084;410785;409348;413388;408900;551826;409717;408976;410009;409024;727312;408534;406106;408783;100578885;413246;409983;408703;408896;413289;412406;408427;410340;550818;409049;410906;551291;100578927;550966;410413;552235;727081;406154;727335;411553;551848;413310;413618;409321;406122;725189;408939;410585;552397;408325;409576;410427;408697;412274;409961;412468;408721;724651;408951;410505;724603;412770;727528;410235;410923;412601;411175;410609;409227;408563;410809;409780;411566;411116;411083;408470;410256;413371;412742;552099;408311;551124;725591;411288;410203;411678;724375;408725;100578984;724518;100577393;410741;408782;726349;726308;411820;408844;725433;410190;408277;410348;408519;551250;411009;410904;410918;410573;551259;552030;410326;412243;408793;410998;724138;552545;412528;551514;100577365;413423;411917;411744;409836;409904;725091;551272;724613;724159;412763;410922;726582;411238;408359;408923;413054;409778;410204;550930;725782;408399;414025;410222;410649;408583;410211;725294;408857;100578515;411079;725776;726914;408343;408411;408494;408508;408779;409051;409697;409733;410902;412840;412968;552265 | 9.80E-25 |
| GO:0048729 | tissue morphogenesis | 411276;724704;725928;413558;413242;408661;410723;410632;410956;413968;726842;724607;408792;724595;413125;411177;410685;552485;410351;408810;727235;411011;100578826;552617;552340;408936;410606;412108;551765;726469;413215;413677;725190;724929;409084;406084;410497;409501;409348;412046;410009;406106;100578885;413246;410208;551602;408616;413289;550818;726683;410906;725237;406154;726280;724501;551848;409470;409321;725189;410585;552397;411843;408325;412175;410427;410253;724516;409961;724651;410505;551735;724603;410235;410923;412601;409227;409861;726510;410809;411157;411566;100577697;100578883;406086;410256;413371;410989;724322;551124;725591;724311;551349;411678;552709;100577393;724292;410776;410741;724851;411820;551706;408844;408277;411009;410904;410918;100576392;551170;410951;552030;410326;408333;412243;408793;725923;410399;552545;412528;410195;100577365;413423;408959;100578439;551928;413197;409904;725091;408551;411086;551272;552589;406123;724613;411124;724159;408552;413052;411238;411827;408602;408359;410271;552079;408821;100577801;411038;550930;408444;408399;414025;408677;410649;726790;410211;410159;551895;100578515;411079;725776;100578467;100578865;408292;408429;408508;408664;408779;409658;409697;409921;410228;410902;412840 | 2.70E-24 |
| GO:0032501 | multicellular organismal process | 411276;552187;724704;552782;412801;726230;409643;100576129;411569;409611;725928;408894;413558;410447;408509;413242;726419;410689;411158;408543;408726;100578818;410769;410686;726461;408661;725996;409708;411890;413987;409856;410723;408438;409305;410632;410956;410280;413968;726842;724607;725943;409001;408792;412878;724595;412917;413569;727238;408367;413683;100578770;408328;408763;409543;551733;413125;551071;551746;411177;410685;413377;552485;725195;100577280;410730;410351;727007;408777;727000;408810;409890;727235;409390;409707;411011;410368;100578826;551130;410279;408835;550804;100577515;725754;408986;410258;408670;408786;408869;100576251;408890;552094;725015;410739;552617;411969;409324;552340;724991;408298;408936;409902;410606;726361;413288;410975;412739;411250;412108;552149;408264;724973;409726;408276;408769;551765;726469;552377;410913;413215;408591;413677;408915;725190;408430;725551;408431;409165;411981;409362;410674;724929;408278;724207;411112;412256;409084;724902;406084;410497;410785;409501;409893;413130;726709;100576770;552142;409348;408348;413388;408900;412046;412884;412059;551826;552101;408852;411700;411048;409717;408976;410009;409405;409965;727312;408534;411539;406106;725679;408783;552259;412602;413440;100578885;413246;410208;409983;408703;408406;410178;551602;406111;408616;409628;408896;413289;412406;408427;550818;725038;726683;409049;411227;410906;725311;551291;551425;408855;100578927;550966;410413;552235;725237;408489;725436;724338;406154;726280;410933;724501;727335;408874;411553;725155;411115;551848;412846;410174;413310;413618;410758;408631;409470;410514;409321;406122;725189;408939;410585;726602;409854;410269;100576586;552397;411843;408325;410840;412175;409576;410427;408697;410253;412274;409006;724516;409961;412468;726587;408721;724344;724651;408951;410505;551735;724603;412770;413474;413503;411812;408577;727528;410235;409151;410923;410554;412601;411175;410609;408372;409227;552078;409861;726350;408563;726510;410809;411157;409780;413184;411566;100577697;550645;100578883;410732;406086;411116;411083;408470;410256;413371;100576321;410989;412742;552099;724322;408311;551124;725591;413385;724311;411288;413467;408331;413399;410203;551349;411678;724375;725900;551894;408725;410767;413755;100578984;552709;409727;724518;100577393;724292;410776;410741;100578976;724851;408782;726349;726308;725391;725474;408723;411820;411144;551706;408844;724517;725433;410190;408277;410348;551250;411009;410904;410918;408658;410553;100576392;409142;409722;410573;408358;551170;410951;551259;552030;551102;411490;410326;408333;412243;409898;408793;724172;725923;410998;408423;724138;410399;552545;408323;412528;551514;410195;100577365;413423;726458;411917;411744;408959;551928;413197;408306;409836;409904;409928;725091;408551;408693;726681;411086;408891;551272;552589;410425;100576700;406123;724613;411124;726913;410487;724159;412763;410922;726582;408552;409942;413052;410502;410920;411238;410795;408405;411827;408602;725893;408359;408923;413071;412470;413054;410853;409777;409778;410271;552079;408821;100577801;411038;408383;410371;550930;408607;408444;725782;408399;414025;408677;410222;410649;408981;408583;408434;726790;413319;410211;727399;551356;410159;725294;408857;551895;100578515;411079;725776;726914;100578467;100578865;408292;408343;408365;408411;408429;408494;408508;408664;408758;408779;408862;408973;409051;409658;409697;409733;409921;410078;410228;410278;410902;411209;412840;412968;413410;551360;552265;552804;724997;725932;726302;726729 | 3.00E-24 |
| GO:0009888 | tissue development | 411276;724704;411569;409611;725928;413558;410447;413242;410689;100578818;408661;410723;410632;410956;413968;726842;724607;408792;724595;413569;408328;408763;413125;551746;411177;410685;552485;410351;727000;408810;727235;411011;410368;100578826;410279;408986;410258;408869;552617;552340;408936;410606;412739;412108;551765;726469;552377;413215;413677;725190;725551;408431;724929;408278;409084;724902;406084;410497;409501;409348;413388;412046;408976;410009;727312;406106;725679;408783;413440;100578885;413246;410208;410178;551602;408616;413289;550818;726683;409049;411227;410906;551291;551425;550966;725237;406154;726280;724501;411553;551848;409470;409321;406122;725189;408939;410585;410269;552397;411843;408325;410840;412175;409576;410427;410253;724516;409961;726587;408721;724651;408951;410505;551735;724603;412770;413474;410235;410923;412601;410609;408372;409227;409861;726510;410809;411157;411566;100577697;100578883;406086;410256;413371;410989;724322;551124;725591;724311;410203;551349;411678;552709;100577393;724292;410776;410741;724851;411820;551706;408844;410190;408277;410348;411009;410904;410918;410553;100576392;551170;410951;551259;552030;551102;410326;408333;412243;409898;408793;725923;410399;552545;412528;551514;410195;100577365;413423;408959;100578439;551928;413197;409904;409928;725091;408551;726681;411086;551272;552589;100576700;406123;724613;411124;410487;724159;412763;408552;409942;413052;410502;410920;411238;411827;408602;408359;413054;410853;410271;552079;408821;100577801;411038;408383;410371;550930;408444;408399;414025;408677;410649;726790;410211;727399;551356;410159;551895;100578515;411079;725776;100578467;100578865;408292;408411;408429;408494;408508;408664;408779;409658;409697;409733;409921;410228;410278;410902;412840;551360;726302 | 3.90E-24 |
| GO:0048699 | generation of neurons | 411276;552187;552782;409643;409611;725928;408894;413558;726419;411158;410686;408661;411890;410723;410280;726842;724607;725943;409001;408792;724595;413569;551733;551746;411177;410685;413377;410351;408777;727000;408810;727235;409390;409707;551130;408835;550804;725754;408670;408786;408869;100576251;408890;410739;552617;552340;408298;408936;409902;410975;412739;411250;412108;408264;413215;725190;408430;409362;410674;724929;408278;724207;411112;412256;724902;406084;410497;410785;409501;409893;413130;726709;409348;413388;408900;412046;551826;411700;409717;410009;408534;406106;408783;412602;413440;410208;409983;408703;551602;408896;413289;412406;408427;550818;725038;726683;409049;551291;100578927;410413;552235;725237;406154;726280;727335;411553;551848;413310;413618;410758;408631;409470;410514;409321;725189;410585;552397;408325;410840;409576;410427;408697;410253;412274;724516;409961;408721;724651;408951;724603;412770;411812;727528;410235;409151;412601;411175;410609;409227;408563;410809;100577697;100578883;406086;411116;408470;410256;413371;412742;552099;408311;551124;725591;413385;411288;410203;411678;724375;408725;410767;413755;100578984;724518;100577393;724292;410741;408782;726349;726308;725474;411820;725433;410190;551250;411009;410904;410918;410553;100576392;409722;410573;552030;410326;412243;409898;408793;724172;410998;724138;552545;412528;551514;100577365;413423;411917;411744;551928;409836;409904;725091;724613;724159;412763;410922;726582;409942;411238;408359;408923;409778;552079;550930;408399;414025;410222;410649;408583;410211;727399;725294;408857;411079;725776;726914;100578467;408292;408343;408411;408494;408779;409051;409697;409921;410228;410902;412840;412968;552265;724997 | 1.30E-23 |
| GO:0050794 | regulation of cellular process | 411276;552187;724704;411534;410925;552782;412801;726230;100576129;552145;409611;725928;408894;413558;413242;410689;411158;408726;100577778;100578818;410686;726461;408661;725996;411890;409856;410723;408438;410632;410956;410280;413968;726842;724607;725943;409001;406096;412878;724595;413569;727238;413683;409774;408328;408763;409543;551733;413125;409027;551071;551746;411177;410685;409337;413377;725195;100577280;413280;410351;724442;408777;727000;408810;409890;727235;551707;409390;409707;411011;100578826;551130;408880;410279;408835;550804;411575;725754;410258;408670;725662;408786;408869;100576709;408890;552094;410739;552617;726399;552340;724991;408298;408936;409902;410606;413288;412739;411250;412108;408264;724973;408276;408769;551765;726469;552377;725393;410913;413215;408591;413677;408915;725190;408430;413171;725551;408431;411981;724929;408278;412256;409084;552585;724902;406084;413091;410497;410785;409501;413130;726709;725061;552142;409348;411794;410322;413388;408900;412046;412059;413318;551826;408852;411700;411048;551801;409717;408976;410009;409405;726018;409024;409788;409200;409965;727312;408534;411539;406106;725679;408783;552259;412602;413440;100578885;413246;410208;409983;408703;408406;551602;726159;406111;408616;408896;413289;410352;412406;408427;410828;410340;550818;725038;411765;409049;410906;725311;551291;409423;408855;100578927;550966;408597;410413;552235;725237;408489;725436;409862;406154;726280;410933;724501;727335;552289;411553;413562;725155;411115;551848;412846;410174;413310;100577367;410758;408631;409470;410514;409321;406122;725189;408939;411526;409854;413207;100576586;552397;411843;408325;410840;412002;412175;411159;409576;410427;726756;551822;410253;412274;409006;724516;409961;413183;726587;408721;724344;724651;408951;100578262;410505;551735;724603;412770;413474;411812;408577;727528;410235;409151;413510;410923;412601;411175;410609;409839;408372;409227;411754;726350;551587;408563;408275;726510;550811;410809;411157;409780;727008;411566;100577697;550645;100578883;408351;410732;406086;408803;411083;410256;413371;100576321;410989;412422;412742;552099;410567;724322;552732;408311;408944;551124;725591;413385;724311;408354;408528;411288;551521;413467;408331;410203;551349;411678;724375;551894;408725;100578984;552709;409727;100577393;724292;410776;724851;408918;726308;100577364;725474;411820;726251;411144;551706;408844;412887;413005;725433;410190;408277;408519;100578253;411009;410918;410553;100576392;409142;408358;551170;410951;551259;552030;727232;726375;410326;408333;412243;409898;408793;725923;410998;408423;724138;724559;410399;552545;412528;412357;551514;410195;100577365;413423;726458;411917;411744;551928;413197;409836;409904;409928;725091;726681;411086;408891;551272;552589;100576700;406123;724613;411124;726913;724159;412763;725290;410922;409942;413052;410502;411238;411827;408602;408359;408923;413071;412470;408388;413054;410853;408271;409777;725263;724592;410271;552079;100577801;411038;410371;550930;726506;408399;414025;408677;551094;410222;410649;408981;408583;410211;727399;410159;725294;100578515;411079;725776;726914;100578467;408292;408343;408411;408494;408508;408664;408729;408758;408779;409051;409697;409733;409921;410078;410228;410902;411209;411670;412840;413410;551360;552265;552804;724997;725932;726302;726729 | 3.60E-23 |
| GO:0030182 | neuron differentiation | 411276;552187;552782;409643;409611;725928;408894;413558;726419;411158;410686;408661;411890;410723;410280;726842;724607;725943;409001;408792;724595;413569;551733;551746;411177;410685;413377;410351;408777;727000;408810;727235;409390;409707;551130;408835;550804;408670;408786;408869;100576251;408890;410739;552617;552340;408298;408936;409902;410975;412739;411250;412108;408264;413215;725190;408430;409362;410674;724929;408278;724207;411112;412256;724902;406084;410785;409893;726709;413388;408900;412046;551826;411700;409717;410009;408534;406106;408783;412602;410208;409983;408703;551602;408896;413289;412406;408427;550818;725038;726683;409049;551291;100578927;410413;552235;725237;406154;726280;727335;411553;551848;413310;413618;410758;408631;409470;409321;725189;410585;552397;408325;410840;409576;410427;408697;410253;412274;724516;409961;408721;724651;408951;724603;412770;411812;727528;410235;409151;412601;411175;410609;409227;408563;410809;100577697;100578883;406086;411116;408470;410256;413371;412742;552099;408311;551124;725591;413385;411288;410203;411678;724375;408725;410767;413755;100578984;724518;100577393;410741;408782;726349;726308;725474;411820;725433;410190;551250;411009;410904;410918;410553;409722;410573;552030;410326;412243;409898;408793;724172;410998;724138;552545;412528;551514;100577365;411917;411744;551928;409836;409904;725091;724613;724159;412763;410922;726582;409942;411238;408359;408923;409778;552079;550930;408399;414025;410222;410649;408583;410211;725294;408857;411079;725776;726914;100578467;408343;408411;408494;408779;409051;409697;409921;410228;410902;412840;412968;552265 | 8.00E-23 |
| GO:0022008 | neurogenesis | 411276;552187;552782;409643;409611;725928;408894;413558;726419;411158;410686;408661;411890;410723;410280;726842;724607;725943;409001;408792;724595;413569;551733;551746;411177;410685;413377;410351;408777;727000;408810;727235;409390;409707;551130;408835;550804;725754;408670;408786;408869;100576251;408890;410739;552617;552340;408298;408936;409902;410975;412739;411250;412108;408264;413215;725190;408430;409362;410674;724929;408278;724207;411112;412256;724902;406084;410497;410785;409501;409893;413130;726709;409348;413388;408900;412046;551826;411700;409717;410009;408534;406106;408783;412602;413440;410208;409983;408703;551602;408896;413289;412406;408427;550818;725038;726683;409049;551291;100578927;410413;552235;725237;406154;726280;727335;411553;551848;413310;413618;410758;408631;409470;410514;409321;725189;410585;552397;408325;410840;409576;410427;408697;410253;412274;724516;409961;408721;724651;408951;410505;724603;412770;411812;727528;410235;409151;412601;411175;410609;409227;408563;410809;100577697;100578883;406086;411116;408470;410256;413371;412742;552099;408311;551124;725591;413385;411288;410203;411678;724375;408725;410767;413755;100578984;724518;100577393;724292;410741;408782;726349;726308;725474;411820;725433;410190;551250;411009;410904;410918;410553;100576392;409142;409722;410573;552030;410326;412243;409898;408793;724172;410998;724138;552545;412528;551514;100577365;413423;411917;411744;551928;409836;409904;725091;411086;410425;724613;411124;724159;412763;410922;726582;409942;411238;408359;408923;409778;552079;550930;408399;414025;410222;410649;408583;410211;727399;725294;408857;411079;725776;726914;100578467;408292;408343;408411;408494;408779;409051;409697;409921;410228;410902;412840;412968;552265;724997 | 1.20E-22 |
| GO:0000902 | cell morphogenesis | 411276;552187;552782;409643;409611;725928;408894;413558;411158;410686;408661;411890;410723;408438;410632;410280;726842;724607;725943;409001;724595;413569;551733;551746;411177;410685;410351;408777;727000;408810;727235;409390;409707;550804;411575;408670;408786;408869;408890;410739;552617;552340;408298;408936;409902;410975;412739;412108;408264;551765;410913;413215;725190;409362;410674;724929;408278;724207;411112;412256;406084;410785;409348;413388;408900;551826;409717;408976;410009;727312;408534;406106;408783;413246;408703;408896;413289;412406;408427;410340;550818;409049;410906;551291;100578927;410413;552235;727081;406154;727335;551848;413310;413618;409321;725189;408939;410585;552397;408325;409576;410427;408697;412274;409961;408721;724651;408951;410505;724603;412770;727528;410235;412601;411175;410609;409227;408563;410809;411566;411116;411083;408470;410256;413371;412742;552099;408311;551124;725591;411288;410203;411678;724375;408725;100578984;724518;410741;408782;726349;726308;411820;725433;410190;408277;408519;551250;411009;410904;410918;410573;552030;410326;412243;408793;410998;724138;552545;412528;551514;100577365;413423;411917;411744;409836;409904;725091;724613;724159;412763;410922;726582;411238;408359;408923;409778;550930;408399;414025;410222;410649;408583;410211;725294;408857;100578515;411079;725776;726914;408343;408411;408494;408508;408779;409051;409697;410902;412840;412968;552265 | 1.50E-22 |
| GO:0010468 | regulation of gene expression | 411276;552187;724704;411534;410925;726230;552145;408894;413558;410689;411158;408661;411890;410723;410956;726842;412878;724595;727238;408328;408763;413125;551071;551746;411177;409337;725195;100577280;409890;100578826;550804;725754;725662;408869;408890;552094;552617;726399;552340;408298;408936;410606;413288;410975;411250;408276;726469;725393;725190;725551;408431;724929;412256;409084;552585;724902;406084;413091;725352;409501;413130;725061;411794;413388;412046;412059;413318;551826;408852;411700;411048;551801;409717;410009;409405;409788;409200;409965;411539;725679;552259;412602;413440;100578885;410208;408406;551602;726159;406111;408616;413289;410352;412406;409049;725311;551425;409423;408855;100578927;408597;410413;552235;409862;726280;552289;411553;411115;412846;410174;100577367;410758;409470;409321;725189;411526;409854;100576586;552397;408325;410840;551822;410253;724516;409961;726587;100578262;410505;411905;724603;412770;413474;411812;727528;409151;412601;411175;409839;408372;409227;411754;726350;551587;408275;410809;409780;727008;100577697;100578883;408351;406086;410256;413371;100576321;410989;412422;412742;552099;410567;724322;552732;408944;724311;408354;551521;408331;410203;411678;724375;100578984;552709;100577393;724292;410776;724851;408918;725474;411820;411144;408844;411009;410918;410553;100576392;551170;410951;552030;726375;410326;412243;409898;408793;408423;410399;552545;412528;412357;100577365;726458;411917;551928;409928;408891;551272;100576700;406123;724613;724159;725290;410502;411238;408602;408923;413054;725263;724592;410271;552079;100577801;550930;408399;410222;408981;727399;410159;411079;725776;726914;408292;408411;408664;408758;409733;409921;410078;410228;412840;551360;726729 | 2.70E-22 |
| GO:0050789 | regulation of biological process | 411276;552187;724704;411534;410925;552782;412801;726230;100576129;552145;551796;409611;725928;408894;413558;413242;410689;411158;408726;100577778;100578818;410769;410686;726461;408661;725996;411890;413987;409856;410723;408438;409305;410632;410956;410280;413968;726842;724607;725943;409001;406096;412878;724595;413569;727238;413683;409774;408328;408763;409543;551733;413125;409027;551071;551746;411177;410685;409337;413377;725195;100577280;413280;410351;724442;408777;727000;408810;409890;727235;551707;409390;409707;411011;100578826;551130;408880;410279;408835;550804;411575;725754;410258;408670;725662;408786;408869;100576709;408890;552094;410739;552617;726399;552340;724991;408298;408936;409902;410606;413288;410975;412739;411250;412108;552149;408264;724973;408276;408769;551765;726469;552377;725393;410913;413215;408591;413677;408915;725190;408430;413171;725551;408431;411981;724929;408278;412256;409084;552585;724902;406084;413091;725352;410497;410785;409501;413130;726709;725061;552142;409348;411794;410322;408348;413388;408900;412046;412059;413378;413318;551826;408852;411700;411048;551801;409717;408976;410009;409405;726018;409024;409788;409200;409965;727312;408534;411539;406106;725679;408783;552259;412602;413440;100578885;413246;410208;409983;408703;408406;551602;726159;406111;408616;408896;413289;410352;412406;408427;410828;410340;550818;725038;411765;409049;410906;725311;551291;551425;409423;408855;100578927;550966;408597;410413;552235;725237;408489;725436;410838;409862;406154;726280;410933;724501;727335;552289;411553;413562;725155;411115;551848;412846;410174;413310;100577367;410758;408631;409470;410514;409321;406122;725189;408939;410585;411526;409854;413207;100576586;552397;411843;408325;410840;412002;412175;411159;409576;410427;726756;551822;410253;412274;409006;724516;409961;413183;726587;408721;724344;724651;408951;100578262;410505;551735;411905;724603;412770;413474;413503;411812;408577;727528;410235;409151;413510;410923;410554;412601;411175;410609;409839;408372;409227;552078;411754;726350;551587;408563;408275;726510;550811;410809;411157;409780;727008;411566;100577697;550645;100578883;408351;410732;406086;408803;411083;410256;413371;100576321;410989;412422;412742;552099;410570;410567;724322;552732;408311;408944;551124;725591;413385;724311;408354;408528;411288;551521;413467;408331;410203;551349;411678;724375;551894;408725;100578984;552709;409727;100577393;724292;410776;724851;408918;726308;100577364;725474;411820;726251;411144;551706;408844;412887;413005;725433;410190;408277;408519;100578253;411009;410918;410553;100576392;409142;409722;408358;551170;410951;551259;552030;727232;726375;411490;410326;408333;412243;409898;408793;725923;410998;408423;724138;724559;410399;552545;412528;412357;551514;412476;410195;100577365;413423;726458;411917;411744;551928;413197;409836;409904;409928;725091;726681;411086;408891;551272;552589;100576700;406123;724613;411124;726913;724159;412763;725290;410922;409942;413052;410502;411238;411827;408602;408359;408923;413071;412470;408388;413054;410853;408271;409777;725263;413574;724592;410204;410271;552079;100577801;411038;408383;410371;550930;726506;408399;414025;408677;551094;410222;410649;408981;408583;410793;726790;410211;727399;410159;725294;551895;100578515;411079;725776;726914;100578467;408292;408343;408411;408494;408508;408664;408729;408758;408779;409051;409697;409733;409921;410078;410228;410902;411209;411670;412840;413410;551360;552265;552804;724997;725932;726302;726729 | 6.70E-22 |
| GO:0000904 | cell morphogenesis involved in different... | 411276;552187;552782;409643;409611;408894;413558;411158;410686;408661;411890;410723;410632;726842;724607;409001;724595;413569;551746;411177;410685;410351;408777;727000;727235;409390;409707;550804;408670;408786;408869;408890;410739;552617;552340;408298;408936;409902;410975;412739;408264;551765;413215;725190;409362;410674;724929;408278;412256;406084;410785;413388;408900;551826;409717;408976;410009;406106;408783;413246;408703;408896;413289;408427;550818;409049;410906;551291;100578927;410413;552235;406154;727335;551848;413310;409321;725189;408939;410585;552397;408325;409576;412274;409961;724651;408951;410505;724603;412770;727528;410235;412601;411175;410609;409227;408563;410809;411116;413371;412742;552099;408311;551124;725591;411288;410203;411678;724375;408725;100578984;724518;410741;408782;726349;411820;725433;410190;408277;551250;411009;410904;410918;410573;552030;410326;412243;408793;410998;724138;552545;412528;100577365;411917;411744;409836;725091;724613;724159;410922;411238;408923;409778;550930;414025;410222;410649;408583;410211;725294;408857;100578515;411079;725776;726914;408343;408411;408508;408779;409051;409697;410902;412840;412968;552265 | 8.10E-22 |
| GO:0051252 | regulation of RNA metabolic process | 411276;552187;411534;410925;726230;552145;408894;413558;410689;411158;408661;411890;410723;726842;412878;727238;408328;408763;413125;551071;551746;411177;409337;725195;100577280;100578826;550804;725754;725662;408869;408890;552094;726399;552340;408298;408936;410606;413288;411250;726469;725393;725190;725551;408431;724929;412256;409084;552585;724902;406084;409501;413130;725061;411794;412046;412059;413318;551826;408852;411700;551801;409717;409788;409965;411539;725679;413440;100578885;410208;726159;406111;408616;413289;410352;412406;409049;725311;409423;408855;100578927;408597;410413;552235;409862;726280;552289;411553;412846;410174;100577367;410758;409470;409321;725189;411526;409854;100576586;552397;410840;551822;410253;724516;409961;726587;100578262;410505;724603;412770;413474;411812;727528;412601;411175;409839;408372;409227;411754;726350;551587;408275;410809;409780;727008;100578883;408351;406086;410256;413371;410989;412422;412742;552099;724322;552732;408944;724311;408354;551521;408331;410203;411678;724375;100578984;724292;410776;724851;408918;725474;411009;410918;410553;100576392;551170;410951;552030;726375;410326;412243;409898;408793;408423;410399;552545;412528;412357;100577365;726458;411917;551928;409928;408891;551272;100576700;406123;724159;725290;410502;411238;408602;408923;725263;724592;410271;552079;100577801;550930;408399;410222;408981;410159;411079;725776;726914;408292;408411;408758;409733;409921;410078;410228;412840;551360;726729 | 2.20E-21 |
| GO:0065007 | biological regulation | 411276;552187;724704;411534;410925;552782;412801;726230;100576129;552145;551796;409611;725928;408894;413558;413242;410689;411158;408726;100577778;100578818;410769;410686;726461;408661;725996;409708;411890;413987;409856;410723;408438;409305;410632;410956;410280;413968;726842;724607;725943;409001;408792;406096;412878;724595;413569;727238;413683;409774;408328;408763;409543;551733;413125;409027;551071;551746;411177;410685;409337;413377;410362;725195;100577280;413280;410730;410351;724442;408777;727000;408810;409890;727235;551707;409390;409707;411011;100578826;551130;408880;410279;408835;550804;411575;725754;410258;408670;725662;408786;408869;100576709;100576251;408890;552094;410739;552617;726399;552340;724991;408298;408936;409902;410606;408920;413288;409869;410975;412739;411250;412108;552149;408264;724973;408276;408769;551765;726469;552377;725393;410913;413215;408591;409043;413677;408915;725190;408430;413171;725551;408431;411981;724929;408278;412256;409084;552585;724902;406084;413091;725352;410497;410785;409501;413130;726709;725061;552142;409348;411794;410322;408348;413388;408900;412046;412059;413378;413318;551826;408852;411700;411048;551801;409717;408976;410009;409405;726018;409024;409788;409200;409965;727312;408534;411539;406106;725679;408783;552259;412602;413440;100578885;413246;410208;409983;408703;408406;551602;726159;406111;408616;409628;408896;413289;410352;412406;408427;410828;410340;550818;725038;412741;411765;409049;410906;725311;551291;551425;409423;408855;100578927;550966;408597;410413;552235;725237;408489;725436;410838;409862;406154;726280;410933;724501;727335;552289;411553;413562;725155;411115;551848;412846;410174;413310;100577367;410758;408631;409470;410514;409321;406122;725189;408939;410585;411526;409854;413207;100576586;552397;411843;408325;410840;412002;412175;411159;409576;410427;726756;551822;410253;412274;409006;724516;409961;413183;726587;408721;724344;724651;408951;410527;100578262;410505;551735;411905;724603;412770;413474;413503;411812;408577;727528;410235;409151;413510;410923;410554;412601;411175;410609;409839;408372;409227;552078;411754;409861;726350;551587;408563;408275;726510;550811;410809;411157;409780;727008;411566;100577697;550645;100578883;408351;410732;406086;408803;411083;410256;413371;100576321;410989;412422;412742;552099;410570;410567;724322;552732;408311;408944;551124;725591;413385;724311;408354;408528;411288;551521;413467;408331;410203;551349;411678;724375;551894;408725;410767;100578984;552709;409727;100577393;724292;410776;410741;724851;408918;726308;725391;100577364;725474;411820;726251;411144;551706;408844;412887;413005;725433;410190;408277;408519;100578253;551250;411009;410918;410553;100576392;409142;409722;408358;551170;410951;551259;552030;727232;726375;411490;410326;408333;412243;409898;408793;725923;410998;408423;724138;724559;410399;552545;412528;412357;551514;412476;410195;100577365;413423;726458;411917;411744;408959;551928;413197;408306;409836;409904;409928;725091;408551;408693;726681;406076;411086;408891;551272;552589;100576700;406123;724613;411124;726913;724159;412763;725290;410922;408552;409942;413052;410502;410920;411238;411827;408602;408359;408923;413071;412470;408388;413054;410853;408271;409777;725263;413574;724592;410204;410271;552079;100577801;411038;408383;410371;550930;725782;726506;408399;414025;408677;551094;410222;410649;408981;408583;410793;726790;410211;727399;551356;410159;725294;551895;100578515;411079;725776;726914;100578467;100578865;408292;408343;408365;408411;408429;408494;408508;408664;408729;408758;408779;408973;409051;409697;409733;409921;410078;410228;410278;410902;411209;411670;412840;413410;551360;552265;552804;724997;725932;726302;726729 | 5.80E-20 |
| GO:0019219 | regulation of nucleobase-containing comp... | 411276;552187;411534;410925;726230;552145;408894;413558;410689;411158;408661;411890;410723;726842;412878;727238;408328;408763;413125;551071;551746;411177;409337;725195;100577280;551707;100578826;550804;725754;725662;408869;408890;552094;726399;552340;408298;408936;410606;413288;411250;726469;725393;725190;725551;408431;724929;412256;409084;552585;724902;406084;410785;409501;413130;725061;411794;412046;412059;413318;551826;408852;411700;551801;409717;409788;409965;411539;725679;413440;100578885;410208;726159;406111;408616;413289;410352;412406;409049;725311;409423;408855;100578927;408597;410413;552235;409862;726280;552289;411553;412846;410174;100577367;410758;409470;409321;725189;411526;409854;100576586;552397;410840;409576;551822;410253;412274;724516;409961;726587;100578262;410505;724603;412770;413474;411812;727528;412601;411175;409839;408372;409227;411754;726350;551587;408275;410809;409780;727008;100578883;408351;406086;410256;413371;410989;412422;412742;552099;724322;552732;408944;724311;408354;551521;408331;410203;411678;724375;100578984;724292;410776;724851;408918;725474;411009;410918;410553;100576392;551170;410951;552030;726375;410326;412243;409898;408793;408423;410399;552545;412528;412357;100577365;726458;411917;551928;409928;408891;551272;100576700;406123;724159;725290;410502;411238;408602;408923;725263;724592;410271;552079;100577801;550930;408399;410222;408981;410159;411079;725776;726914;408292;408411;408758;409733;409921;410078;410228;412840;551360;726729 | 1.20E-19 |

**Supplemental Table S3.** List of GO data for 96hQ RNA-seq differences.

| **GO.ID** | **Term** | **Gene IDs** | **classicFisher** |
| --- | --- | --- | --- |
| GO:0140053 | mitochondrial gene expression | 725918;725274;409951;552097;725291;100577673;724263;408675;551812;725062;413398;412984;411351;725659;408352;409926;724744;412549;410727;551939;726000;410806;411924;552152;725496;551561;411618;726480;411103;552471;552025;551158;412473;724125;413121;411482;552676;413989;725357;100578551;552628;410941;100576395;552641;408392;413343;413928;551613;724164;551321;724493 | 5.70E-27 |
| GO:0042254 | ribosome biogenesis | 410200;408573;413805;726812;409951;724638;551992;411856;413404;411192;413686;552781;413935;726212;551383;552214;726537;550996;100576457;100578852;724352;725880;410879;726028;551235;100578005;551781;413313;724161;552664;725496;552280;552439;551087;410875;724742;724203;412190;408371;724125;410350;409589;410319;552241;413649;413591;552010;724142;724809;551311;411603;724506;409305;410280;408515;411953;411026;724493;725947;411233;727288;551386 | 6.10E-27 |
| GO:0006412 | translation | 725918;410200;408573;409951;552097;100578660;725291;726500;724263;408675;724704;552781;725062;413398;409258;552106;408441;552649;552494;412984;413909;411351;725659;726673;100576457;551235;408352;410414;409926;724744;412549;410727;551939;726000;726874;410806;413145;411924;552152;551629;725496;551901;726428;550903;411618;409560;410100;551184;412200;724532;726480;724868;726363;413011;410672;411103;552471;552025;551158;551242;412473;724125;413121;552056;411482;409589;552676;413989;725357;100578551;552241;724142;408556;552628;100576395;552641;408392;413343;551867;724733;413928;409550;552768;100576945;726056;552764;408515;727582;551613;724164;409842;551321;413796;724493;725943;409379 | 7.10E-26 |
| GO:0034660 | ncRNA metabolic process | 410200;408573;413805;551580;725274;724638;551992;411856;413404;410295;100577673;411192;413686;552781;413935;726212;551383;551812;550996;413909;100578852;725880;410879;726028;100578005;551781;413889;100578380;413313;726874;410806;552664;551629;725496;552439;551087;551561;550903;410875;412200;724742;551700;413011;412190;408371;552025;725234;411719;725326;410319;413209;413649;411509;410941;724809;551311;411603;408392;724733;409305;552768;551133;727582;551613;411026;413796;724493;725947;411233;724953;551745;727288;551386;413250 | 3.70E-24 |
| GO:0022613 | ribonucleoprotein complex biogenesis | 410200;408573;413805;726812;409951;724638;551992;100578660;411856;413404;411192;413686;552781;413935;726212;551383;552214;726816;726537;550996;726673;100576457;100578852;724352;725880;410879;726028;551235;100578005;551781;413313;724161;552664;725496;552280;552439;551087;409560;410875;724742;724203;412190;408371;724125;410350;409589;410319;552241;413649;413591;552010;724142;724809;551311;411603;724506;409305;410280;409544;408515;411953;408750;411026;724493;725947;411233;409379;727288;551386;725401 | 3.80E-23 |
| GO:0032543 | mitochondrial translation | 725918;409951;552097;725291;724263;408675;725062;413398;412984;411351;725659;408352;409926;724744;412549;410727;551939;726000;410806;411924;552152;411618;726480;411103;552471;552025;551158;412473;724125;413121;411482;552676;413989;725357;100578551;552628;100576395;552641;408392;413343;413928;551613;724164;551321;724493 | 6.90E-23 |
| GO:0043043 | peptide biosynthetic process | 725918;410200;408573;409951;552097;100578660;725291;726500;724263;408675;724704;552781;412610;412460;725062;413398;409258;552106;408441;552649;552494;412984;413909;411351;725659;726673;100576457;551235;408352;410414;409926;724744;412549;410727;551939;726000;726874;410806;413145;411924;552152;551629;725496;551901;726428;550903;411618;409560;410100;551184;412200;724532;726480;724868;726363;413011;410672;411103;552471;552025;551158;551242;412473;724125;413121;552056;411482;409589;552676;413989;725357;552672;100578551;552241;724142;408556;552628;100576395;552641;408392;413343;551867;724733;413928;409550;552768;100576945;726056;552764;408515;727582;551613;724164;409842;551321;413796;724493;725943;409379 | 9.00E-22 |
| GO:0034470 | ncRNA processing | 410200;408573;413805;551580;725274;724638;551992;411856;413404;410295;100577673;411192;413686;552781;413935;726212;551383;551812;550996;100578852;725880;410879;726028;100578005;551781;413889;100578380;413313;726874;552664;725496;552439;551087;410875;724742;551700;412190;408371;725234;411719;725326;410319;413209;413649;411509;410941;724809;551311;411603;409305;551133;551613;411026;724493;725947;411233;724953;551745;727288;551386;413250 | 1.70E-21 |
| GO:0043604 | amide biosynthetic process | 725918;410200;408573;409951;552097;100578660;725291;726500;724263;408675;724704;552781;412610;412460;725062;413398;409258;552106;408441;552649;551667;552494;412984;413909;411351;725659;726673;100576457;551235;408352;410414;409926;724744;412549;410727;551939;726000;726874;410806;413145;411924;552152;551629;725496;551901;726428;550903;411618;409560;410100;551184;412200;724532;726480;724868;726363;413011;410672;411103;552471;552025;551158;551242;412473;724125;413121;552056;411482;409589;552676;413989;725357;552672;100578551;552241;724142;408556;552628;100576395;552641;408392;413343;551867;724733;413928;409550;552768;100576945;726056;552764;408515;727582;551613;724164;409842;551321;413796;724493;725943;409379 | 2.00E-21 |
| GO:0043603 | cellular amide metabolic process | 725918;410200;408573;409951;552097;100578660;725291;726500;724263;408675;724704;552781;412610;412460;725062;413398;409258;552106;408441;552649;551667;552494;412984;413909;411351;725659;726673;100576457;551235;408352;410414;409926;724744;412549;410727;552118;551939;726000;726874;410806;413145;411924;552152;551629;725496;551901;726428;550903;411618;409560;410100;551184;412200;724532;726480;724868;726363;413011;410672;411103;552471;552025;551158;410769;551242;412473;724125;413121;552056;411482;409589;409708;411045;552676;413989;725357;552672;100578551;552241;724142;408556;552628;100576395;552641;408392;413343;551867;411796;724733;413928;409550;552768;551039;100576945;726056;552764;100577000;408515;727582;551613;724164;409842;551321;413796;724493;725943;409379;409870 | 2.60E-21 |
| GO:0006518 | peptide metabolic process | 725918;410200;408573;409951;552097;100578660;725291;726500;724263;408675;724704;552781;412610;412460;725062;413398;409258;552106;408441;552649;552494;412984;413909;411351;725659;726673;100576457;551235;408352;410414;409926;724744;412549;410727;552118;551939;726000;726874;410806;413145;411924;552152;551629;725496;551901;726428;550903;411618;409560;410100;551184;412200;724532;726480;724868;726363;413011;410672;411103;552471;552025;551158;410769;551242;412473;724125;413121;552056;411482;409589;411045;552676;413989;725357;552672;100578551;552241;724142;408556;552628;100576395;552641;408392;413343;551867;724733;413928;409550;552768;100576945;726056;552764;100577000;408515;727582;551613;724164;409842;551321;413796;724493;725943;409379;409870 | 4.80E-21 |
| GO:0006364 | rRNA processing | 410200;408573;413805;724638;551992;411856;413404;411192;413686;552781;413935;726212;551383;550996;100578852;725880;410879;726028;100578005;551781;413313;552664;725496;552439;551087;410875;724742;412190;408371;410319;413649;724809;551311;411603;409305;411026;724493;725947;411233;727288;551386 | 1.20E-17 |
| GO:0042273 | ribosomal large subunit biogenesis | 410200;408573;409951;413404;411192;552781;726212;551383;552214;100576457;726028;724161;552280;410875;724203;410350;409589;552241;413591;724142;551311;724506;409305;408515;411953;724493;551386 | 1.20E-16 |
| GO:0016072 | rRNA metabolic process | 410200;408573;413805;724638;551992;411856;413404;411192;413686;552781;413935;726212;551383;550996;100578852;725880;410879;726028;100578005;551781;413313;552664;725496;552439;551087;410875;724742;412190;408371;410319;413649;724809;551311;411603;409305;411026;724493;725947;411233;727288;551386 | 1.30E-16 |
| GO:1901566 | organonitrogen compound biosynthetic pro... | 725918;410200;408573;409951;552097;413708;100578660;413048;725291;726500;410295;724263;408675;724704;552781;412610;412460;725062;413398;409258;552106;408441;552649;551667;552494;412984;413909;411351;725659;726673;100576457;412458;552610;551235;408352;410414;726274;409926;724744;412549;410727;410039;552118;551939;726000;726874;410806;413145;408461;411924;552152;551629;551861;413605;725496;551901;551966;726428;550903;411618;409560;726120;410100;551184;412200;552682;724532;726118;551872;726480;724868;726363;413011;410672;411103;552471;552025;551158;551242;412473;724125;411719;413121;552056;413240;411482;409589;552676;413989;725357;550987;552672;100578551;552241;724142;408556;552628;100576395;413987;552641;408392;413343;551867;411796;724733;413928;411581;409550;552768;409299;100576945;726056;552764;408515;727582;551613;724164;413885;409842;725569;551321;552205;413796;724493;725943;409379 | 3.60E-13 |
| GO:0034641 | cellular nitrogen compound metabolic pro... | 408270;725918;410200;408573;413805;408368;413727;551580;725274;409951;552097;724638;551992;413708;552809;100578660;411856;413048;413404;725837;725291;726316;726500;410295;100577673;411192;724263;410030;413686;408675;724704;552781;412610;412460;413935;726212;551383;551812;725062;413398;726816;726537;410925;727210;409258;412328;552106;408441;552649;550996;551667;408984;552494;412984;411193;727049;412452;413909;725633;411351;725659;726673;100576457;100578852;552610;725880;552343;413542;725757;410879;726028;413006;551235;408352;410414;100578005;408527;551781;413889;726274;409926;412212;724744;412549;410727;100578380;552118;551939;726586;413313;726000;726874;410806;413014;552664;411757;551128;413145;408461;411924;552152;551629;551861;100577344;410893;413605;725496;410305;551901;551966;552439;551087;724355;551561;726428;550903;411618;411833;409560;408788;550935;408728;726120;410875;410100;551184;411122;412200;724742;411183;552682;724532;552076;551700;724628;551872;726480;724868;551990;726363;413011;412190;408371;410672;411103;552471;552025;551158;410769;411191;552424;724264;551242;412473;724125;725234;411719;413121;552056;724636;725326;411482;727471;409589;409708;410022;410319;411890;408909;411045;552676;413209;413989;725357;725142;411947;552672;100578551;552241;413649;411509;411510;413591;410266;551762;412578;724142;408556;552628;410941;724809;551311;411603;100576395;100359410;406077;552641;408392;413343;551867;409340;410452;409462;411796;724733;409305;413928;411581;725719;409544;409550;552768;409299;726666;551133;551039;100576945;726056;552764;100577000;409586;408515;411568;727582;726261;413815;551613;724164;409842;413891;408750;551321;411026;413796;413340;551904;100576912;724493;725943;725947;411233;551078;724953;409379;551745;727288;551386;725401;409870;413250;413814;408586 | 9.40E-13 |
| GO:0006399 | tRNA metabolic process | 551580;725274;551992;410295;100577673;551812;413909;100578852;413889;100578380;413313;726874;410806;551629;551561;550903;412200;551700;413011;552025;725234;411719;725326;411509;410941;408392;724733;552768;551133;727582;551613;413796;413250 | 3.00E-12 |
| GO:0006396 | RNA processing | 410200;408573;413805;551580;725274;724638;551992;411856;413404;725837;410295;100577673;411192;413686;724704;552781;413935;726212;551383;551812;408441;550996;100578852;725880;413542;410879;726028;410414;100578005;408527;551781;413889;100578380;413313;726874;552664;725496;552439;551087;551561;411833;550935;410875;411122;724742;552076;551700;724628;412190;408371;410672;725234;411719;725326;410319;411890;413209;725142;413649;411509;410941;724809;551311;411603;409462;409305;551133;413815;551613;408750;551321;411026;724493;725947;411233;724953;551745;727288;551386;725401;413250;413814 | 3.80E-11 |
| GO:0009451 | RNA modification | 413805;551580;724638;551992;410295;413686;552781;413935;726212;551812;100578005;413889;100578380;725496;724742;551700;725234;411719;725326;411509;410941;409305;551133;724493;413250 | 4.00E-11 |
| GO:0022900 | electron transport chain | 408270;552809;726316;412328;727049;552610;552343;412212;413014;100577344;413605;727599;411183;552424;724264;410022;408909;100359410;409586;551613;413891;413340;551904;551078 | 5.00E-10 |
| GO:0010467 | gene expression | 725918;410200;408573;413805;410343;551580;725274;409951;552097;724638;551992;100578660;411856;413404;725837;725291;726500;410295;100577673;411192;724263;410030;413686;408675;724704;552781;411163;413935;726212;551383;551812;725062;413398;726816;410925;409258;552106;408441;552649;550996;408984;552494;412984;411193;412452;413909;725633;411351;725659;726673;100576457;100578852;725880;413542;725757;410879;410890;726028;413006;551235;408352;410414;100578005;408527;551781;413889;409926;724744;412549;410727;100578380;551939;413313;726000;726874;410806;552664;551128;413145;411924;552152;551629;100577344;410893;725496;410305;551901;552439;551087;724355;551561;726428;550903;411618;411833;409560;550935;408728;410875;410100;551184;411122;412200;724742;724532;552076;551700;724628;410763;726480;724868;552175;726363;413011;412190;408371;410672;411103;552471;552025;551158;410769;411191;551242;412473;724125;725234;411719;413121;552056;724636;725326;411482;409589;410022;410319;411890;552676;413209;413989;725357;725142;411947;100578551;552241;413649;411509;411510;413591;412578;724142;408556;552628;410941;724809;551311;411603;100576395;406077;552641;408392;413343;551867;410452;409462;724733;409305;413928;725719;409544;409550;552768;726666;551133;100576945;726056;552764;408515;411568;727582;413815;551613;724164;409842;408750;551321;411026;413796;100576912;724493;725943;725947;411233;724953;409379;551745;727288;551386;725401;409870;413250;413814;408586 | 5.20E-10 |
| GO:0006119 | oxidative phosphorylation | 408270;552809;726316;412328;727049;552610;552343;412212;413014;100577344;413605;411183;552424;724264;410022;408909;100359410;409586;551613;413891;413340;551078 | 1.10E-09 |
| GO:0022904 | respiratory electron transport chain | 408270;552809;726316;412328;727049;552610;552343;412212;413014;100577344;413605;411183;552424;724264;410022;408909;100359410;409586;551613;413891;413340;551904;551078 | 1.20E-09 |
| GO:0042775 | mitochondrial ATP synthesis coupled elec... | 408270;552809;726316;412328;727049;552610;412212;413014;100577344;413605;411183;552424;724264;410022;408909;100359410;409586;413891;413340;551078 | 4.30E-09 |
| GO:0006413 | translational initiation | 408573;100578660;726500;409258;726673;551235;413145;551901;726428;409560;410100;551184;724532;551242;552056;408556;409550;552764;409842;409379 | 6.10E-09 |
| GO:0042773 | ATP synthesis coupled electron transport | 408270;552809;726316;412328;727049;552610;412212;413014;100577344;413605;411183;552424;724264;410022;408909;100359410;409586;413891;413340;551078 | 6.10E-09 |
| GO:0006626 | protein targeting to mitochondrion | 726498;412409;409991;725705;552727;551769;726081;408569;725240;409384;552175;409500;408284 | 1.20E-08 |
| GO:0065002 | intracellular protein transmembrane tran... | 412409;409991;725705;552727;551769;408569;725240;409384;552256;409500;412505;408284;726435 | 2.10E-08 |
| GO:0070585 | protein localization to mitochondrion | 726498;412409;409991;725705;552727;551769;726081;408569;725240;409384;552175;409500;408284 | 2.10E-08 |
| GO:0071806 | protein transmembrane transport | 412409;409991;725705;552727;551769;408569;725240;409384;552256;409500;412505;408284;726435 | 2.10E-08 |

**Supplemental Table S4.** List of GO data for 96hW RNA-seq differences.

| **GO.ID** | **Term** | **Gene IDs** | **classicFisher** |
| --- | --- | --- | --- |
| GO:0048731 | system development | 411276;408450;552187;408956;413616;412401;411986;413837;726710;725563;406139;552177;552782;412801;100578754;410718;551736;411018;408851;410393;409341;409847;409643;552075;100576129;100578450;727092;410060;408841;413782;411569;408447;725681;551433;551375;408716;409611;725928;411693;413557;408894;552062;413558;413370;412278;412353;409000;100577214;408509;725503;409817;413242;726671;726419;410689;411494;552185;724545;726936;411158;409753;410107;725910;409082;410108;408937;726014;552370;100578818;409003;410686;726461;408661;408435;408791;410655;551449;551666;725159;411212;412641;409366;413407;411201;409193;409856;411264;410723;409782;408438;409063;410632;726017;410956;724281;413908;408718;409676;412003;413968;551290;726405;551868;552742;724853;410499;408688;408645;411181;410982;726842;410161;410694;724607;413133;100576326;409001;725140;726053;408792;551947;410257;410710;551914;408963 | 1.30E-10 |
| GO:0007399 | nervous system development | 411276;408450;552187;408956;413616;412401;413837;726710;725563;406139;552177;552782;412801;410718;551736;411018;410393;409341;409847;409643;100576129;727092;410060;408841;408447;725681;551433;408716;409611;725928;411693;413557;408894;552062;413558;413370;412278;412353;409000;100577214;408509;725503;409817;726419;410689;411494;552185;724545;726936;411158;409753;410107;725910;409082;726014;552370;409003;410686;726461;408661;408435;410655;551449;551666;411212;412641;409366;411201;411264;410723;409782;409063;726017;410956;724281;408718;409676;412003;726405;551868;552742;410499;408688;408645;411181;726842;410161;410694;724607;413133;100576326;409001;725140;408792;551947;551914 | 5.30E-10 |
| GO:0032989 | cellular component morphogenesis | 411276;408450;552187;408956;412401;413837;725563;406139;552782;410718;411018;409341;409847;409643;727092;410060;411569;408447;725681;551433;408716;409611;410087;725928;411693;413557;408894;552062;413558;412197;413370;412353;409000;725503;409817;726671;411494;724545;726936;411158;410107;724488;725910;409082;410686;408661;408435;410655;411212;412641;411201;409856;411264;410723;408438;410632;726017;724281;408718;410353;409676;726405;551868;552742;410499;408688;408645;726842;410161;410694;724607;100576326;409001;725140;551914 | 2.60E-09 |
| GO:0007275 | multicellular organism development | 411276;413833;726331;408450;552187;408956;413616;412401;409376;411986;413837;726710;725563;406139;552177;552782;412801;100578754;410718;551736;412115;411018;408851;410393;409341;409847;409643;552075;100576129;100578450;727092;410060;408841;410757;413782;411569;408447;725681;551433;551375;408716;409611;410087;725928;411693;100576432;413557;408894;552062;412008;413558;412197;413370;412278;412353;409000;100577214;408509;725503;409817;413242;726671;726419;410689;411494;552185;724545;726936;411158;409753;410107;724488;725910;409082;410108;408937;726014;552370;409682;100578818;409003;410686;726461;408661;408435;408791;410655;551449;551666;725159;411212;412641;409366;413407;411201;409193;409856;411264;410723;409782;408438;409063;410632;726017;410956;724281;413908;408718;409676;412003;413968;551290;726405;551868;552742;724853;410499;408688;408645;411181;410982;726842;410161;410694;724607;413133;100576326;409001;725140;726053;408792;551947;410257;410710;551914;408963 | 8.80E-09 |
| GO:0048812 | neuron projection morphogenesis | 411276;408450;552187;408956;412401;413837;725563;406139;552782;410718;411018;409847;409643;727092;410060;408447;725681;551433;408716;409611;411693;413557;408894;413558;413370;412353;409000;725503;409817;411494;724545;726936;411158;410107;725910;409082;410686;408661;408435;410655;411212;412641;411201;411264;410723;726017;724281;408718;409676;726405;551868;552742;410499;408688;408645;726842;410694;724607;100576326;409001;725140;551914 | 1.30E-08 |
| GO:0048858 | cell projection morphogenesis | 411276;408450;552187;408956;412401;413837;725563;406139;552782;410718;411018;409847;409643;727092;410060;408447;725681;551433;408716;409611;411693;413557;408894;413558;413370;412353;409000;725503;409817;411494;724545;726936;411158;410107;725910;409082;410686;408661;408435;410655;411212;412641;411201;411264;410723;726017;724281;408718;409676;726405;551868;552742;410499;408688;408645;726842;410694;724607;100576326;409001;725140;551914 | 1.30E-08 |
| GO:0120039 | plasma membrane bounded cell projection ... | 411276;408450;552187;408956;412401;413837;725563;406139;552782;410718;411018;409847;409643;727092;410060;408447;725681;551433;408716;409611;411693;413557;408894;413558;413370;412353;409000;725503;409817;411494;724545;726936;411158;410107;725910;409082;410686;408661;408435;410655;411212;412641;411201;411264;410723;726017;724281;408718;409676;726405;551868;552742;410499;408688;408645;726842;410694;724607;100576326;409001;725140;551914 | 1.30E-08 |
| GO:0032990 | cell part morphogenesis | 411276;408450;552187;408956;412401;413837;725563;406139;552782;410718;411018;409847;409643;727092;410060;408447;725681;551433;408716;409611;411693;413557;408894;413558;413370;412353;409000;725503;409817;411494;724545;726936;411158;410107;725910;409082;410686;408661;408435;410655;411212;412641;411201;411264;410723;726017;724281;408718;409676;726405;551868;552742;410499;408688;408645;726842;410694;724607;100576326;409001;725140;551914 | 1.80E-08 |
| GO:0048468 | cell development | 552202;411276;408450;552187;408956;412401;413837;725563;406139;552177;552782;100578754;410718;551736;412115;411018;408851;409341;409847;409643;552075;727092;410060;411569;408447;725681;551433;408716;409611;410087;725928;411693;413557;408894;552062;413558;412197;413370;412278;412353;409000;100577214;725503;409817;726419;410689;411494;724545;726936;411158;409753;410107;724488;725910;409082;552370;410686;408661;408435;408791;410655;551449;551666;411219;411212;412641;411201;409193;100578281;409856;411264;410723;409782;408438;410632;726017;724281;408718;410353;409676;412003;551290;726405;551868;552742;410499;408688;408645;411181;410982;726842;410694;100578429;724607;413133;413332;100576326;409001;725140;408792;551947;410257;410710;551914 | 1.80E-08 |
| GO:0000902 | cell morphogenesis | 411276;408450;552187;408956;412401;413837;725563;406139;552782;410718;411018;409847;409643;727092;410060;408447;725681;551433;408716;409611;725928;411693;413557;408894;413558;413370;412353;409000;725503;409817;726671;411494;724545;726936;411158;410107;725910;409082;410686;408661;408435;410655;411212;412641;411201;411264;410723;408438;410632;726017;724281;408718;409676;726405;551868;552742;410499;408688;408645;726842;410161;410694;724607;100576326;409001;725140;551914 | 2.40E-08 |
| GO:0050789 | regulation of biological process | 409667;411276;726331;408450;552187;408956;413616;412401;409376;411986;411534;413837;725485;726710;725563;406139;552463;552177;552782;412801;100578754;410718;551736;412115;726230;409341;552075;100576129;552145;100577833;409795;100578450;552309;727092;410060;408841;410757;551796;725681;551375;408716;409138;409611;410087;725928;411693;410006;413557;408894;552062;412008;413558;406082;412197;409565;412278;412353;409000;100577214;412104;552721;725503;409817;413242;410950;726671;410689;411494;552185;724545;726936;411158;409753;410107;724488;725910;410108;408937;726014;552370;409682;100577778;100578818;727479;409003;410686;411617;726461;408661;408435;725996;408791;412355;410655;551449;551666;725159;411219;408557;408955;726003;551636;726651;411212;412641;409366;413407;411201;409193;100578281;408614;409856;411264;410723;409255;409782;409579;408438;551930;410632;726017;410956;724281;413908;408718;410353;409676;414017;409957;412003;552243;726438;413968;409514;551290;726405;551868;552742;724853;410499;408688;408645;411181;410982;726842;410161;410694;100578429;724607;408879;413133;100576326;409001;412265;725140;726053;551947;410710;406096;551914;408963 | 3.00E-08 |
| GO:0031175 | neuron projection development | 411276;408450;552187;408956;412401;413837;725563;406139;552782;410718;411018;409847;409643;727092;410060;408447;725681;551433;408716;409611;411693;413557;408894;413558;413370;412353;409000;725503;409817;411494;724545;726936;411158;410107;725910;409082;410686;408661;408435;410655;411212;412641;411201;411264;410723;726017;724281;408718;409676;726405;551868;552742;410499;408688;408645;726842;410694;724607;100576326;409001;725140;551947;551914 | 3.40E-08 |
| GO:0050794 | regulation of cellular process | 409667;411276;726331;408450;552187;408956;413616;411986;411534;413837;725485;726710;725563;406139;552177;552782;412801;100578754;410718;551736;412115;726230;409341;552075;100576129;552145;100577833;409795;100578450;552309;727092;410060;408841;410757;725681;408716;409138;409611;410087;725928;411693;410006;413557;408894;552062;412008;413558;406082;412197;412278;412353;409000;100577214;412104;552721;725503;409817;413242;410950;726671;410689;411494;552185;724545;726936;411158;409753;410107;724488;725910;408937;726014;552370;409682;100577778;100578818;727479;409003;410686;411617;726461;408661;408435;725996;408791;412355;410655;551449;551666;411219;408557;408955;726003;551636;726651;411212;412641;409366;413407;411201;409193;100578281;408614;409856;411264;410723;409255;409579;408438;551930;410632;726017;410956;724281;408718;410353;409676;414017;409957;412003;552243;726438;413968;409514;551290;726405;551868;552742;724853;410499;408688;408645;411181;410982;726842;410161;410694;100578429;724607;408879;413133;100576326;409001;412265;725140;726053;551947;410710;406096;551914;408963 | 4.50E-08 |
| GO:0048856 | anatomical structure development | 552202;411276;413833;726331;408450;552187;408956;413616;412401;409376;411986;413837;726710;725563;406139;552177;552782;412801;100578754;410718;551736;412115;411018;408851;410393;409341;409847;409643;552075;100576129;100578450;727092;410060;408841;410757;413782;411569;408447;725681;551433;551375;408716;409611;410087;725928;411693;100576432;413557;408894;552062;412008;413558;412197;413370;412278;412353;409000;100577214;408509;725503;409817;413242;726671;726419;410689;411494;552185;724545;726936;411158;409753;410107;724488;725910;409082;410108;408937;726014;552370;409682;100578818;409003;410686;726461;408661;408435;408791;410655;551449;551666;725159;411219;411212;412641;409366;413407;411201;409193;100578281;409856;411264;410723;409782;408438;409063;410632;726017;410956;724281;413908;408718;410353;409676;412003;413968;551290;726405;551868;552742;724853;410499;408688;408645;411181;410982;726842;410161;410694;100578429;724607;413133;413332;100576326;409001;725140;726053;408792;551947;410257;410710;551914;408963 | 5.70E-08 |
| GO:0009653 | anatomical structure morphogenesis | 411276;726331;408450;552187;408956;412401;411986;413837;726710;725563;406139;552177;552782;100578754;410718;551736;412115;411018;408851;409341;409847;409643;552075;727092;410060;411569;408447;725681;551433;408716;409611;410087;725928;411693;100576432;413557;408894;552062;413558;412197;413370;412278;412353;409000;100577214;725503;409817;413242;726671;410689;411494;724545;726936;411158;409753;410107;724488;725910;409082;408937;410686;408661;408435;408791;410655;411212;412641;413407;411201;409193;409856;411264;410723;409782;408438;410632;726017;410956;724281;408718;410353;409676;412003;413968;551290;726405;551868;552742;724853;410499;408688;408645;410982;726842;410161;410694;724607;413133;100576326;409001;725140;408792;410257;551914;408963 | 7.50E-08 |
| GO:0030154 | cell differentiation | 552202;411276;408450;552187;408956;412401;409376;411986;413837;725563;406139;552177;552782;100578754;410718;551736;412115;411018;408851;409341;409847;409643;552075;552309;727092;410060;411569;408447;725681;551433;408716;409611;410087;725928;411693;413557;408894;552062;413558;412197;413370;412278;412353;409000;100577214;725503;409817;726671;726419;410689;411494;724545;726936;411158;409753;410107;724488;725910;409082;726014;552370;100577778;100578818;410686;408661;408435;408791;410655;551449;551666;725159;411219;411212;412641;411201;409193;100578281;409856;411264;410723;409782;408438;410632;726017;724281;413908;408718;410353;409676;412003;551290;726405;551214;551868;552742;410499;408688;408645;411181;410982;726842;410161;410694;100578429;724607;413133;413332;100576326;409001;725140;408792;551947;410257;410710;551914 | 1.10E-07 |
| GO:0032501 | multicellular organismal process | 552202;411276;413833;726331;408450;552187;413759;408956;413616;412401;409376;411986;413837;726710;725563;406139;552463;552177;552782;412801;100578754;410718;551736;412115;411018;406147;408851;410393;409341;409847;409643;552075;100576129;100578450;408576;727092;410060;408841;410757;413782;411569;408447;725681;551433;551375;408716;409611;410087;725928;411693;100576432;410006;413557;408894;552062;412008;413558;406082;412197;413370;412278;412353;409000;552070;100577214;408509;412104;725503;409817;413242;726671;726419;410689;411494;552185;724545;726936;411158;409753;410107;408543;724488;725910;409082;410108;408937;726014;552370;409682;100578818;409003;410686;411617;726461;408661;408435;725996;408791;725689;410655;551449;551666;725159;411219;408955;411212;412641;409366;413407;411201;409193;100578281;552711;409856;411264;410723;409782;408438;409063;410632;726017;410956;724281;413908;408718;410353;409676;414017;412003;411725;413968;551290;726405;551868;552742;724853;410499;408688;408645;411181;410982;726842;410161;410694;100578429;724607;413133;413332;100576326;409001;725140;726053;408792;551947;410257;410710;551914;408963 | 2.00E-07 |
| GO:0022008 | neurogenesis | 411276;408450;552187;408956;412401;413837;725563;406139;552177;552782;410718;551736;411018;409341;409847;409643;727092;410060;408447;725681;551433;408716;409611;725928;411693;413557;408894;413558;413370;412353;409000;100577214;725503;409817;726419;411494;724545;726936;411158;409753;410107;725910;409082;552370;410686;408661;408435;410655;551449;551666;411212;412641;411201;411264;410723;409782;726017;724281;408718;409676;412003;726405;551868;552742;410499;408688;408645;411181;726842;410161;410694;724607;413133;100576326;409001;725140;408792;551947;551914 | 2.30E-07 |
| GO:0048869 | cellular developmental process | 552202;411276;408450;552187;408956;412401;409376;411986;413837;725563;406139;552177;552782;100578754;410718;551736;412115;411018;408851;409341;409847;409643;552075;552309;727092;410060;411569;408447;725681;551433;408716;409611;410087;725928;411693;413557;408894;552062;413558;412197;413370;412278;412353;409000;100577214;725503;409817;726671;726419;410689;411494;724545;726936;411158;409753;410107;724488;725910;409082;726014;552370;100577778;100578818;410686;408661;408435;408791;410655;551449;551666;725159;411219;411212;412641;411201;409193;100578281;409856;411264;410723;409782;408438;410632;726017;724281;413908;408718;410353;409676;412003;551290;726405;551214;551868;552742;410499;408688;408645;411181;410982;726842;410161;410694;100578429;724607;413133;413332;100576326;409001;725140;408792;551947;410257;410710;551914 | 2.30E-07 |
| GO:0120036 | plasma membrane bounded cell projection ... | 411276;408450;552187;408956;412401;413837;725563;406139;552782;410718;411018;409341;409847;409643;727092;410060;408447;725681;551433;408716;409611;411693;413557;408894;413558;413370;412353;409000;552070;725503;409817;411494;724545;726936;411158;410107;725910;409082;410686;408661;408435;410655;411219;411212;412641;411201;411264;410723;726017;724281;408718;409676;726405;551868;552742;410499;408688;408645;726842;410694;100578429;724607;100576326;409001;725140;408792;551947;551914 | 2.70E-07 |
| GO:0016043 | cellular component organization | 409667;411276;408450;552187;408956;412401;411986;413837;725563;406139;552463;552177;552782;100578754;410718;551736;412115;411018;409341;409847;409643;552075;100577833;409795;100578450;408576;552309;727092;410060;408841;411569;408447;725681;551433;408716;409138;409611;410087;725928;411693;100576432;413557;408894;552062;413558;412197;413370;412278;412353;409000;552070;408509;412104;725503;409817;413242;410950;726671;726419;410689;411494;724545;726936;411158;410107;724488;725910;409082;408937;726014;552370;100577778;100578818;409003;410686;726461;408661;408435;408686;408791;412355;410655;551449;551666;411219;408557;408955;411212;412641;411201;409193;409856;411264;410723;409255;409782;409579;408438;409063;410632;726017;410956;724281;408718;410353;409676;409957;412003;552243;726438;411725;409514;551290;726405;551868;552742;724853;410499;408688;408645;410982;726842;410161;410694;100578429;724607;413133;413332;100576326;409001;412265;725140;726053;408792;551947;410257;551914 | 3.00E-07 |
| GO:0032502 | developmental process | 552202;411276;413833;726331;408450;552187;408956;413616;412401;409376;411986;411534;413837;726710;725563;406139;552177;552782;412801;100578754;410718;551736;412115;411018;408851;410393;409341;409847;409643;552075;100576129;100578450;552309;727092;410060;408841;410757;413782;411569;408447;725681;551433;551375;408716;409611;410087;725928;411693;100576432;413557;408894;552062;412008;413558;412197;413370;412278;412353;409000;100577214;408509;725503;409817;413242;726671;726419;410689;411494;552185;724545;726936;411158;409753;410107;724488;725910;409082;410108;408937;726014;552370;409682;100577778;100578818;409003;410686;726461;408661;408435;408791;410655;551449;551666;725159;411219;411212;412641;409366;413407;411201;409193;100578281;409856;411264;410723;409782;408438;409063;410632;726017;410956;724281;413908;408718;410353;409676;412003;413968;551290;726405;551214;551868;552742;724853;410499;408688;408645;411181;410982;726842;410161;410694;100578429;724607;413133;413332;100576326;409001;725140;726053;408792;551947;410257;410710;551914;408963 | 3.10E-07 |
| GO:0030030 | cell projection organization | 411276;408450;552187;408956;412401;413837;725563;406139;552782;410718;411018;409341;409847;409643;727092;410060;408447;725681;551433;408716;409611;411693;413557;408894;413558;413370;412353;409000;552070;725503;409817;411494;724545;726936;411158;410107;725910;409082;410686;408661;408435;410655;411219;411212;412641;411201;411264;410723;726017;724281;408718;409676;726405;551868;552742;410499;408688;408645;726842;410694;100578429;724607;100576326;409001;725140;408792;551947;551914 | 3.30E-07 |
| GO:0009605 | response to external stimulus | 411276;726331;408450;413759;413837;725563;406139;552782;412115;726230;409341;727092;408841;410757;551796;408447;551433;408716;409611;410087;413557;552062;413558;412197;413370;409565;412278;409000;408509;725503;409817;409039;411113;410689;411494;724545;724488;409082;551758;410686;408661;408435;408791;410655;551449;725159;411212;411201;552711;410723;409782;408438;409063;410632;726017;410956;413908;408718;409676;726405;552742;724853;408688;411181;410982;408481;726842;410694;724607;408879;100576326;409001;725140;551914 | 3.30E-07 |
| GO:0048699 | generation of neurons | 411276;408450;552187;408956;412401;413837;725563;406139;552177;552782;410718;551736;411018;409341;409847;409643;727092;410060;408447;725681;551433;408716;409611;725928;411693;413557;408894;413558;413370;412353;409000;100577214;725503;409817;726419;411494;724545;726936;411158;409753;410107;725910;409082;410686;408661;408435;410655;551449;551666;411212;412641;411201;411264;410723;409782;726017;724281;408718;409676;412003;726405;551868;552742;410499;408688;408645;726842;410694;724607;413133;100576326;409001;725140;408792;551947;551914 | 3.50E-07 |
| GO:0030182 | neuron differentiation | 411276;408450;552187;408956;412401;413837;725563;406139;552782;410718;551736;411018;409341;409847;409643;727092;410060;408447;725681;551433;408716;409611;725928;411693;413557;408894;413558;413370;412353;409000;725503;409817;726419;411494;724545;726936;411158;409753;410107;725910;409082;410686;408661;408435;410655;551449;411212;412641;411201;411264;410723;409782;726017;724281;408718;409676;412003;726405;551868;552742;410499;408688;408645;726842;410694;724607;100576326;409001;725140;408792;551947;551914 | 4.90E-07 |
| GO:0040011 | locomotion | 726331;408450;408956;413837;725563;406139;727092;411569;408447;551433;408716;409611;725928;413557;413558;413370;409000;725503;409817;413242;411494;724545;726936;410107;409082;410686;408661;408435;408791;410655;411219;409193;100578281;410723;726017;410956;724281;408718;409676;411725;726405;551868;552742;724853;410499;408688;411181;410982;408481;726842;100578429;724607;408879;409001;725140;410710;551914 | 7.60E-07 |
| GO:0048667 | cell morphogenesis involved in neuron di... | 411276;552187;408956;413837;725563;406139;552782;409643;727092;408447;725681;551433;408716;409611;413557;408894;413558;413370;409000;725503;409817;411494;724545;726936;411158;410107;725910;409082;410686;408661;408435;410655;411212;412641;411201;411264;410723;726017;724281;408718;409676;726405;552742;410499;408688;726842;410694;409001;725140;551914 | 7.70E-07 |
| GO:0050896 | response to stimulus | 409667;411276;726331;408450;552187;413759;413616;411534;413837;725485;726710;725563;406139;552177;552782;412801;100578754;410718;551736;412115;726230;410393;409341;409847;552075;100576129;100577833;100578450;552309;727092;408841;410757;551796;408447;725681;551433;408716;409611;410087;725928;410006;413557;408894;552062;412008;413558;406082;412197;413370;409565;412278;412353;409000;100577214;408509;552721;725503;409817;413242;726671;409039;411113;410689;411494;724545;726936;411158;408543;724488;409082;551758;408937;552370;100577778;100578818;727479;410686;411617;726461;408661;408435;725996;408791;412355;410655;551449;725159;408557;726003;551636;726651;411212;411201;409193;408614;552711;409856;411264;410723;409255;409782;409579;408438;551930;409063;410632;726017;410956;413908;408718;409676;414017;412003;726405;552742;724853;410499;408688;408645;411181;410982;408481;726842;410161;410694;724607;408879;413133;409829;413332;100576326;409001;725140;726053;410710;406096;551914;408963 | 8.70E-07 |
| GO:0023052 | signaling | 409667;726331;408450;552187;413616;409376;411534;413837;725485;406139;552177;412801;100578754;410718;551736;412115;726230;409341;552075;100576129;100577833;409795;100578450;408576;552309;410060;408841;725681;409138;409611;725928;411693;410006;408894;552062;412008;413558;406082;413370;412278;412353;100577214;552721;725503;409817;413242;726671;411113;410689;411494;726936;411158;408937;552370;100577778;100578818;409003;410686;411617;726461;725996;408791;412355;551449;725159;726003;551636;411212;411201;409193;100578281;408614;409856;410723;409255;408438;551930;410632;410956;413908;408718;409676;414017;409957;412003;413968;726405;551214;724853;410499;408688;408645;411181;410982;410161;410694;724607;408879;413133;100576326;725140;726053;406096;408963 | 9.00E-07 |

**Supplemental Table S5.** List of GO data for combined 96hQ ChIP-seq and RNA-seq differences.

| **GO.ID** | **Term** | **Gene IDs** | **classicFisher** |
| --- | --- | --- | --- |
| GO:0006412 | translation | 725062;552106;552649;100576457;412549;726874;552152;552025;552056;409589;725357;100578551;552768;552272;409552;100576960;552774;725147;725197;725136;413868;551418;552266;409832;406099;724162;551330;409866;411380;550715;726171;551107;552726 | 1.20E-14 |
| GO:0002181 | cytoplasmic translation | 552106;100576457;409589;552272;409552;552774;725147;413868;551418;552266;409832;406099;551330;411380;550715;726171;551107;552726 | 1.30E-13 |
| GO:0043043 | peptide biosynthetic process | 725062;552106;552649;100576457;412549;726874;552152;552025;552056;409589;725357;100578551;552768;552272;726899;409552;100576960;552774;725147;725197;725136;413868;551418;552266;409832;406099;724162;551330;409866;411380;550715;726171;551107;552726 | 2.70E-13 |
| GO:0043604 | amide biosynthetic process | 725062;552106;552649;100576457;412549;726874;552152;552025;552056;409589;725357;100578551;552768;552272;726899;409552;100576960;552774;725147;725197;725136;413868;551418;552266;409832;406099;724162;551330;409866;411380;550715;726171;551107;552726 | 5.90E-13 |
| GO:0043603 | cellular amide metabolic process | 725062;552106;552649;100576457;412549;552118;726874;552152;552025;552056;409589;725357;100578551;552768;552272;726899;409552;100576960;552774;725147;725197;725136;413868;551418;552266;409832;406099;724162;551330;409866;411380;550715;726171;551107;552736;552726 | 8.70E-13 |
| GO:0006518 | peptide metabolic process | 725062;552106;552649;100576457;412549;552118;726874;552152;552025;552056;409589;725357;100578551;552768;552272;726899;409552;100576960;552774;725147;725197;725136;413868;551418;552266;409832;406099;724162;551330;409866;411380;550715;726171;551107;552726 | 9.50E-13 |
| GO:1901566 | organonitrogen compound biosynthetic pro... | 725062;552106;552649;100576457;412549;552118;726874;552152;552025;411719;552056;409589;725357;100578551;552768;552272;727483;726899;409552;100576960;552774;725147;725197;725136;552699;413868;551418;552266;409832;406099;724162;551330;409866;411380;550715;726171;551107;552084;413078;550703;552726 | 2.60E-12 |
| GO:1901564 | organonitrogen compound metabolic proces... | 725062;552106;552649;100576457;412549;552118;726874;552152;552025;411719;552056;409589;725357;100578551;552768;552272;727483;726899;409552;100576960;552774;551866;725147;725197;725136;552699;413868;551418;552266;409832;406099;724162;552023;551330;409866;411380;550715;552007;726171;551107;552736;552084;413078;550703;552726 | 1.20E-09 |
| GO:0000027 | ribosomal large subunit assembly | 100576457;724203;409589;551311;550715;726171 | 3.90E-07 |
| GO:0042255 | ribosome assembly | 100576457;724203;409589;551311;409552;550715;726171 | 1.20E-06 |
| GO:0042254 | ribosome biogenesis | 726812;100576457;552280;724203;409589;551311;411603;409552;413868;413720;724162;550715;726171;552726 | 1.60E-06 |
| GO:0042273 | ribosomal large subunit biogenesis | 100576457;552280;724203;409589;551311;413868;550715;726171 | 3.70E-06 |
| GO:0044271 | cellular nitrogen compound biosynthetic ... | 726812;725062;552106;552649;100576457;412549;552118;726874;552152;410305;552025;411719;552056;409589;725357;100578551;725719;552768;552272;727483;724435;726899;409552;100576960;412580;552774;411415;725147;725197;725136;552699;413868;551418;552266;409832;406099;724162;551330;409866;411380;552704;550715;726171;551107;552084;550703;552726 | 2.10E-05 |
| GO:0034641 | cellular nitrogen compound metabolic pro... | 726812;413727;725062;552106;552649;100576457;412549;552118;726874;552152;410305;550935;724628;552025;411719;552056;409589;725357;100578551;551311;411603;725719;552768;413815;413250;552272;727483;552662;724435;726899;409552;100576960;412580;552774;411415;551866;725147;725197;725136;552699;413868;551418;552266;409832;413720;406099;724162;552023;551330;409866;411380;552704;550715;552260;552007;726171;551107;552736;552084;550703;552726;552284 | 2.20E-05 |
| GO:0022613 | ribonucleoprotein complex biogenesis | 726812;100576457;552280;724203;409589;551311;411603;409552;413868;413720;724162;550715;552260;726171;552726 | 2.50E-05 |
| GO:0010467 | gene expression | 726812;725062;552106;552649;100576457;412549;726874;552152;410305;550935;724628;552175;552025;411719;552056;409589;725357;100578551;551311;411603;725719;552768;413815;413250;552272;724435;726899;409552;100576960;412580;552774;411415;725147;725197;725136;413868;551418;552266;409832;413720;406099;724162;551330;409866;411380;552704;550715;552260;726171;551107;552726;552284 | 3.60E-05 |
| GO:0009058 | biosynthetic process | 726812;413746;725062;552106;552649;100576457;412549;552118;726874;552152;410305;410763;552025;411719;552056;552163;409589;725357;100578551;552769;725719;552768;552272;727483;724435;726899;409552;100576960;412580;552774;411415;551866;725147;725197;725136;552699;413868;551418;552266;409832;406099;724534;724162;551330;409866;411380;552704;550715;726171;551107;552736;552084;413078;550703;552726 | 7.80E-05 |
| GO:1901576 | organic substance biosynthetic process | 726812;413746;725062;552106;552649;100576457;412549;552118;726874;552152;410305;552025;411719;552056;552163;409589;725357;100578551;552769;725719;552768;552272;727483;724435;726899;409552;100576960;412580;552774;411415;551866;725147;725197;725136;552699;413868;551418;552266;409832;406099;724534;724162;551330;409866;411380;552704;550715;726171;551107;552736;552084;413078;550703;552726 | 9.00E-05 |
| GO:0006807 | nitrogen compound metabolic process | 726812;413727;725062;552106;552649;100576457;412549;552118;726874;552152;410305;550935;724628;552025;411719;552056;409589;725357;100578551;551311;411603;725719;552768;413815;413250;552272;727483;552662;724435;726899;409552;100576960;412580;552774;411415;551866;725147;725197;725136;552699;413868;551418;552266;409832;413720;406099;724162;552023;551330;409866;411380;552704;550715;552260;552007;726171;551107;552736;552084;413078;550703;552726;552284 | 0.00012 |
| GO:0044267 | cellular protein metabolic process | 725976;725062;552106;552649;100576457;412549;726874;552152;410305;724628;410763;552175;552025;552056;409589;725357;100578551;552769;552768;552272;726899;409552;100576960;412580;552774;725147;725197;100578243;725136;551956;413868;551418;552266;409832;406099;724534;724162;551330;409866;411380;550715;409308;726171;551107;552614;552726 | 0.00015 |
| GO:0044249 | cellular biosynthetic process | 726812;413746;725062;552106;552649;100576457;412549;552118;726874;552152;410305;552025;411719;552056;552163;409589;725357;100578551;552769;725719;552768;552272;727483;724435;726899;409552;100576960;412580;552774;411415;551866;725147;725197;725136;552699;413868;551418;552266;409832;406099;724534;724162;551330;409866;411380;552704;550715;726171;551107;552084;413078;550703;552726 | 0.00018 |
| GO:0034660 | ncRNA metabolic process | 726812;726874;552025;411719;551311;411603;552768;413250;552662;100576960;725136;413868;413720;724162 | 0.0002 |
| GO:0032543 | mitochondrial translation | 725062;412549;552152;552025;725357;100578551;100576960;724162 | 0.00057 |
| GO:0022618 | ribonucleoprotein complex assembly | 100576457;724203;409589;551311;409552;550715;552260;726171 | 0.00072 |
| GO:0071826 | ribonucleoprotein complex subunit organi... | 100576457;724203;409589;551311;409552;550715;552260;726171 | 0.00107 |
| GO:0034645 | cellular macromolecule biosynthetic proc... | 726812;725062;552106;552649;100576457;412549;726874;552152;410305;552025;552056;409589;725357;100578551;725719;552768;552272;724435;409552;100576960;412580;552774;411415;725147;725197;725136;413868;551418;552266;409832;406099;724534;724162;551330;409866;411380;552704;550715;726171;551107;552726 | 0.00115 |
| GO:0019538 | protein metabolic process | 725976;725062;552106;552649;100576457;412549;726874;552152;410305;724628;410763;552175;552025;552056;409589;725357;100578551;552769;552768;552272;726899;409552;100576960;412580;552774;725147;725197;100578243;725136;551956;413868;551418;552266;409832;406099;724534;724162;551330;409866;411380;550715;409308;726171;551107;552614;552726 | 0.00161 |
| GO:0070127 | tRNA aminoacylation for mitochondrial pr... | 552025;100576960 | 0.00178 |
| GO:0009059 | macromolecule biosynthetic process | 726812;725062;552106;552649;100576457;412549;726874;552152;410305;552025;552056;409589;725357;100578551;725719;552768;552272;724435;409552;100576960;412580;552774;411415;725147;725197;725136;413868;551418;552266;409832;406099;724534;724162;551330;409866;411380;552704;550715;726171;551107;552726 | 0.00188 |
| GO:0000959 | mitochondrial RNA metabolic process | 726874;552025;100576960 | 0.00248 |

**Supplemental Table S6.** List of GO data for combined 96hW ChIP-seq and RNA-seq differences.

| **GO.ID** | **Term** | **Gene IDs** | **classicFisher** |
| --- | --- | --- | --- |
| GO:0048731 | system development | 411276;552187;552782;409643;100576129;411569;409611;725928;408509;413242;726419;410689;100578818;726461;408661;409856;410723;408438;410956;413968;726842;724607;409001;408792;410257;724595;413569;413683;408763;551733;413125;551071;551746;410685;413377;410351;408777;727000;408810;727235;100578826;410279;408835;725754;408786;408869;100576251;408890;552094;410739;100576449;552617;724991;408298;409902;413288;412739;411250;412108;408264;724973;726469;413215;413677;408430;725551;408431;411981;410674;724929;408278;724207;411112;412256;409084;724902;406084;410497;410785;409893;726709;409348 | 3.40E-14 |
| GO:0007399 | nervous system development | 411276;552187;552782;409643;100576129;409611;725928;408509;726419;410689;726461;408661;410723;410956;726842;724607;409001;408792;724595;413569;408763;551733;551071;551746;410685;413377;410351;408777;727000;408810;727235;408835;725754;408786;408869;100576251;408890;552094;410739;100576449;552617;724991;408298;409902;413288;412739;411250;412108;408264;724973;413215;408430;410674;724929;408278;724207;411112;412256;724902;406084;410497;410785;409893;726709;409348 | 6.60E-13 |
| GO:0048513 | animal organ development | 411276;100576129;411569;409611;725928;413242;410689;100578818;408661;409856;410723;410956;413968;726842;724607;409001;408792;410257;724595;413569;413683;408763;551733;413125;551746;410685;413377;410351;408810;727235;100578826;410279;725754;408786;408869;100576251;552094;410739;100576449;724991;409902;413288;412739;411250;412108;408264;726469;413215;413677;725551;408431;411981;724929;408278;412256;409084;724902;406084;410497;726709;409348 | 1.20E-10 |
| GO:0048699 | generation of neurons | 411276;552187;552782;409643;409611;725928;726419;408661;410723;726842;724607;409001;408792;724595;413569;551733;551746;410685;413377;410351;408777;727000;408810;727235;408835;725754;408786;408869;100576251;408890;410739;100576449;552617;408298;409902;412739;411250;412108;408264;413215;408430;410674;724929;408278;724207;411112;412256;724902;406084;410497;410785;409893;726709;409348 | 1.30E-10 |
| GO:0030182 | neuron differentiation | 411276;552187;552782;409643;409611;725928;726419;408661;410723;726842;724607;409001;408792;724595;413569;551733;551746;410685;413377;410351;408777;727000;408810;727235;408835;408786;408869;100576251;408890;410739;100576449;552617;408298;409902;412739;411250;412108;408264;413215;408430;410674;724929;408278;724207;411112;412256;724902;406084;410785;409893;726709 | 4.20E-10 |
| GO:0007275 | multicellular organism development | 411276;552187;552782;409643;100576129;411569;409611;725928;408509;413242;726419;410689;100578818;726461;408661;409856;410723;408438;410956;413968;726842;724607;409001;408792;410257;724595;413569;727238;413683;408763;551733;413125;551071;551746;410685;413377;410351;408777;727000;408810;727235;100578826;410279;408835;725754;408786;408869;100576251;408890;552094;410739;100576449;552617;724991;408298;409902;413288;412739;411250;412108;408264;724973;726469;413215;413677;408430;725551;408431;411981;410674;724929;408278;724207;411112;412256;409084;724902;406084;410497;410785;409893;726709;409348 | 4.50E-10 |
| GO:0048869 | cellular developmental process | 411276;552187;552782;409643;411569;409611;725928;726419;410689;100578818;408661;409856;410723;408438;726842;724607;409001;408792;410257;412878;724595;413569;727238;408763;551733;724460;551746;410685;413377;100577280;410351;408777;727000;408810;727235;410279;408835;411575;725754;408786;408869;100576251;408890;552094;410739;100576449;552617;724991;408298;409902;412739;411250;412108;408264;724973;726469;413215;408591;408430;408431;411981;410674;724929;408278;724207;411112;412256;724902;406084;410497;410785;409893;726709;409348 | 6.50E-10 |
| GO:0048856 | anatomical structure development | 411276;552187;552782;409643;100576129;411569;409611;725928;408509;413242;726419;410689;100578818;726461;408661;409856;410723;408438;410956;413968;726842;724607;409001;408792;410257;412878;724595;413569;727238;413683;408763;551733;413125;551071;551746;410685;413377;100577280;410351;408777;727000;408810;727235;100578826;410279;408835;411575;725754;408786;408869;100576251;408890;552094;410739;100576449;552617;724991;408298;409902;413288;412739;411250;412108;408264;724973;726469;413215;408591;413677;408430;725551;408431;411981;410674;724929;408278;724207;411112;412256;409084;724902;406084;410497;410785;409893;726709;409348 | 6.80E-10 |
| GO:0022008 | neurogenesis | 411276;552187;552782;409643;409611;725928;726419;408661;410723;726842;724607;409001;408792;724595;413569;551733;551746;410685;413377;410351;408777;727000;408810;727235;408835;725754;408786;408869;100576251;408890;410739;100576449;552617;408298;409902;412739;411250;412108;408264;413215;408430;410674;724929;408278;724207;411112;412256;724902;406084;410497;410785;409893;726709;409348 | 7.40E-10 |
| GO:0048468 | cell development | 411276;552187;552782;409643;411569;409611;725928;726419;410689;408661;409856;410723;408438;726842;724607;409001;408792;410257;412878;724595;413569;727238;408763;551733;551746;410685;413377;100577280;410351;408777;727000;408810;727235;410279;408835;725754;408786;408869;100576251;408890;410739;100576449;552617;724991;408298;409902;412739;411250;408264;726469;413215;408591;408430;408431;410674;724929;408278;724207;411112;412256;724902;406084;410785;409893;726709;409348 | 8.40E-10 |
| GO:0030154 | cell differentiation | 411276;552187;552782;409643;411569;409611;725928;726419;410689;100578818;408661;409856;410723;408438;726842;724607;409001;408792;410257;412878;724595;413569;727238;408763;551733;724460;551746;410685;413377;100577280;410351;408777;727000;408810;727235;410279;408835;725754;408786;408869;100576251;408890;552094;410739;100576449;552617;724991;408298;409902;412739;411250;412108;408264;724973;726469;413215;408591;408430;408431;411981;410674;724929;408278;724207;411112;412256;724902;406084;410497;410785;409893;726709;409348 | 1.10E-09 |
| GO:0048666 | neuron development | 411276;552187;552782;409643;409611;726419;408661;410723;726842;724607;409001;724595;413569;551733;551746;410685;413377;408777;727000;408810;727235;408835;408786;408869;100576251;408890;410739;100576449;552617;408298;409902;412739;411250;408264;413215;408430;410674;724929;408278;724207;411112;412256;724902;406084;410785;409893;726709 | 1.40E-09 |
| GO:0009653 | anatomical structure morphogenesis | 411276;552187;552782;409643;411569;409611;725928;413242;410689;408661;409856;410723;408438;410956;413968;726842;724607;409001;408792;410257;724595;413569;413683;408763;551733;413125;551746;410685;413377;410351;408777;727000;408810;727235;100578826;410279;411575;408786;408869;408890;410739;100576449;552617;724991;408298;409902;412739;411250;412108;408264;726469;413215;408591;413677;411981;410674;724929;408278;724207;411112;412256;409084;406084;410497;410785;726709;409348 | 1.50E-09 |
| GO:0032502 | developmental process | 411276;552187;411534;552782;409643;100576129;411569;409611;725928;408509;413242;726419;410689;100578818;726461;408661;409856;410723;408438;410956;413968;726842;724607;409001;408792;410257;412878;724595;413569;727238;413683;408763;551733;413125;724460;551071;551746;410685;413377;100577280;410351;408777;727000;408810;727235;100578826;410279;408835;411575;725754;408786;408869;100576251;408890;552094;410739;100576449;552617;724991;408298;409902;413288;412739;411250;412108;408264;724973;726469;413215;408591;413677;408430;725551;408431;411981;410674;724929;408278;724207;411112;412256;409084;724902;406084;410497;410785;409893;726709;409348 | 2.80E-09 |
| GO:0009887 | animal organ morphogenesis | 411276;409611;725928;413242;408661;410723;410956;413968;726842;724607;408792;410257;724595;413569;413683;408763;413125;551746;410685;413377;410351;408810;727235;100578826;408869;410739;100576449;724991;409902;412739;411250;412108;408264;726469;413215;413677;411981;724929;408278;409084;406084;410497;726709;409348 | 3.40E-09 |
| GO:0044767 | single-organism developmental process | 411276;552187;552782;409643;100576129;411569;409611;725928;408509;413242;726419;410689;100578818;726461;408661;409856;410723;408438;410956;413968;726842;724607;409001;408792;410257;412878;724595;413569;727238;413683;408763;551733;413125;724460;551071;551746;410685;413377;100577280;410351;408777;727000;408810;727235;100578826;410279;408835;411575;725754;408786;408869;100576251;408890;552094;410739;100576449;552617;724991;408298;409902;413288;412739;411250;412108;408264;724973;726469;413215;408591;413677;408430;725551;408431;411981;410674;724929;408278;724207;411112;412256;409084;724902;406084;410497;410785;409893;726709;409348 | 4.80E-09 |
| GO:0000902 | cell morphogenesis | 411276;552187;552782;409643;409611;725928;408661;410723;408438;726842;724607;409001;724595;413569;551733;551746;410685;410351;408777;727000;408810;727235;411575;408786;408869;408890;410739;100576449;552617;408298;409902;412739;412108;408264;413215;410674;724929;408278;724207;411112;412256;406084;410785;409348 | 5.00E-09 |
| GO:0044707 | single-multicellular organism process | 411276;552187;552782;726230;409643;100576129;411569;409611;725928;408509;413242;726419;410689;408543;100578818;726461;408661;725996;409856;410723;408438;410956;413968;726842;724607;409001;408792;410257;724595;413569;727238;413683;408763;551733;413125;551071;551746;410685;413377;410351;408777;727000;408810;727235;100578826;410279;408835;725754;408786;408869;100576251;408890;552094;410739;100576449;552617;724991;408298;409902;413288;412739;411250;412108;408264;724973;726469;413215;413677;408430;725551;408431;411981;410674;724929;408278;724207;411112;412256;409084;724902;406084;410497;410785;409893;726709;552142;409348 | 8.60E-09 |
| GO:0050794 | regulation of cellular process | 411276;552187;411534;552782;726230;100576129;409611;725928;413242;410689;100578818;726461;408661;725996;409856;410723;408438;410956;413968;726842;724607;409001;406096;412878;724595;413569;727238;413683;408763;551733;413125;409027;551071;551746;410685;409337;413377;100577280;410351;724442;408777;727000;408810;727235;100578826;410279;408835;411575;725754;725662;408786;408869;408890;552094;410739;100576449;552617;724991;408298;409902;413288;412739;411250;412108;408264;724973;726469;725393;413215;408591;413677;408430;725551;408431;411981;724929;408278;412256;409084;552585;724902;406084;410497;410785;726709;725061;552142;409348;410322 | 1.00E-08 |
| GO:0032989 | cellular component morphogenesis | 411276;552187;552782;409643;411569;409611;725928;408661;409856;410723;408438;726842;724607;409001;724595;413569;551733;551746;410685;410351;408777;727000;408810;727235;411575;408786;408869;408890;410739;100576449;552617;408298;409902;412739;412108;408264;413215;410674;724929;408278;724207;411112;412256;406084;410785;409348 | 1.90E-08 |
| GO:0032501 | multicellular organismal process | 411276;552187;552782;726230;409643;100576129;411569;409611;725928;408509;413242;726419;410689;408543;100578818;726461;408661;725996;409856;410723;408438;410956;413968;726842;724607;409001;408792;410257;412878;724595;412917;413569;727238;413683;408763;551733;413125;551071;551746;410685;413377;100577280;410351;408777;727000;408810;727235;100578826;410279;408835;725754;408786;408869;100576251;408890;552094;725015;410739;100576449;552617;724991;408298;409902;413288;412739;411250;412108;408264;724973;726469;413215;408591;413677;408430;725551;408431;411981;410674;724929;408278;724207;411112;412256;409084;724902;406084;410497;410785;409893;726709;552142;409348 | 3.70E-08 |
| GO:0050793 | regulation of developmental process | 411534;552782;100576129;409611;413242;100578818;410723;408438;410956;726842;724607;409001;724595;413569;408763;551746;410351;408777;408810;727235;410279;411575;725754;408786;408869;552094;100576449;552617;724991;408298;409902;412739;412108;724973;408431;724929;406084;410497;410785;726709;409348 | 4.90E-08 |
| GO:0048523 | negative regulation of cellular process | 552187;411534;100576129;410689;408661;410723;410956;726842;724607;724595;413569;727238;551733;551746;410685;413377;100577280;410351;408777;727235;725754;725662;408869;408890;552094;100576449;552617;724991;408298;413288;411250;412108;724973;726469;725393;408591;725551;408431;411981;724929;408278;412256;409084;552585;724902;406084;410785;726709;409348 | 8.00E-08 |
| GO:0000904 | cell morphogenesis involved in different... | 411276;552187;552782;409643;409611;408661;410723;726842;724607;409001;724595;413569;551746;410685;410351;408777;727000;727235;408786;408869;408890;410739;100576449;552617;408298;409902;412739;408264;413215;410674;724929;408278;412256;406084;410785 | 1.30E-07 |
| GO:0003002 | regionalization | 413242;408661;410723;410956;413968;726842;724607;408792;408763;413125;551746;410351;727235;100578826;410279;408869;100576449;724991;408298;412739;411250;408264;726469;725551;411981;409084;724902;406084;726709;409348 | 2.80E-07 |
| GO:0048667 | cell morphogenesis involved in neuron di... | 411276;552187;552782;409643;409611;408661;410723;726842;409001;724595;413569;551746;410685;408777;727000;727235;408786;408869;408890;410739;100576449;552617;408298;409902;412739;408264;413215;410674;724929;408278;412256;406084;410785 | 3.00E-07 |
| GO:0040011 | locomotion | 411569;409611;725928;413242;408661;410723;410956;408481;726842;724607;409001;413569;410685;410351;408777;727000;408810;727235;408786;408869;410739;100576449;552617;408298;409902;412739;408264;413215;411981;410674;724929;408278;406084;410497;410785;409893;726709 | 3.10E-07 |
| GO:0048589 | developmental growth | 409611;413242;410689;100578818;726461;408661;408438;410956;726842;724607;409001;724595;413569;408777;727000;727235;408786;552094;410739;552617;724991;408298;409902;412108;724973;408431;724929;410785 | 3.80E-07 |
| GO:0040007 | growth | 409611;725928;413242;410689;100578818;726461;408661;408438;410956;726842;724607;409001;724595;413569;408777;727000;727235;408786;552094;410739;552617;724991;408298;409902;412108;724973;408431;724929;406084;410785;409348 | 3.90E-07 |
| GO:0048812 | neuron projection morphogenesis | 411276;552187;552782;409643;409611;408661;410723;726842;724607;409001;724595;413569;551733;551746;410685;408777;727000;727235;408786;408869;408890;410739;100576449;552617;408298;409902;412739;408264;413215;410674;724929;408278;724207;411112;412256;406084;410785 | 4.20E-07 |

**Supplemental Table S7.** Detailed mapping statistics for ChIP-seq.

| **Sample** | **Histone** | **Replicate** | **Sequenced Reads** | **Mapped Reads** | **Percentage** |
| --- | --- | --- | --- | --- | --- |
| 96hQ | H3K4me3 | R1 | 34,669,609 | 30,581,396 | 88 |
|  |  | R2 | 57,935,572 | 51,722,279 | 89 |
| 96hW | H3K4me3 | R1 | 63,087,388 | 54,471,413 | 86 |
|  |  | R2 | 57,935,572 | 51,722,279 | 89 |
| 96hQ | H3K27ac | R1 | 41,510,735 | 36,093,006 | 87 |
|  |  | R2 | 38,522,770 | 33,509,382 | 87 |
| 96hW | H3K27ac | R1 | 51,243,102 | 45,499,616 | 89 |
|  |  | R2 | 55,182,941 | 48,643,044 | 88 |
| 96hQ | H3K36me3 | R1 | 37,220,378 | 33,136,156 | 89 |
|  |  | R2 | 38,753,123 | 35,224,118 | 90 |
| 96hW | H3K36me3 | R1 | 55,885,087 | 50,894,564 | 91 |
|  |  | R2 | 57,239,224 | 52,685,185 | 92 |
| 96hQ | Input |  | 31437953 | 25221473 | 80 |
| 96hW | Input |  | 72446922 | 51067663 | 70 |

**Supplemental Table S8.** Detailed mapping statistics for RNA-seq.

| **Samples** | **Number of Reads** | **Number of Reads Used** | **Percentage** |
| --- | --- | --- | --- |
| 96hQ | 26,872,290 | 22,057,972 | 82 |
| 96hQ | 28,095,222 | 22,478,870 | 80 |
| 96hQ | 28,627,449 | 23,037,945 | 80 |
| 96hQ | 28,987,668 | 23,344,818 | 81 |
| 96hW | 24,400,160 | 19,139,730 | 78 |
| 96hW | 28,050,319 | 21,848,473 | 78 |
| 96hW | 25,758,401 | 20,095,458 | 78 |
| 96hW | 27,281,147 | 21,545,316 | 79 |
